# Neural Population Tuning Links Visual Cortical Anatomy to Human Visual Perception

**DOI:** 10.1016/j.neuron.2014.12.041

**Published:** 2015-02-04

**Authors:** Chen Song, Dietrich Samuel Schwarzkopf, Ryota Kanai, Geraint Rees

**Affiliations:** 1 Institute of Cognitive Neuroscience, University College London, 17 Queen Square, London WC1N 3AR, UK; 2 Wellcome Trust Centre for Neuroimaging, University College London, 12 Queen Square, London WC1N 3BG, UK; 3 School of Psychology, University of Sussex, Sussex House, Brighton BN1 9QH, UK

## Abstract

The anatomy of cerebral cortex is characterized by two genetically independent variables, cortical thickness and cortical surface area, that jointly determine cortical volume. It remains unclear how cortical anatomy might influence neural response properties and whether such influences would have behavioral consequences. Here, we report that thickness and surface area of human early visual cortices exert opposite influences on neural population tuning with behavioral consequences for perceptual acuity. We found that visual cortical thickness correlated negatively with the sharpness of neural population tuning and the accuracy of perceptual discrimination at different visual field positions. In contrast, visual cortical surface area correlated positively with neural population tuning sharpness and perceptual discrimination accuracy. Our findings reveal a central role for neural population tuning in linking visual cortical anatomy to visual perception and suggest that a perceptually advantageous visual cortex is a thinned one with an enlarged surface area.

## Highlights

• Variability in cortical thickness and surface area has opposite functional impacts
• Smaller human visual cortical thickness links to high neural and perceptual acuity
• Larger human visual cortical surface area links to high neural and perceptual acuity

## Teaser

Song et al. showed that large brains are not necessarily advantageous. Instead, the two dimensions, thickness and surface area, of human brain have opposite impacts on visual perception. A perceptually advantageous brain is a thinned one with enlarged surface area.

## Introduction

The cerebral cortex is a neural sheet composed vertically of ontogenetic cortical columns and horizontally of laminar layers (Rakic, 1988; Mountcastle, 1997). The surface area of cerebral cortex depends on the proliferation of cortical columns, whereas the thickness of cerebral cortex depends on the generation of laminar layers (Rakic, 1974; Bugbee and Goldman-Rakic, 1983; Jones, 2000). These two elementary dimensions, cortical thickness and cortical surface area, jointly determine cortical volume. However, controlled by independent sets of genetic-developmental factors, cortical thickness and cortical surface area exhibit distinct patterns of variability (Rakic, 1988; Panizzon et al., 2009; Joyner et al., 2009; Chen et al., 2011). Cortical surface area has expanded over 1,000-fold from small mammals to humans (Blinkov and Glezer, 1968; Rakic, 1988). Even within the human species, the surface area of a cortical region, such as visual cortical surface area, can vary up to 3-fold across healthy adults (Dougherty et al., 2003). By contrast, cortical thickness has only doubled during mammalian evolution and differs marginally across human individuals (Blinkov and Glezer, 1968; Rakic, 1988). Nevertheless, cortical thickness can vary over 3-fold across different cortical locations within the same cortical region of the same individual (Fischl and Dale, 2000; Hilgetag and Barbas, 2006).

This substantial variability in cortical anatomy has attracted great interest in the study of its behavioral consequences, and recent progress has been made in identifying correlations between higher performance on a variety of behavioral tasks and larger local cortical volume in task-relevant cortical regions (Kanai and Rees, 2011). However, the fundamental questions of whether a larger cortical volume is in essence behaviorally advantageous and why cortical volume is behaviorally relevant remain to be addressed. An intuitive hypothesis is that increases in cortical volume, arising either from increased cortical thickness or cortical surface area, improve behavioral performance by engaging responses from more neurons (Haug, 1987) and increasing the overall signal-to-noise ratio (Gilbert et al., 2001). Alternatively, changes in behavioral performance may be driven by changes in neural response properties that are associated with variation in local cortical volume and potentially associated differently with cortical thickness versus cortical surface area, as these two anatomical dimensions exhibit distinct natures that affect different aspects of intracortical processing (Kaas, 2000). Specifically, cortical thickness characterizes local (point-level) cortical anatomy, where the thickness at different cortical locations within a cortical region can be independently assessed and reflects the result of tissue proliferation. By contrast, cortical surface area characterizes global (region-level) cortical anatomy, where the surface area of a cortical region is determined jointly by the set of cortical locations it bounds and reflects the result of cortical arealization. As such, variability in the surface area of a cortical region might globally influence all the cortical columns within that region and intercolumnar processing between them, whereas variability in the thickness at a cortical location might locally influence the cortical column at that location and interlaminar processing within it.

To test our two hypotheses, we therefore investigated whether cortical thickness and cortical surface area, which both contribute to cortical volume, had similar or different functional impacts for neural response properties and human behavioral performance. In human cerebral cortex, the neural response properties of many cortical regions are hard to characterize using noninvasive neuroimaging techniques. Due to the limited spatial resolution of fMRI signals, different neurons within a single fMRI voxel tend to exhibit heterogeneous response properties that often render the voxel-level characterization of neural responses qualitatively different from the response properties of single neurons. An exception to this limitation is early retinotopic visual cortices. Neurons in early visual cortices respond to visual field position in an orderly fashion, where cortically adjacent neurons are tuned to spatially adjacent visual field positions (Hubel and Wiesel, 1974; Sereno et al., 1995). This relative similarity in tuning responses between different neurons within a single fMRI voxel allows fMRI-based characterization of neural population tuning (Dumoulin and Wandell, 2008; Fischer and Whitney, 2009). Given the close correspondence between neural tuning properties and perceptual discrimination performance (Purushothaman and Bradley, 2005), this fMRI-based characterization of neural population tuning enabled us to explore the behavioral significance of any influence that cortical anatomy may have on neural response properties.

Using human early visual cortices (V1 and V2) as a model system, we investigated how visual cortical thickness and visual cortical surface area influenced neural population tuning for visual field position, and whether such influences had behavioral consequences on perceptual discrimination for visual field position. Since visual cortical thickness captures the differences between visual field positions in the cortical architecture at corresponding visual cortical locations, we studied how thickness at different visual cortical locations related to the width of neural population tuning and the0 of perceptual discrimination for corresponding visual field positions. On the other hand, since visual cortical surface area reflects the differences between individuals in the proportion of cortex devoted to early visual processing, we studied how surface area of early visual cortices influenced the position tuning width and position discrimination threshold across the visual field in general.

## Results

For a group of 20 healthy participants, we used structural MRI, high-resolution fMRI, and visual psychophysics to measure anatomy of early visual cortices (V1 and V2), population tuning of visual cortical neurons, and performance in perceptual discrimination, respectively. The psychophysical experiments assessing perceptual discrimination were conducted outside the scanner, while the neuroimaging experiments assessing visual cortical anatomy and neural population tuning were performed inside the scanner. During data analysis, we explored the relationships among these independent measures reflecting cortical anatomy, neural response properties, and behavioral performance, respectively.

### Variability in Visual Cortical Anatomy

Delineation of early visual cortices (V1 and V2) used the standard method of retinotopic mapping (Sereno et al., 1995). The mapped visual field covered an eccentricity range from 0.25 to 7.2 degree of visual angle. To improve the delineation accuracy of polar angle boundaries (representing vertical and horizontal meridians), we conducted two different retinotopic mapping experiments using phase-encoded paradigm (Sereno et al., 1995) and population-receptive-field paradigm (Dumoulin and Wandell, 2008), respectively. To improve the delineation accuracy of eccentricity boundaries (representing 0.25 and 7.2 degree eccentricity), the eccentricity boundaries delineated from the two retinotopic mapping experiments were refined in a third experiment using a retinotopic localizer. The performance of retinotopic-based delineation was assessed through comparison with morphology-based delineation, where the medial occipital cortex was segmented according to the cortical folding patterns (Desikan et al., 2006). We found that the delineation of early visual cortices was consistent across different delineation protocols (Figure S1 available online; Supplemental Experimental Procedures section 2).

After the delineation of early visual cortices, we measured visual cortical thickness and visual cortical surface area by applying the surface-based analysis on the structural MRI data collected using the standard T1-weighted MRI sequence. In the analysis, the structural MRI data were segmented into different cortical tissues, from which the 3D cortical surface was reconstructed in a smooth triangle-mesh model, with each vertex of this mesh representing a single cortical location distinguishable by MRI (Dale et al., 1999). Based on this 3D cortical surface reconstruction, we measured the thickness at individual visual cortical locations (vertices) and the surface area summed over different visual cortical locations. The MRI-based measure of visual cortical anatomy was potentially confounded by the choice of data analysis software. Therefore, we repeated the analysis in four established software packages, SPM (Ashburner, 2012), Freesurfer (Fischl, 2012), FSL (Jenkinson et al., 2012), and MIPAV CBS (Bazin et al., 2013), in order to separate the contributions of software specific versus software independent factors. We found that the MRI-based measure of visual cortical anatomy was not biased by the specific choice of data analysis software (Supplemental Experimental Procedures section 3.1).

Our MRI-based measure of visual cortical anatomy was also potentially vulnerable to any confounding influences of data acquisition sequence. Specifically, while the T1-weighted MRI sequence we employed is a widely used standard protocol, the signal in fact represents a combination of magnetic-field-specific and biological-tissue-specific components. As a result, the T1-weighted MRI images had inhomogenous intensity and low tissue contrast that could potentially lead to bias in the segmentation of cortical tissues. To address this limitation in quality of the standard T1-weighted MRI sequence, in control experiments, we collected the structural MRI data using a state-of-art quantitative-T1 MRI sequence, at both a high resolution (0.8 millimeter [mm] isotropic voxels) and a standard resolution (1 mm isotropic voxels). Through the detection of multiple parametric signals, the quantitative-T1 MRI sequence factored out the magnetic-field-specific component and directly reflected the physical property of the underlying biological tissue (Weiskopf et al., 2013). As such, the quantitative-T1 MRI images had homogeneous intensity and high tissue contrast that greatly reduced potential bias in the surface-based analysis (Figure S2). Although the standard T1-weighted MRI sequence had image quality limitations, we found that the MRI-based measure of visual cortical anatomy was nonetheless robust against such limitations and was strongly correlated across different data acquisition sequences (Supplemental Experimental Procedures section 3.2).

**Figure 1:**
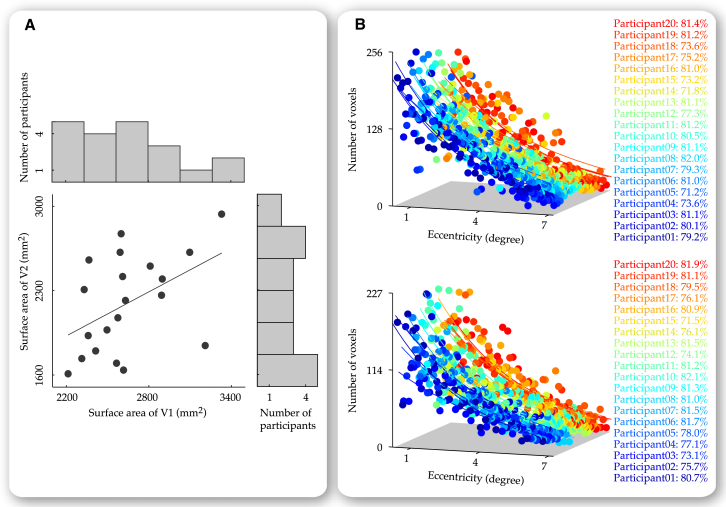
Variability in Visual Cortical Surface Area Variability in visual cortical surface area was studied in a group of 20 participants, where we applied the standard method of retinotopic mapping to delineate the part of early visual cortices (V1 and V2) that responded to the visual field between 0.25 and 7.2 degree eccentricity. Based on the retinotopy delineation, visual cortical surface area was calculated as the surface area summed over all cortical locations in the retinotopically delineated part of V1 or V2. This retinotopically delineated visual cortical surface area exhibited a 2-fold interindividual variability (illustrated in the marginal histogram of A) that was correlated between V1 and V2 (illustrated in the scatter plot of A). To quantify the fraction of retinotopically delineated V1 or V2 to full V1 or V2, the distribution of mapped visual field eccentricity was plotted on a voxel basis, where voxels responsive to similar eccentricity were binned to generate 30 data points for each participant (B). From the exponential fit to the eccentricity distribution, we estimated the retinotopically delineated V1 or V2 as the area under the exponential fit between *x* equaled 0.25 and *x* equaled 7.2, and the full V1 or V2 as the area under the exponential fit between *x* equaled 0 and *x* approximated infinite. Data points are color coded according to the participant (B). Parameters reflect the fraction of retinotopically delineated V1 or V2 (B).

From our MRI-based measure of visual cortical anatomy, we studied variability in visual cortical surface area and visual cortical thickness. Consistent with previous reports (Dougherty et al., 2003), the retinotopically delineated visual cortical surface area exhibited a 2-fold interindividual variability (Figure 1A, summed across left and right hemispheres; V1, 2,213 mm^2^ to 3,328 mm^2^ and V2, 1,611 mm^2^ to 2,936 mm^2^) that was correlated between V1 and V2 (Figure 1A; r = 0.568, p < 0.05, n = 20). As the retinotopy delineation covered a part rather than the full extent of early visual cortices, we further explored interindividual variability in the fraction of retinotopy coverage, based on the distribution of mapped visual field eccentricity derived from the eccentricity map. This distribution was best fitted with an exponential function *y = ae*^−*bx*^, which reflected the percentage of voxels responsive to each visual field eccentricity (Figure 1B). Given that different voxels were equal in volume, we estimated the retinotopically delineated part of early visual cortices as the area under the exponential curve from *x* equaled 0.25 degree eccentricity to *x* equaled 7.2 degree eccentricity, and the full extent of early visual cortices as the area under the exponential curve from *x* equaled 0 to *x* approximated infinite. We found that in both V1 and V2, the retinotopically delineated part accounted for about three-quarters of the full area. This fraction of retinotopy coverage was rather consistent across participants (V1, mean = 78.3%, SD = 3.7%, n = 20 and V2, mean = 78.8%, SD = 3.3%, n = 20) and did not correlate with the measure of visual cortical surface area (V1, r = −0.120, p = 0.564, n = 20 and V2, r = 0.080, p = 0.768, n = 20). Therefore, we concluded that the retinotopically delineated visual cortical surface area captured true anatomical variability.

**Figure 2:**
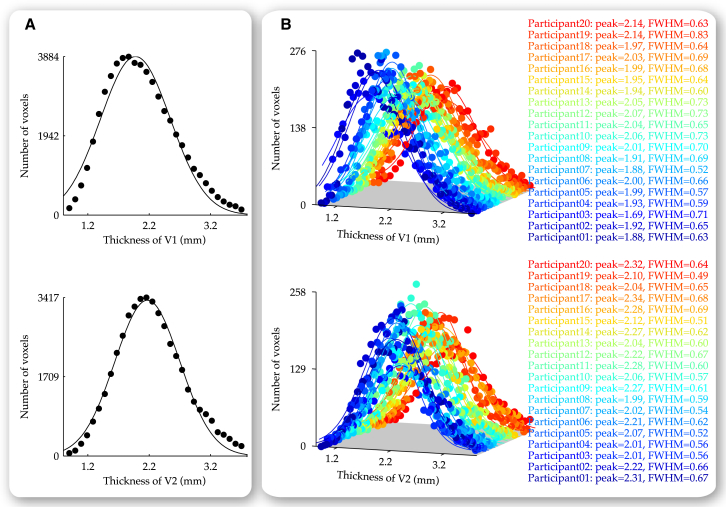
Variability in Visual Cortical Thickness Based on the retinotopy delineation of early visual cortices (V1 and V2), visual cortical thickness was calculated at individual cortical locations in the retinotopically delineated part of V1 or V2. The distribution of V1 or V2 thickness was plotted on a voxel basis, where voxels with similar thickness were binned to generate 30 data points for the group of 20 participants (A) or for each participant in the group (B). The mean and the SD of V1 or V2 thickness derived from the Gaussian fit to the thickness distribution illustrated the variability in visual cortical thickness across different visual cortical locations. Data points are color coded according to the participant (B). Parameters are derived from the Gaussian fit to the thickness distribution (B).

Compared to the large degree of interindividual variability in visual cortical surface area, the average thickness of early visual cortices varied across participants to a much smaller degree from 2 mm to 2.5 mm. Nevertheless, within individual participants, visual cortical thickness varied across different visual cortical locations from 1 mm to 4 mm following a Gaussian distribution (Figure 2). In addition to this general intraindividual variability in visual cortical thickness, we observed an intraindividual increase in visual cortical thickness from sulci to gyri and from parafovea (central 2.0 degree eccentricity) to perifovea. This increase in visual cortical thickness from parafovea to perifovea was observed for both sulci (V1, T = 6.533, p < 0.0001, n = 20 and V2, T = 8.359, p < 0.0001, n = 20) and gyri (V1, T = 6.509, p < 0.0001, n = 20 and V2, T = 8.874, p < 0.0001, n = 20). In a similar fashion, the increase in visual cortical thickness from sulci to gyri was observed for both parafovea (V1, T = 7.113, p < 0.0001, n = 20 and V2, T = 8.357, p < 0.0001, n = 20) and perifovea (V1, T = 9.972, p < 0.0001, n = 20 and V2, T = 9.471, p < 0.0001, n = 20).

While the structural MRI data allowed a noninvasive, in vivo measure of visual cortical anatomy, the measure was, at the same time, limited by its indirect nature. In contrast, a direct measure of cortical anatomy (albeit in vitro) is possible from postmortem histology. Therefore, we further addressed the reliability of our in vivo MRI measure by comparing it with an in vitro histology measure derived from postmortem human brain. Conventional analysis of histology data employs a slice-based approach that is constrained by the slice orientation and has a limited sampling coverage. For example, the slice-based measure of cortical thickness is only valid for histology slices orthogonal to the cortical surface. To overcome this limitation, we developed a surface-based approach and applied it to a data set of high-resolution (40 μm isotropic pixel) whole-brain (4,992 pixel × 3,328 pixel) histology images (502 images in total), taken consecutively every 300 μm along the dorsoventral axis of a postmortem human brain. In the analysis, we manually segmented each histology image into different cortical tissues, from which we reconstructed the 3D cortical surface and acquired the surface-based measure of visual cortical anatomy (Figure S3; Supplemental Experimental Procedures section 3.3). While time-consuming, this surface-based analysis offered a sampling coverage of the full brain that was unconstrained by the slice orientation.

From this histology-based measure of visual cortical anatomy, we observed a substantial degree of intraindividual variability in visual cortical thickness (Figure S4) that was similar in extent to the MRI-based measure (Figure 2). Moreover, the dependence of visual cortical thickness on cortical folding and visual field eccentricity that we observed in the structural MRI data was recaptured by the histology data. Specifically, we observed an increase in visual cortical thickness from sulci to gyri for both parafovea (V1, T = 13.498, p < 0.0001, n = 87,455 voxels and V2, T = 18.179, p < 0.0001, n = 86,466 voxels) and perifovea (V1, T = 23.822, p < 0.0001, n = 230,387 voxels and V2, T = 9.507, p < 0.0001, n = 50,348 voxels), as well as an increase in visual cortical thickness from parafovea to perifovea for both sulci (V1, T = 83.929, p < 0.0001, n = 146,834 voxels and V2, T = 56.089, p < 0.0001, n = 72,410 voxels) and gyri (V1, T = 97.783, p < 0.0001, n = 171,008 voxels and V2, T = 49.674, p < 0.0001, n = 64,404 voxels). The consistency with the in vitro histology measure validated our application of in vivo structural MRI data to assess visual cortical anatomy.

### Variability in Neural Population Tuning Width

Our measure of neural population tuning for visual field position was based on the established method of population-receptive-field mapping (Dumoulin and Wandell, 2008). In the experiment, a bar stimulus was presented at different visual field positions, and the BOLD time series recorded from each voxel in early visual cortices was deconvolved with a hemodynamic response function before fitting with a 2D Gaussian characterization of position tuning profile (Supplemental Experimental Procedures section 4.1). The 2D Gaussian function characterized the range of visual field positions that the voxel responded to (position tuning width) and the visual field position where the voxel responded the strongest (position tuning peak). We acquired the measure of position tuning for individual visual cortical locations (vertices) from single fMRI voxels, where the exact cortical depth of the voxels did not affect the measure (Figure S5; Supplemental Experimental Procedures section 4.2). This measure of neural population tuning reflected a combined contribution from the average position tuning width of neurons in the voxel and the heterogeneity in position tuning peak between different neurons in the voxel (Hubel and Wiesel, 1974). To improve the resolution of the measure and minimize intravoxel heterogeneity in position tuning peak, we collected the fMRI data at a high spatial resolution (1.5 mm isotropic voxel) using a 3D echo plannar imaging acquisition sequence with parallel imaging acceleration (Lutti et al., 2013). For tissue volumes as small as 1.5 mm isotropic, the heterogeneity in position tuning peak is smaller than the position tuning width of single neurons and is correlated with the average position tuning width of neurons in the tissue volume (Hubel and Wiesel, 1974). As such, the voxel-level measure of the position tuning width in effect reflected the average tuning width of neurons in the voxel. Indeed, this voxel-level position tuning width (0.6 ± 0.35 degree of visual angle) measured here in the retinotopically delineated part of human primary visual cortex was comparable with neural-level position tuning width (0.35 degree of visual angle) in the corresponding part of macaque primary visual cortex (Hubel and Wiesel, 1974).

Despite the improvement in spatial resolution offered by the advanced fMRI acquisition sequence, our measure of the position tuning width was nonetheless still potentially confounded by the temporal lag between neural responses and fMRI signals due to hemodynamic coupling. This potential confounding factor was taken into consideration during the experiment where the BOLD time series was deconvolved with an empirically derived hemodynamic response function before fitting with a 2D Gaussian characterization of position tuning profile. Across voxels, we did not observe significant correlations between the measure of the position tuning width and the parameters of the hemodynamic response function, suggesting that the measure largely reflected the neural response properties rather than the hemodynamic response properties (Supplemental Experimental Procedures section 4.3). In control experiments, we further tested whether the fMRI signal quality might confound our measure of the position tuning width. We found that the fMRI signal-to-noise ratio was rather homogenous across the cortical surface and did not vary systematically with the measure of the position tuning width (Supplemental Experimental Procedures section 4.4). These control studies suggested that our measure of neural population tuning for visual field position was not confounded by intervoxel variability in fMRI signal properties.

From the fMRI-based measure of neural population tuning, we studied variability within and across participants in the position tuning width. Similar to variability in visual cortical thickness, we observed an intraindividual increase in the position tuning width from parafovea to perifovea (V1 sulci, T = 20.894, p < 0.0001, n = 20; V1 gyri, T = 16.458, p < 0.0001, n = 20; V2 sulci, T = 22.849, p < 0.0001, n = 20; and V2 gyri, T = 20.240, p < 0.0001, n = 20) and from sulci to gyri (V1 parafovea, T = 7.834, p < 0.0001, n = 20; V1 perifovea, T = 5.478, p < 0.0001, n = 20; V2 parafovea, T = 6.675, p < 0.0001, n = 20; and V2 perifovea, T = 6.182, p < 0.0001, n = 20), where the slope of this eccentricity-dependent increase varied across participants over 2-fold. In addition to this eccentricity-dependent variability (Dumoulin and Wandell, 2008), we found that the position tuning width also varied intraindividually across different visual field positions at the same eccentricity and the same cortical folding. Moreover, even for the same visual field position, the position tuning width still exhibited a substantial degree of variability across participants. As such, variability in the position tuning width could be decomposed into an eccentricity-dependent and an eccentricity-independent component, respectively.

### Variability in Perceptual Discrimination Threshold

The measure of perceptual discrimination for visual field position was based on the standard staircase procedure with a two-alternative forced-choice task. In the experiment, the visual field position difference between two horizontally offset stimuli was varied in a 2-up-1-down staircase to assess the minimum position difference that participants could discriminate. To improve the reliability of this perceptual discrimination measure, we conducted two separate experiments, employing respectively, a spatial forced-choice task where participants discriminated the position difference between two concurrently presented stimuli, and a temporal forced-choice paradigm where participants discriminated the position difference between two sequentially presented stimuli. We found that the measure of the position discrimination threshold from these two different experiments was correlated across participants (r = 0.652, p < 0.01, n = 20), suggesting that the measure reflected a robust, trait-like perceptual variability (Supplemental Experimental Procedures section 5.1).

For each participant, the position discrimination threshold was measured at 13 nonoverlapping visual field positions in 13 independent experiments. The 13 visual field positions covered three eccentricities (0, 4.7, and 6.7 degree) and six polar angles (45, 90, 135, 225, 270, and 315 degree). Such a distributed coverage of the visual field allowed a comprehensive characterization of intraindividual perceptual variability. Specifically, by distributing the 13 visual field positions along both the axis of eccentricity and the axis of polar angle, we separately assessed the eccentricity-dependent and the eccentricity-independent component of intraindividual perceptual variability that reflected respectively, how the position discrimination threshold changed along visual field eccentricity and varied across different visual field positions at the same eccentricity (Supplemental Experimental Procedures section 5.2).

From the psychophysical measure of perceptual discrimination, we studied variability within and across participants in the position discrimination threshold. Consistent with previous reports (Duncan and Boynton, 2003), we observed an intraindividual increase in the position discrimination threshold from parafovea to perifovea, where the slope of this eccentricity-dependent increase varied across participants over 2-fold. When the factor of eccentricity was controlled for, we found that the position discrimination threshold still exhibited a substantial degree of variability. In particular, at a fixed visual field eccentricity, the position discrimination threshold varied across different visual field positions for the same participant and across different participants for the same visual field position. Therefore, similar to variability in the position tuning width, variability in the position discrimination threshold could be decomposed into an eccentricity-dependent and an eccentricity-independent component, respectively.

### Dependence of Neural Population Tuning Width and Perceptual Discrimination Threshold on Visual Cortical Anatomy at a Fixed Visual Field Eccentricity

**Figure 3:**
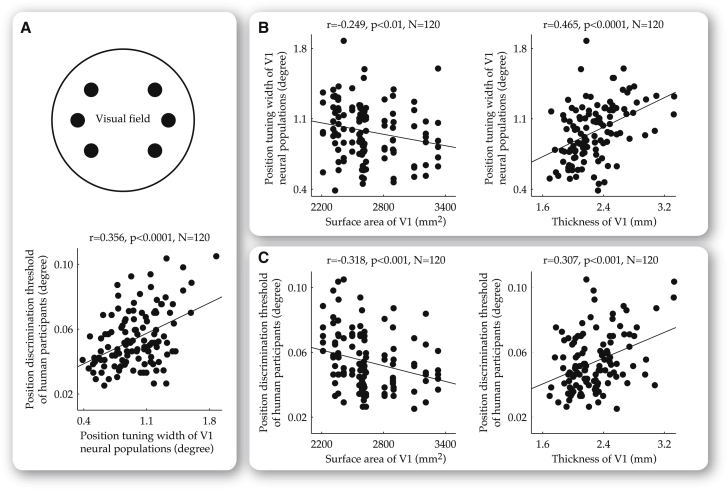
Dependence of Neural Population Tuning Width and Perceptual Discrimination Threshold on V1 Anatomy at a Fixed Visual Field Eccentricity Across a total of 20 participants and six visual field positions at 4.7 degree eccentricity, the position tuning width of V1 neural populations (measured using fMRI) and the position discrimination threshold of human participants (measured using psychophysics) were plotted against each other (A) and against V1 surface area or V1 thickness (B and C). These analyses revealed a positive correlation between the position discrimination threshold of our participants and the position tuning width of V1 neural populations (A), as well as a dependence of both the position tuning width (B) and position discrimination threshold (C) on V1 anatomy. Specifically, the position tuning width of V1 neural populations (B), and the position discrimination threshold of our participants (C), correlated positively with V1 surface area, but negatively with V1 thickness. A further analysis, where interindividual variability and intraindividual variability were regressed out respectively, revealed that these correlations between neural population tuning width, perceptual acuity, and V1 anatomy existed both within and across individuals. Each data point represents the measures at a single visual field position from a single participant. Statistical values reflect permutation-based Spearman’s rank correlation with familywise error (FWE) correction for multiple comparisons.

As variability in the position tuning width and position discrimination threshold consisted of both eccentricity-independent and eccentricity-dependent components, we conducted separate analyses to explore the influences that visual cortical anatomy exerted on these two components, respectively. To control for the factor of eccentricity and study the eccentricity-independent component, we analyzed the relationships between visual cortical anatomy, neural population tuning width, and perceptual acuity at a fixed visual field eccentricity (4.7 degree). Across a total of 20 participants and six visual field positions at 4.7 degree eccentricity, we plotted the position tuning width of V1 neural populations and position discrimination threshold of human participants, first against each other to address the behavioral significance of neural population tuning (Figure 3A) and then against thickness or surface area of V1 to address the functional impacts of visual cortical anatomy (Figures 3B and 3C).

We found that the position tuning width of V1 neural populations correlated positively with the position discrimination threshold of our participants (Figure 3A; r = 0.356, p < 0.0001, n = 120). This correlation reflected a combined contribution of intraindividual and interindividual factors. To address the contribution of each factor, we conducted separate analysis where we calculated, for each participant (n = 20), the position discrimination threshold as well as the position tuning width averaged across the six visual field positions, and for each visual field position (n = 6), the position discrimination threshold as well as the position tuning width averaged across the 20 participants. By subtracting the averages of individual participants, we factored out interindividual variability and studied the contribution of intraindividual factors. Similarly, by subtracting the averages of individual visual field positions, we factored out intraindividual variability and addressed the contribution of interindividual factors. In both cases, we still observed a positive correlation between the position tuning width of V1 neural populations and position discrimination threshold of our participants (intraindividually, r = 0.350, p < 0.0001, n = 120; interindividually, r = 0.359, p < 0.0001, n = 120). This observation illustrated a close correspondence between neural population tuning and human perceptual discrimination.

In addition to the correlation with perceptual discrimination, the position tuning width of V1 neural populations also exhibited correlations with V1 anatomy. Specifically, neural populations in V1 with a larger surface area tended to have a smaller position tuning width (Figure 3B; r = −0.249, p < 0.01, n = 120). In contrast to this negative correlation with V1 surface area, the position tuning width of V1 neural populations exhibited a positive correlation with V1 thickness, where neural populations at V1 locations with a greater thickness tended to have a larger position tuning width (Figure 3B; r = 0.465, p < 0.0001, n = 120). This correlation between the position tuning width and V1 thickness was observed both within individuals (r = 0.394, p < 0.0001, n = 120, interindividual variability factored out), and across individuals (r = 0.423, p < 0.0001, n = 120, intraindividual variability factored out). These findings suggested that the two anatomical dimensions, thickness and surface area, of V1 had opposite impacts on neural population tuning for visual field position.

The functional impacts of visual cortical anatomy on neural population tuning were further reflected in perceptual discrimination. We found that thickness and surface area of V1 both exhibited correlations with the position discrimination threshold of our participants. Specifically, participants with a larger V1 surface area tended to have a smaller position discrimination threshold (Figure 3C; r = −0.318, p < 0.001, n = 120). In contrast to this negative correlation between the position discrimination threshold and V1 surface area, a positive correlation was observed between the position discrimination threshold at different visual field positions and V1 thickness at corresponding cortical locations (Figure 3C; r = 0.307, p < 0.001, n = 120), within individuals (r = 0.339, p < 0.001, n = 120, interindividual variability factored out) as well as across individuals (r = 0.311, p < 0.001, n = 120, intraindividual variability factored out).

**Figure 4:**
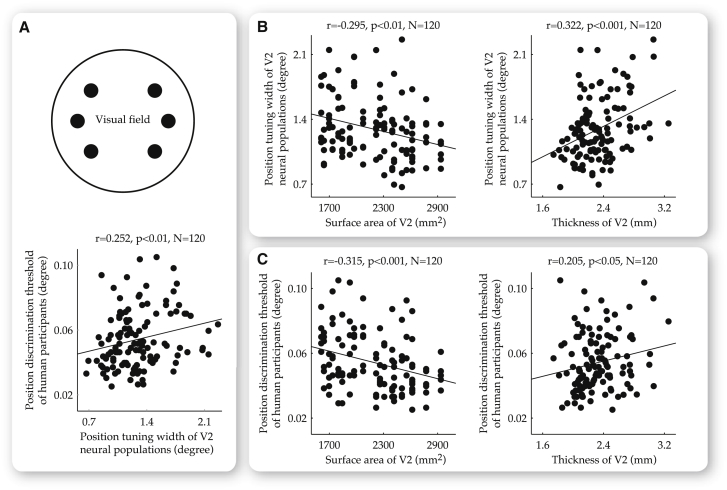
Dependence of Neural Population Tuning Width and Perceptual Discrimination Threshold on V2 Anatomy at a Fixed Visual Field Eccentricity Across a total of 20 participants and six visual field positions at 4.7 degree eccentricity, the position tuning width of V2 neural populations (measured using fMRI) and the position discrimination threshold of human participants (measured using psychophysics) were plotted against each other (A) and against V2 surface area or V2 thickness (B and C). These analyses revealed a positive correlation between the position discrimination threshold of our participants and the position tuning width of V2 neural populations (A), as well as a dependence of both the position tuning width (B), and position discrimination threshold (C) on V2 anatomy. Specifically, the position tuning width of V2 neural populations (B), and the position discrimination threshold of our participants (C), correlated positively with V2 surface area, but negatively with V2 thickness. A further analysis, where interindividual variability and intraindividual variability were regressed out respectively, revealed that these correlations between neural population tuning width, perceptual acuity, and V2 anatomy existed both within and across individuals. Each data point represents the measures at a single visual field position from a single participant. Statistical values reflect permutation-based Spearman’s rank correlation with FWE correction for multiple comparisons.

These observations revealed that thickness and surface area of human V1 had influences on both neural population tuning and perceptual discrimination. To explore the influences that V2 anatomy might exert on the position tuning width of V2 neural populations and the position discrimination threshold of our participants, we applied a similar analysis. We plotted the position tuning width of V2 neural populations and the position discrimination threshold of our participants, first against each other and then against thickness or surface area of V2, for a total of 20 participants and six visual field positions at 4.7 degree eccentricity. Similar to our observations in V1, the position tuning width of V2 neural populations correlated positively with the position discrimination threshold of our participants (Figure 4A; r = 0.252, p < 0.01, n = 120; intraindividually, r = 0.230, p < 0.05, n = 120; and interindividually, r = 0.261, p < 0.01, n = 120). Moreover, both the position tuning width and position discrimination threshold exhibited correlations with V2 anatomy. Specifically, participants with a larger V2 surface area tended to have a smaller position tuning width (Figure 4B; r = −0.295, p < 0.01, n = 120) and a smaller position discrimination threshold (Figure 4C; r = −0.315, p < 0.001, n = 120). In contrast, a larger position tuning width (Figure 4B; r = 0.322, p < 0.001, n = 120; intraindividually, r = 0.366, p < 0.0001, n = 120; and interindividually, r = 0.276, p < 0.01, n = 120) and a larger position discrimination threshold (Figure 4C; r = 0.205, p < 0.05, n = 120; intraindividually, r = 0.200, p < 0.05, n = 120; and interindividually, r = 0.193, p < 0.05, n = 120) were observed at the visual field positions that corresponded to V2 locations with a larger thickness.

### Dependence of Neural Population Tuning Width and Perceptual Discrimination Threshold on Visual Cortical Anatomy along Visual Field Eccentricity

Our analyses at a fixed visual field eccentricity (4.7 degree) suggested that thickness and surface area of human early visual cortices (V1 and V2) had opposite impacts on neural population tuning that in turn affected perceptual discrimination. To study whether these observations were specific to certain eccentricity or were generalizable across the visual field, we conducted further analyses to address the impacts that visual cortical anatomy had on eccentricity-dependent variability in neural population tuning width and perceptual discrimination threshold. Specifically, by fitting the position tuning width and position discrimination threshold as a function of visual field eccentricity, we explored how visual cortical anatomy might relate to the slope and the intercept of the fit. A relationship with the slope of the fit would indicate that the position tuning width and position discrimination threshold got more dependent on visual cortical anatomy as one approached the peripheral visual field, whereas a relationship with the intercept of the fit would indicate an increased dependence toward the central visual field.

**Figure 5:**
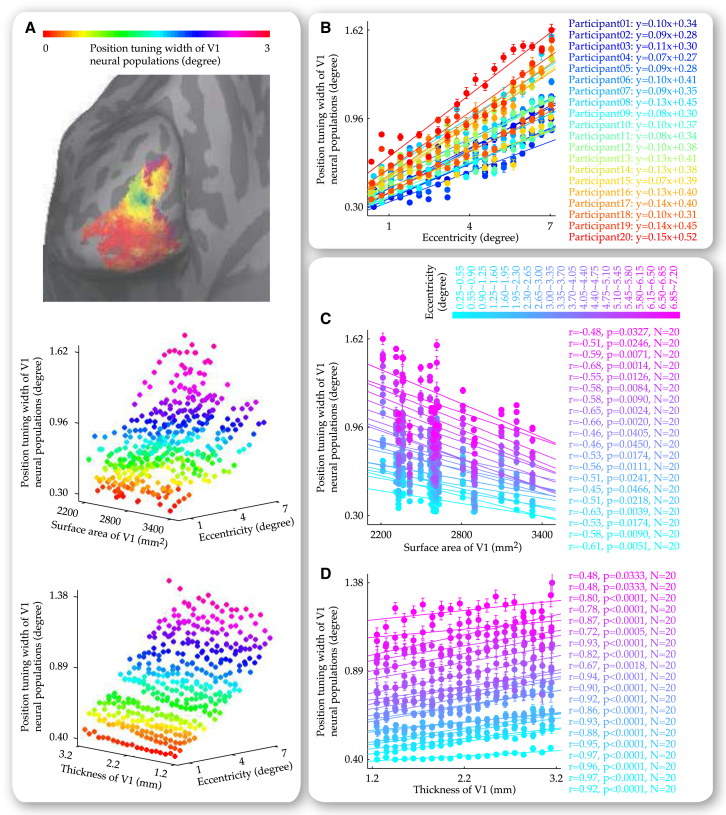
Relationship between Neural Population Tuning Width and V1 Anatomy along Visual Field Eccentricity The cortical surface map from a representative participant illustrated the width of neural population tuning at individual V1 cortical surface locations (vertices) for corresponding visual field positions (A). Based on the cortical surface maps from all 20 participants, we plotted the position tuning width at individual V1 locations against visual field eccentricities these locations responded to and V1 anatomy at these locations. The 3D plots were binned into data grids where individual data points represented the position tuning width averaged over V1 locations that responded to similar eccentricities and were from the same participant or had similar thickness (A). The data grids allowed us to disentangle the influences that visual field eccentricity (B) and V1 anatomy (C and D) exerted on the position tuning width of V1 neural populations. Specifically, along the axis of V1 surface area, each plot of the position tuning width, visual field eccentricity represented the data from a single participant and illustrated the increase in the position tuning width with visual field eccentricity (B). Along the axis of visual field eccentricity, each plot of the position tuning width, V1 anatomy represented the data from a single eccentricity range and illustrated the dependence of the position tuning width on V1 surface area (C) or V1 thickness (D). Data points are color coded according to the position tuning width (A), the participant (B), or the visual field eccentricity (C and D). Equations (B) reflect linear fit to the plot of the position tuning width, visual field eccentricity. Statistical values (C and D) reflected permutation-based Spearman’s rank correlation with FWE correction for multiple comparisons. Error bars represent 1 SEM.

Across the group of 20 participants and the retinotopically delineated coverage of the visual field (0.25–7.2 degree eccentricity), we plotted the position tuning width at individual V1 locations (vertices) against visual field eccentricities these locations responded to and V1 surface area of the participants. The data were binned into a data grid where individual data points represented the position tuning width averaged over V1 locations (vertices) that were from the same participant and responded to similar eccentricities (Figure 5A). This 3D data grid allowed us to separately address the influences that visual field eccentricity and V1 surface area exerted on the position tuning width of V1 neural populations. Along the axis of V1 surface area, we fitted individual plots of the position tuning width, visual field eccentricity with linear regression functions, where each plot represented the data from a single participant (Figure 5B). We found that the slope (r = −0.549, p < 0.05, n = 20) and the intercept (r = −0.614, p < 0.01, n = 20) of the linear fit both correlated negatively with surface area of V1, while the goodness of the fit did not exhibit such correlation (r = 0.272, p = 0.245, n = 20). Therefore, neural populations in V1 with a larger surface area tended to have a smaller position tuning width near fovea, as well as a slower position tuning width increase along visual field eccentricity. These results suggested that the dependence of the position tuning width on V1 surface area was likely to be a general observation that spanned the visual field. Indeed, when correlation analysis was applied along the axis of visual field eccentricity directly on individual plots of the position tuning width, V1 surface area (Figure 5C), we observed negative correlations within individual ranges of visual field eccentricity between the position tuning width and V1 surface area.

Thus, the functional impacts of V1 surface area on neural population tuning width, which we observed at 4.7 degree eccentricity, were generalizable across the visual field. To study the functional impacts of V1 thickness, we applied a similar analytic approach. Across the group of 20 participants and the retinotopically delineated coverage of visual field (0.25−7.2 degree eccentricity), we plotted the position tuning width at individual V1 locations (vertices) against visual field eccentricities that these locations responded to and V1 thickness at these locations. The data were binned into a data grid where individual data points represented the position tuning width averaged over V1 locations (vertices) that were similar in thickness and responded to similar eccentricities (Figure 5A). Through this 3D data grid, we separately addressed the influences that visual field eccentricity and V1 thickness exerted on the position tuning width of V1 neural populations. Along the axis of V1 thickness, we fitted individual plots of the position tuning width, visual field eccentricity with linear regression functions, where each plot represented the data from a single thickness range of 0.1 mm. We found that the slope (r = 0.528, p < 0.05, n = 20) and the intercept (r = 0.935, p < 0.0001, n = 20) of the linear fit both correlated positively with thickness of V1, while the goodness of the fit did not covary with V1 thickness (r = −0.147, p = 0.534, n = 20). The influences of V1 thickness on both the slope and the intercept of the fit suggested that the dependence of position tuning width on V1 thickness was not specific to certain eccentricity ranges, but was instead generalizable across the visual field. To verify this, we applied correlation analysis directly to individual plots of the position tuning width, V1 thickness, along the axis of visual field eccentricity (Figure 5D). We observed positive correlations between the position tuning width and V1 thickness, within individual ranges of visual field eccentricity.

These analyses suggested that, across the visual field, neural population tuning in V1 for a specific visual field position was affected jointly by thickness at corresponding V1 locations and surface area of V1. We further investigated whether such influences on neural population tuning would have behavioral consequences on perceptual discrimination. The threshold of perceptual discrimination, measured at 13 nonoverlapping visual field positions covering three eccentricities (0, 4.7, and 6.7 degree) and six polar angles (45, 90, 135, 225, 270, and 315 degree), was projected onto V1 to generate a cortical map of perceptual acuity. This cortical projection allowed us to relate perceptual acuity at different visual field positions with V1 anatomy at corresponding cortical locations, using analyses similar to the ones on neural population tuning width.

**Figure 6:**
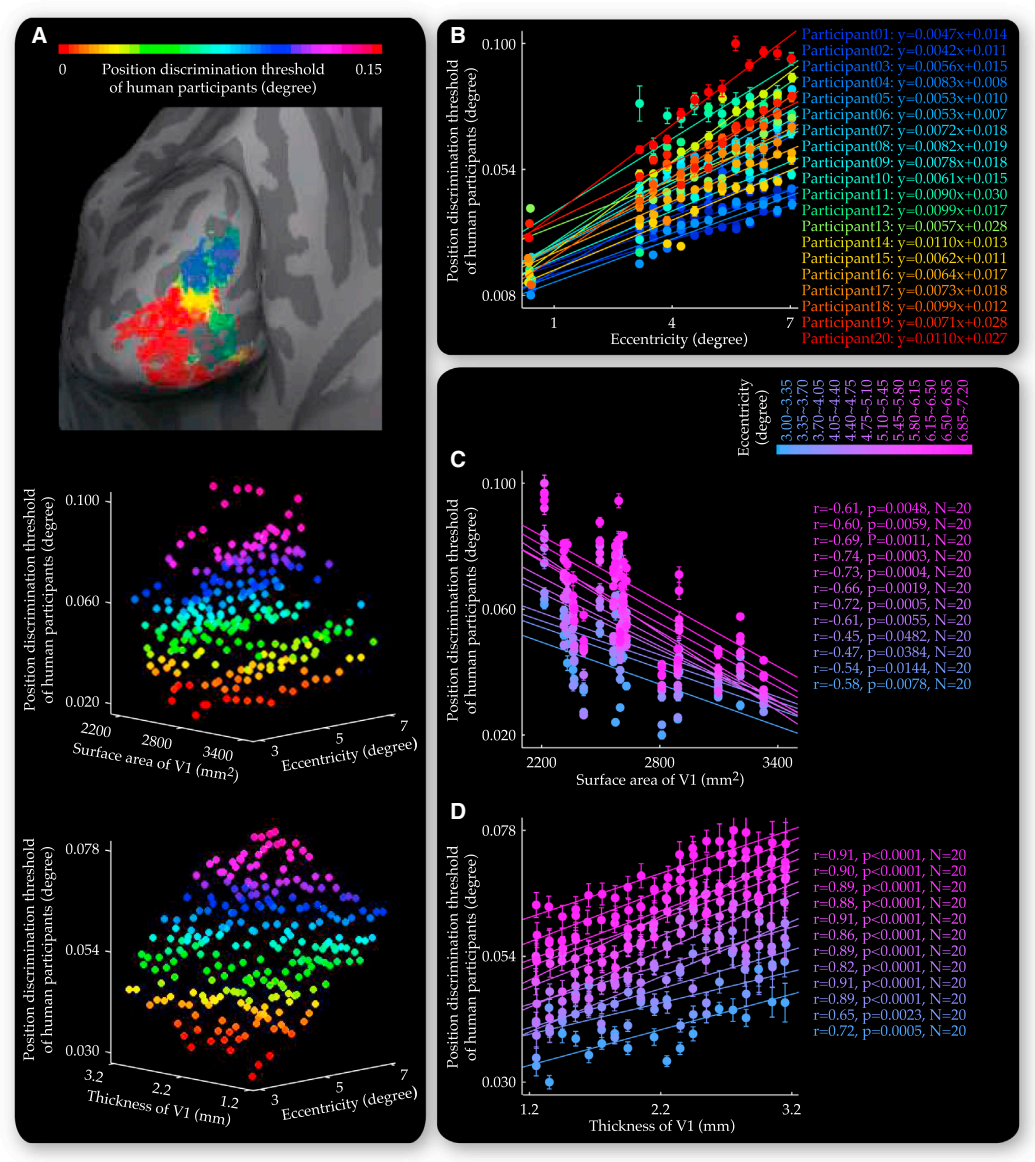
Relationship between Perceptual Discrimination Threshold and V1 Anatomy along Visual Field Eccentricity The threshold of perceptual discrimination, measured at 13 nonoverlapping visual field positions covering three eccentricities (0, 4.7, and 6.7 degree) and six polar angles (45, 90, 135, 225, 270, and 315 degree), was projected onto V1 to generate a cortical surface map for each participant that illustrated variability across different V1 cortical surface locations (vertices) in perceptual discrimination threshold for corresponding visual field positions (A). Based on the cortical surface maps from all 20 participants, we plotted the position discrimination threshold at individual V1 locations against visual field eccentricities these locations responded to and V1 anatomy at these locations. The 3D plots were binned into data grids where individual data points represented the position discrimination threshold averaged over V1 locations that responded to similar eccentricities and were from the same participant or had similar thickness (A). For each participant, different V1 locations that were projected with the measure of position discrimination threshold at the central visual field (zero eccentricity) were binned into a single data point. The data grids allowed us to disentangle the influences that visual field eccentricity (B) and V1 anatomy (C and D) exerted on the position discrimination threshold. Specifically, along the axis of V1 surface area, each plot of the position discrimination threshold, visual field eccentricity represented the data from a single participant and illustrated the increase in the position discrimination threshold with visual field eccentricity (B). Along the axis of visual field eccentricity, each plot of the position discrimination threshold, V1 anatomy represented the data from a single eccentricity range and illustrated the dependence of the position discrimination threshold on V1 surface area (C) or V1 thickness (D). Data points are color coded according to the position discrimination threshold (A), the participant (B), or the visual field eccentricity (C and D). Equations (B) reflect linear fit to the plot of the position discrimination threshold, visual field eccentricity. Statistical values (C and D) reflected permutation-based Spearman’s rank correlation with FWE correction for multiple comparisons. Error bars represent 1 SEM.

On a vertex-by-vertex level, we plotted the position discrimination threshold at individual V1 locations (vertices) against visual field eccentricities that these locations responded to and V1 thickness at these locations or V1 surface area of the participants (Figure 6A). The data were binned into data grids characterizing the increase in the position discrimination threshold along visual field eccentricity, as well as the relationships between the position discrimination threshold and V1 anatomy within individual ranges of visual field eccentricity. Mirroring the observations on neural population tuning width, we found that the functional impacts of V1 surface area and V1 thickness on perceptual acuity, which we observed at 4.7 degree eccentricity, were generalizable across the visual field. Specifically, the position discrimination threshold correlated negatively with V1 surface area (Figure 6C), in a fashion that participants with a larger V1 surface area had not only a smaller position discrimination threshold in the fovea (−r = 0.675, p < 0.01, n = 20), but also a slower position discrimination threshold increase along visual field eccentricity (−r = 0.532, p < 0.05, n = 20). In contrast, positive correlations were observed within individual ranges of visual field eccentricity between the position discrimination threshold and V1 thickness (Figure 6D).

**Figure 7:**
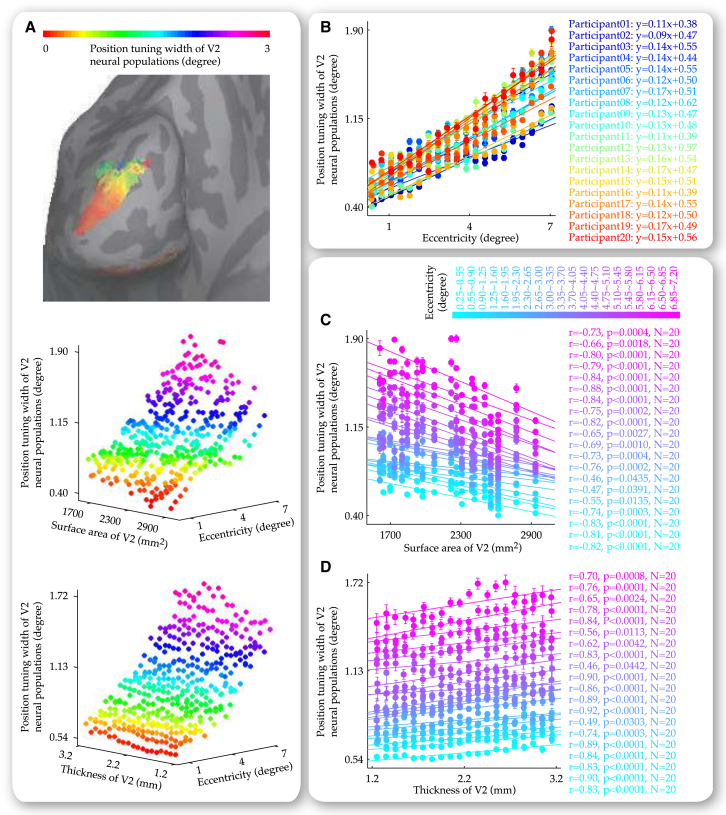
Relationship between Neural Population Tuning Width and V2 Anatomy along Visual Field Eccentricity The cortical surface map from a representative participant illustrated the width of neural population tuning at individual V2 cortical surface locations (vertices) for corresponding visual field positions (A). Based on the cortical surface maps from all 20 participants, we plotted the position tuning width at individual V2 locations against visual field eccentricities these locations responded to and V2 anatomy at these locations. The 3D plots were binned into data grids where individual data points represented the position tuning width averaged over V2 locations that responded to similar eccentricities and were from the same participant or had similar thickness (A). The data grids allowed us to disentangle the influences that visual field eccentricity (B) and V2 anatomy (C and D) exerted on the position tuning width of V2 neural populations. Specifically, along the axis of V2 surface area, each plot of the position tuning width, visual field eccentricity represented the data from a single participant and illustrated the increase in the position tuning width with visual field eccentricity (B). Along the axis of visual field eccentricity, each plot of the position tuning width, V2 anatomy represented the data from a single eccentricity range and illustrated the dependence of the position tuning width on V2 surface area (C) or V2 thickness (D). Data points are color coded according to the position tuning width (A), the participant (B), or the visual field eccentricity (C and D). Equations (B) reflect linear fit to the plot of the position tuning width, visual field eccentricity. Statistical values (C and D) reflected permutation-based Spearman’s rank correlation with FWE correction for multiple comparisons. Error bars represent 1 SEM.

**Figure 8:**
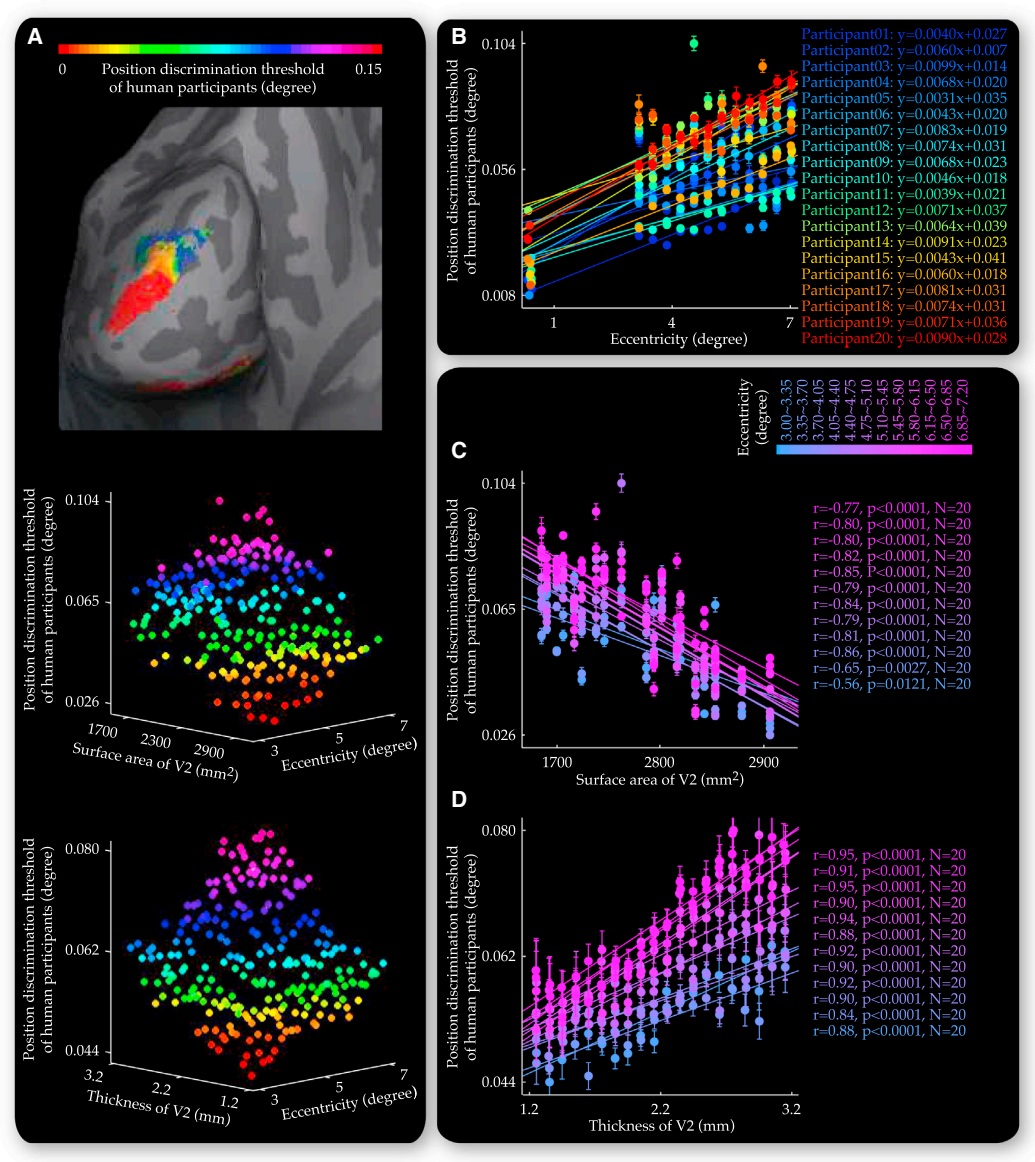
Relationship between Perceptual Discrimination Threshold and V2 Anatomy along Visual Field Eccentricity The threshold of perceptual discrimination, measured at 13 nonoverlapping visual field positions covering three eccentricities (0, 4.7, and 6.7 degree) and six polar angles (45, 90, 135, 225, 270, and 315 degree), was projected onto V2 to generate a cortical surface map for each participant that illustrated variability across different V2 cortical surface locations (vertices) in perceptual discrimination threshold for corresponding visual field positions (A). Based on the cortical surface maps from all 20 participants, we plotted the position discrimination threshold at individual V2 locations against visual field eccentricities these locations responded to and V2 anatomy at these locations. The 3D plots were binned into data grids where individual data points represented the position discrimination threshold averaged over V2 locations that responded to similar eccentricities and were from the same participant or had similar thickness (A). For each participant, different V2 locations that were projected with the measure of the position discrimination threshold at the central visual field (zero eccentricity) were binned into a single data point. The data grids allowed us to disentangle the influences that visual field eccentricity (B) and V2 anatomy (C and D) exerted on the position discrimination threshold. Specifically, along the axis of V2 surface area, each plot of the position discrimination threshold, visual field eccentricity represented the data from a single participant and illustrated the increase in the position discrimination threshold with visual field eccentricity (B). Along the axis of visual field eccentricity, each plot of the position discrimination threshold, V2 anatomy represented the data from a single eccentricity range and illustrated the dependence of the position discrimination threshold on V2 surface area (C) or V2 thickness (D). Data points are color coded according to the position discrimination threshold (A), the participant (B), or the visual field eccentricity (C and D). Equations (B) reflect linear fit to the plot of the position discrimination threshold, visual field eccentricity. Statistical values (C and D) reflected permutation-based Spearman’s rank correlation with FWE correction for multiple comparisons. Error bars represent 1 SEM.

Together, these analyses suggested that our observations at 4.7 degree eccentricity, where thickness and surface area of V1 exerted opposite influences on the position tuning width of V1 neural populations with behavioral consequences on the position discrimination threshold of human participants, were generalizable across the visual field. To investigate whether such generalization was also observable in V2, we plotted the position tuning width (Figure 7) and position discrimination threshold (Figure 8) at individual V2 locations (vertices) against visual field eccentricities these locations responded to and V2 anatomy at these locations. Similar to our observations in V1, surface area of V2 correlated negatively with the position tuning width of V2 neural populations (Figure 7C) and the position discrimination threshold of our participants (Figure 8C), and specifically, with their value near the fovea (tuning width, r = −0.729, p < 0.001, n = 20 and discrimination threshold, r = −0.596, p < 0.01, n = 20), as well as their slope of increase along visual field eccentricity (tuning width, r = −0.705, p < 0.001, n = 20 and discrimination threshold, r = −0.642, p < 0.01, n = 20). In contrast, thickness of V2 exhibited positive correlations with the position tuning width (Figure 7D) and position discrimination threshold (Figure 8D), within individual ranges of visual field eccentricity.

## Discussion

It is intuitive to assume that a larger cortical volume has some behavioral advantage. Indeed, within species, a positive correlation is usually observed between performance on behavioral tasks and local cortical volume in task-relevant cortical regions (Kanai and Rees, 2011). However, the fundamental questions of whether a larger cortical volume is indeed the critical factor and why cortical volume is even relevant to understanding behavioral performance remain unaddressed. Here, we suggest two possible mechanisms. It is plausible that the influences of cortical anatomy on behavioral performance are mediated simply by the volume of cortical tissue available for information processing and, correspondingly, the signal-to-noise ratio during information processing. Alternatively, the neural response properties that are associated with cortical anatomy may underlie its influences on behavioral performance. To disentangle these two hypotheses, we separately studied the two anatomical dimensions, thickness and surface area, of cerebral cortex. These two elementary dimensions both contribute to cortical volume, yet characterize distinct aspects of volumetric changes (local versus global) that may differently affect the neural response properties. This allowed us to address whether it is cortical volumes per se or the associated neural response properties that link cortical anatomy to behavioral performance.

We used early visual cortices as a model system, since the orderly representation of visual field position in early visual cortices allowed fMRI-based, noninvasive characterization of neural population tuning (Dumoulin and Wandell, 2008; Fischer and Whitney, 2009). Utilizing this fMRI-based measure of neural population tuning, we investigated how the anatomy of human early visual cortices influenced the population tuning properties of visual cortical neurons and whether such influences were behaviorally significant. We found that neural population tuning and perceptual discrimination were finer in individuals with a larger surface area of early visual cortices. Therefore, a larger visual cortical volume, if it came from a larger visual cortical surface area, was associated with a better performance in perceptual discrimination and a higher selectivity in neural population tuning. Intriguingly, the exact opposite impacts were observed for visual cortical thickness, where neural population tuning and perceptual discrimination were finer for visual field positions corresponding to thinner parts of early visual cortices. As such, a larger visual cortical volume, if it came from a larger visual cortical thickness, was associated with a poorer performance in perceptual discrimination and a lower selectivity in neural population tuning.

Our findings suggested a larger visual cortical volume is not in itself advantageous for visual perception. Instead, a perceptually advantageous cortical design may involve a thinned visual cortex with an enlarged surface area. This is consistent with the developmental trend that sensory experience drives the expansion of sensory cortical maps, but thinning of sensory cortex (Gilbert et al., 2001; Jiang et al., 2009). Moreover, the association between a thinner visual cortex and a finer visual function is consistent with a similar trend in the retina. In the retina, the part with the highest acuity, the fovea, is also the thinnest. The fovea has only one photoreceptor layer that potentially minimizes the absorption of light signal along the retinal pathway (Jacobson et al., 2007). As such, a finer visual function may in general be achieved not through a simple increase in tissue volume, but instead through the optimization of tissue distribution. Indeed, a thinned visual cortex with an enlarged surface area is likely to optimize the selectivity of visual cortical neurons by maximizing the number of intercolumnar processing units and minimizing the delay of interlaminar processing (Bugbee and Goldman-Rakic, 1983; Rakic, 1988; Mountcastle, 1997; Jones, 2000; Kaas, 2000).

Specifically, thickening of visual cortex is likely to burden intracortical processing, as the axons and the dendrites of interlaminar connections would need to double and quadruple in diameter to improve interlaminar conduction speed in order to maintain the same interlaminar processing time (delay) (Kaas, 2000). Due to the physical constrains on wiring costs, interlaminar connections tend to fall behind the increase in cortical thickness, leading to increased interlaminar processing time (Ringo, 1991; Kaas, 2000; Angelucci et al., 2002; Sporns and Zwi, 2004; Lewis et al., 2009). Such an increase in interlaminar processing time would facilitate response synchronization among different cortical columns, and in turn, decrease the functional specificity (selectivity) of individual cortical columns (Koch, 1984; Ringo, 1991; Kaas, 2000; Womelsdorf et al., 2007; Sun and Dan, 2009). Therefore, a larger visual cortical thickness is likely to be associated with a lower selectivity in neural tuning, and correspondingly, a poorer performance in perceptual discrimination. By contrast, the enlargement of visual cortical surface area is likely to benefit intracortical processing through an increase in the number of cortical columns available for intercolumnar processing. This increased number of cortical columns, at the same time, would be accompanied by a proportionally decreased connectivity between different cortical columns, as the absolute length of intercolumnar connections is physically constrained and remains independent of visual cortical size (Bugbee and Goldman-Rakic, 1983; Rakic, 1988; Ringo, 1991; Mountcastle, 1997; Jones, 2000; Kaas, 2000). Such a decrease in the proportion of cortical columns with which an individual cortical column connects would in turn increase the functional specificity (selectivity) of individual cortical columns. Therefore, a larger visual cortical surface area is likely to be associated with a higher selectivity in neural tuning, and correspondingly, a better performance in perceptual discrimination.

Limited by the current resolution of noninvasive neuroimaging techniques, an empirical assessment of intracortical processing in human participants is not easy. Nevertheless, it might be of interest for future studies to explore whether a visual cortical model that incorporated these hypothetical changes in intracortical processing could reproduce our empirically observed correlations between visual cortical anatomy and neural population tuning. Regardless of the underlying mechanisms, our findings revealed that the population tuning properties of visual cortical neurons play an important role in linking visual cortical anatomy to visual perception. We showed that it is not cortical volume per se, but rather the associated neural response properties that mediate the influences of cortical anatomy on behavioral performance. This raises concerns for the classical approach taken in studying the anatomical basis of behavioral performance, where one simply searches for cortical regions whose local volume correlates positively with behavioral performance (Kanai and Rees, 2011). By demonstrating that the two determinants of cortical volume, cortical thickness and cortical surface area, may have opposite functional impacts (at least for visual perception), our findings call for a more nuanced approach to be taken in future research, where the effects of variability in cortical thickness and cortical surface area are examined independently, and any negative correlation between cortical volume and behavioral performance is not overlooked. Moreover, by showing (albeit implicitly) that a thinned visual cortex with an enlarged surface area is perceptually advantageous, our findings suggested a future research direction where one may explicitly study what constitutes a behaviorally advantageous cortical design.

## Experimental Procedures

In a group of 20 healthy human adults, we studied the relationships among the anatomy of early visual cortices (V1 and V2), the width of neural population tuning, and the threshold of perceptual discrimination, measured respectively using structural imaging, fMRI, and visual psychophysics. First, we acquired the measure of visual cortical anatomy by applying the surface-based analysis to early visual cortices delineated on the structural imaging data. To improve the reliability of the measure, we used different experimental paradigms, where we delineated early visual cortices retinotopically according to the phase-encoded map (Sereno et al., 1995), retinotopically according to the population-receptive-field map (Dumoulin and Wandell, 2008), or morphologically according to the cortical folding patterns (Desikan et al., 2006), acquired the structural data from in vivo T1-weighted MRI imaging, in vivo quantitative-T1 MRI imaging, or in vitro histology sectioning, and conducted the surface-based analysis in SPM (Ashburner, 2012), FSL (Jenkinson et al., 2012), Freesurfer (Fischl, 2012), or MPAV CBS (Bazin et al., 2013). Then, we measured neural population tuning for visual field position using the method of population-receptive-field mapping (Dumoulin and Wandell, 2008), where a bar stimulus was presented at 64 different visual field positions, and the fMRI BOLD time series recorded from each voxel in early visual cortices was fitted with a 2D Gaussian function quantifying the position tuning width and position tuning peak. We took into consideration the potential confounding factor of fMRI signal properties by conducting control experiments addressing the influences of fMRI spatial sampling, fMRI hemodynamic coupling, and fMRI signal-to-noise ratio on the measure of neural population tuning. Finally, we assessed the threshold for perceptual discrimination of visual field position, based on the psychophysical staircase procedure with a forced-choice task. To test whether the measure represented a perceptual trait robust against the experimental paradigm, we performed separate experiments employing respectively, a spatial forced-choice task where participants discriminated the visual field position difference between two concurrently presented stimuli, and a temporal forced-choice paradigm where participants discriminated the visual field position difference between two sequentially presented stimuli. For each participant, the threshold of perceptual discrimination, measured at 13 nonoverlapping visual field positions covering three eccentricities (0, 4.7, and 6.7 degree) and six polar angles (45, 90, 135, 225, 270, and 315 degree), was projected onto early visual cortices to generate a personalized cortical map of perceptual acuity. Together these measures allowed us to relate, on a voxel basis, the anatomy at different visual cortical locations with the width of neural population tuning and the threshold of perceptual discrimination for corresponding visual field positions. The experiment details are described in Supplemental Experimental Procedures.

## Supplemental Information

Document S1. Supplemental Experimental Procedures and Figures S1–S5

Document S2. Article plus Supplemental Information
