# Supplementary material for: Neural Population Tuning Links Visual Cortical Anatomy to Human Visual Perception
Source: Neuron. 2015 Feb 4;85(3):641–56. doi: 10.1016/j.neuron.2014.12.041 (PMC4321887; doi:10.1016/j.neuron.2014.12.041)
Supplement: Document S1. Supplemental Experimental Procedures and Figures S1–S5 [file mmc1.pdf]

Neuron

Supplemental Information

Neural Population Tuning Links Visual Cortical Anatomy to Human Visual Perception

Chen Song, Dietrich Samuel Schwarzkopf, Ryota Kanai, and Geraint Rees

Supplemental Data

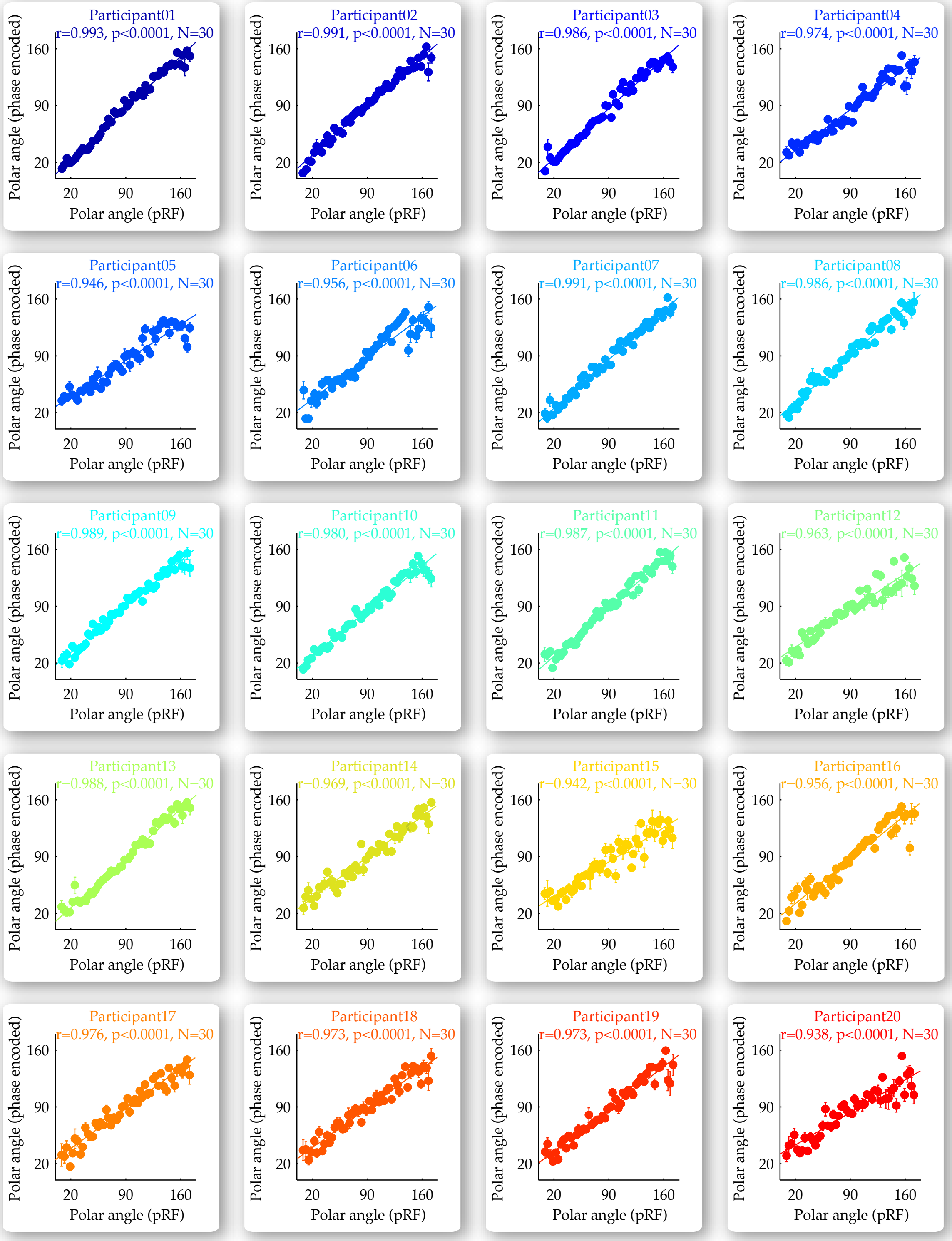

Figure S1. Comparison between Different Retinotopic Mapping Paradigms (Related to Experimental Procedures). Two different retinotopic mapping paradigms, the phase-encoded paradigm and the population-receptive-field paradigm, were used to delineate V1 for each of the twenty participants. The polar angle measures from the two different retinotopic mapping paradigms were plotted against each other on a voxel basis, where voxels responsive to similar polar angle were binned to generate 30 data points for each participant. Based on the voxel-level plot, we calculated the correlation in polar angle measures between the two different retinotopic mapping paradigms. Data points are color coded according to the participant. Statistical values reflect permutation-based Spearman's rank correlation with FWE correction for multiple comparisons.

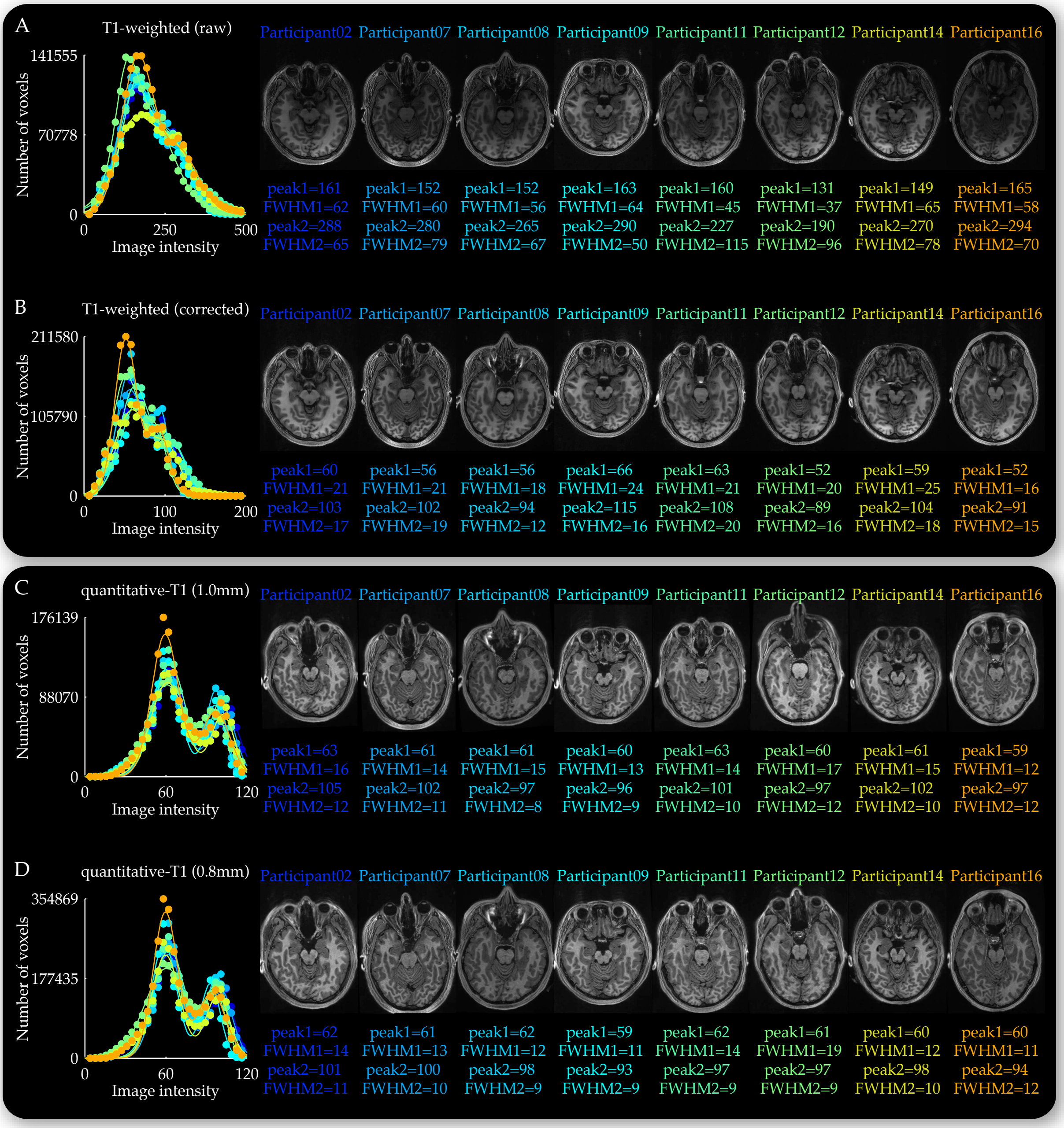

Figure S2. Comparison between T1-weighted MRI Images and Quantitative-T1 MRI Images (Related to Experimental Procedures). Two different MRI sequences, the standard T1-weighted sequence and an advanced quantitative-T1 sequence, were used to collect the structural MRI images for eight of the twenty participants. The distribution of image intensity was plotted on a voxel basis, where voxels with similar intensity value were binned to generate 30 data points for each participant. The double Gaussian fit to the image intensity distribution revealed mixed peaks of white matter and gray matter in the T1-weighted MRI images, not only before (A) but also after (B) non-uniform intensity correction. By contrast, the quantitative-T1 MRI images showed clearly separated peaks of white matter and gray matter with consistent intensity values between 1 mm resolution (C) and 0.8 mm resolution (D). These improvements in intensity homogeneity and tissue contrast from the T1-weighted MRI images to the quantitative-T1 MRI images were further illustrated in the sample cortical slices. Data points are color coded according to the participant. Parameters are derived from the double Gaussian fit to the image intensity distribution.

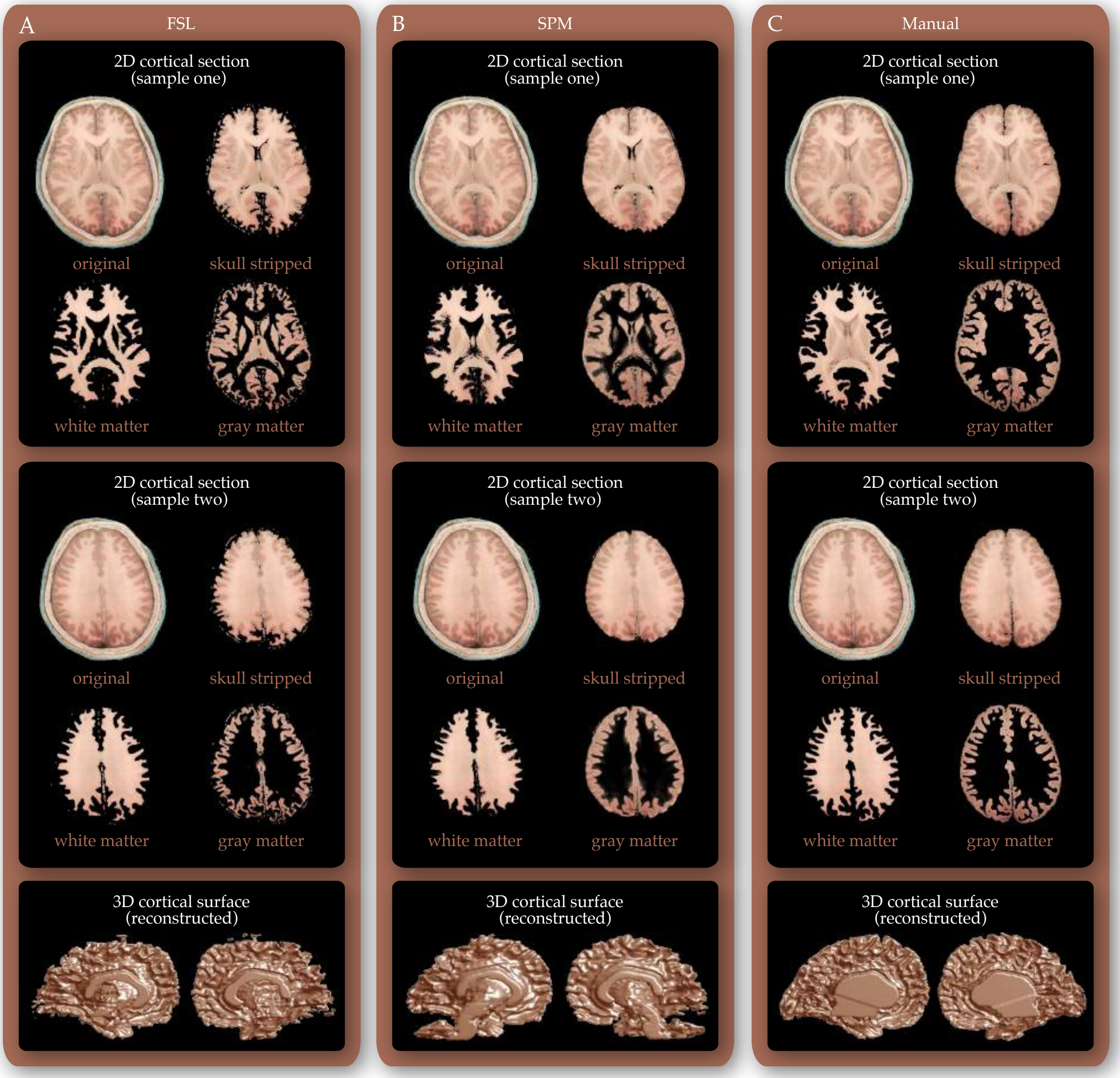

Figure S3. Tissue Segmentation of Histology Data (Related to Experimental Procedures). To build the three-dimensional cortical surface models from the two-dimensional histology images, we segmented each histology image into the white and the gray matter. Our approaches of automatic tissue segmentation, using existing software such as FSL (A) and SPM (B) or using custom-written code, all failed to reach satisfactory accuracy. Consequently, we took the approach of manual tissue segmentation (C). The differences between automatic and manual approaches in the performances of skull stripping, tissue segmentation, and cortical surface reconstruction, were illustrated in the two-dimensional cortical section images and the three-dimensional cortical surface images.

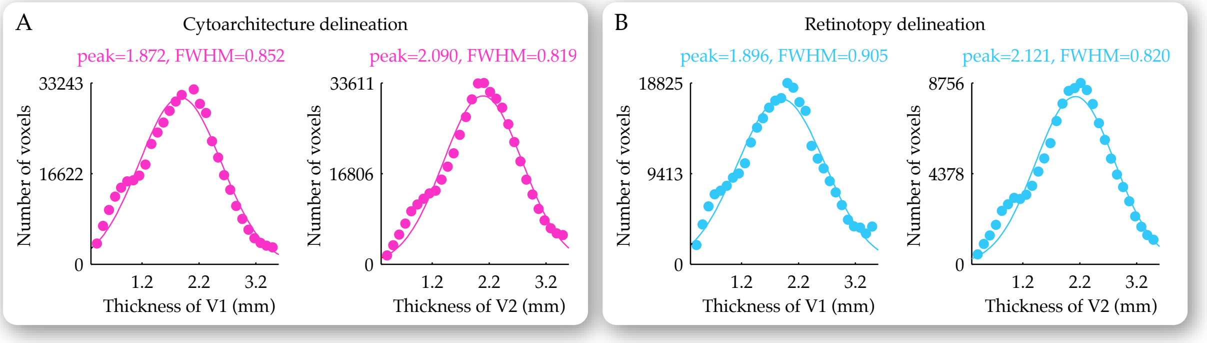

Figure S4. Surface-based Thickness Measure of Histology data (Related to Experimental Procedures). The histology-based measure of visual cortical thickness was acquired for early visual cortices (V1, V2) delineated according to a cytoarchitectonic atlas (A) and according to the average retinotopic map from our fMRI experiments (B), respectively. Based on the delineation of early visual cortices, the cortical thickness at individual visual cortical locations in the histology data was measured as the distance between the white and the pial cortical surfaces. The distribution of histologically-measured visual cortical thickness revealed a substantial degree of intra-individual variability that resembled the observations in MRI-based measure. Data points are color coded according to the delineation method. Parameters are derived from the Gaussian fit to the thickness distribution.

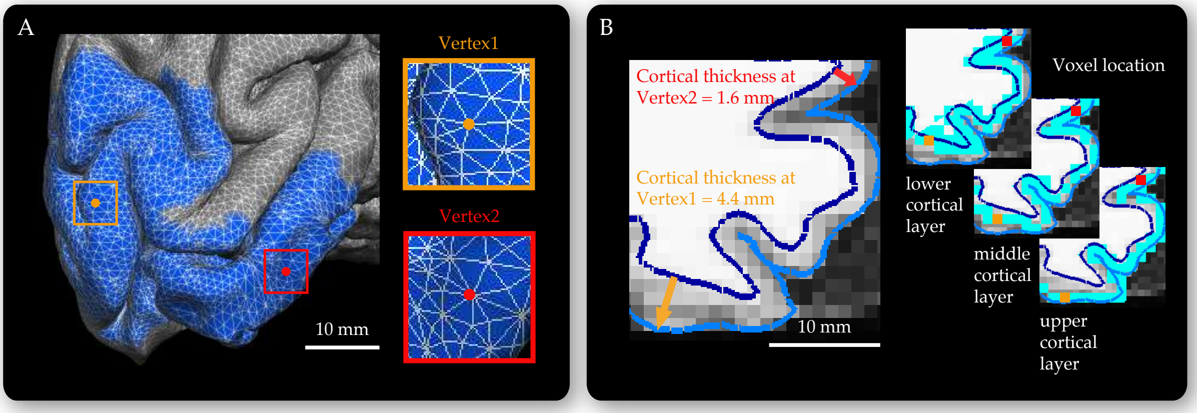

Figure S5. FMRI Spatial Sampling (Related to Experimental Procedures). The triangle-mesh model illustrated the three-dimensional cortical surface reconstruction in a representative participant, where individual vertex of this triangle-mesh model represented a single cortical surface location separable by MRI (A). To assess the influences of fMRI spatial sampling on the measure of neural population tuning, three different voxels, at upper, middle, and lower cortical layers, were sampled for each cortical surface location with high thickness, two different voxels, at upper and lower cortical layers, were sampled for each cortical surface location with medium thickness, and one voxel was sampled for each cortical surface location with low thickness (B). The measure of neural population tuning at individual visual cortical location was compared across voxels at different cortical depth, and the volume of gray matter in individual voxel was compared across different visual cortical locations.

Supplemental Experimental Procedures

1 Participants and apparatus

A group of twenty healthy volunteers gave written informed consent to participate in this study that was approved by the UCL Research Ethics Committee. The participants were young adults (aged 19 to 34, ten females, ten males) with normal or corrected-to-normal vision and no neurological or psychiatric history. All twenty participants took part in the main experiments where we delineated early visual cortices using retinotopic mapping (Sereno et al., 1995), measured visual cortical anatomy using the T1-weighted MRI data, measured neural population tuning for visual field position using the method of population-receptive-field mapping (Dumoulin & Wandell, 2008), and measured perceptual discrimination threshold for visual field position using the psychophysical staircase procedure. Eight (four females, four males) of the twenty participants also took part in the control experiments where we measured visual cortical anatomy using the quantitative-T1 MRI data (Weiskopf et al., 2013), measured fMRI hemodynamic response profile using the visually evoked BOLD responses (Friston et al., 1998; Glover, 1999), and measured fMRI signal-to-noise ratio using the resting state BOLD data (Murphy, Bodurka, & Bandettinia, 2007; Lutti, Thomas, Hutton, & Weiskopf, 2013).

We collected the T1-weighted MRI data at the standard resolution of 1 mm isotropic (TR = 7.92 ms, TE = 2.48 ms, flip angle = 16 degrees, matrix = 256 x 240 x 176), and the quantitative-T1 MRI data at a high resolution of 0.8 mm isotropic as well as the standard resolution of 1 mm isotropic. The protocol for the quantitative-T1 MRI data involved acquisitions of five different MRI signals, and in particular, the proton-density-weighted signals (1 mm resolution: TR = 23.7 ms, TE = 2.2 ms to 19.7 ms by steps of 2.5 ms, flip angle = 6 degrees, matrix = 256 x 240 x 176; 0.8 mm resolution: TR = 25.25 ms, TE = 2.39 ms to 18.91 ms by steps of 2.36 ms, flip angle = 5 degrees, matrix = 320 x 280 x 208), the magnetization-transfer-weighted signals (1 mm resolution: TR = 23.7 ms, TE = 2.2 ms to 14.7 ms by steps of 2.5 ms, flip angle = 6 degrees, matrix = 256 x 240 x 176; 0.8 mm resolution: TR = 29.25 ms, TE = 2.39 ms to 18.91 ms by steps of 2.36 ms, flip angle = 9 degrees, matrix = 320 x 280 x 208), the T1-weighted MDEFT signals (1 mm resolution: TR = 18.7 ms, TE = 2.2 ms to 14.7 ms by steps of 2.5 ms, flip angle = 20 degrees, matrix = 256 x 240 x 176; 0.8 mm resolution: TR = 25.25 ms, TE = 2.39 ms to 18.91 ms by steps of 2.36 ms, flip angle = 29 degrees, matrix = 320 x 280 x 208), the radio-frequency transmit field B1+ signals (1 mm resolution: TR = 500 ms, TE = 18.53 ms / 37.06 ms, flip angle = 90 degrees, matrix = 64 x 48 x 48; 0.8 mm resolution: TR = 500 ms, TE = 19.69 ms / 39.38 ms, flip angle = 90 degrees, matrix = 64 x 48 x 48), and the static magnetic field B0 signals (TR = 1020 ms, TE = 10 ms / 12.46 ms, flip angle = 90 degrees, matrix = 64 x 64 x 64).

We collected the fMRI data at a high spatial resolution of 1.5 mm isotropic, using a 3D EPI sequence with parallel imaging acceleration (Lutti et al., 2013). The data of the main experiments were collected at a standard temporal resolution of 3.2 second volume TR (slice TR = 80 ms, TE = 32.86 ms, flip angle = 20 degrees, field of view = 192 x 192 x 60 mm, 25% oversampling along the encoding slab to avoid wrap-around artifacts). The data of the control experiments were first collected at the same standard temporal resolution of 3.2 second volume TR, and then repeated at a high temporal resolution of 1.52 second volume TR (slice TR = 76 ms, TE = 37.3 ms, flip angle = 15 degrees, field of view = 54 x 192 x 192 mm, 11.1% oversampling along the encoding slab to avoid wrap-around artifacts). The high temporal resolution allowed

fine estimation of fMRI signal properties, but was accompanied by a reduced field-of-view as trade-off. To accommodate the reduced field-of-view, we collected the high temporal resolution fMRI data for the left and the right hemispheres separately in different experimental runs. The fMRI data were preprocessed in SPM8 (http://www.fil.ion.ucl.ac.uk/spm) through bias correction, realignment, unwarping, coregistration, and correction for physiology noise (cardiac activity, respiratory activity, head motion).

The neuroimaging experiments took place in a Siemens Trio 3T MRI scanner with a 32-channel head-coil. Visual stimuli were projected onto a screen (size = 28.6 x 21.5 cm) in the back of the scanner and viewed through a mirror on the head-coil (viewing distance = 85cm). The stimuli covered the visual field extending from fixation to 7.2 degree eccentricity. The psychophysics experiments took place in a dark room where the computer monitor provided the only significant source of light. Visual stimuli were presented on a 22" monitor (size = 41 x 30.6 cm, viewing distance = 67 cm, resolution = 2048 x 1536 pixels, pixel size = 0.017 degree of visual angle) or a 17" monitor (size = 34.2 x 27.5 cm, viewing distance = 3 m, resolution = 1280 x 960 pixels, pixel size = 0.005 degree of visual angle) and viewed through a chin and forehead rest.

2 Delineation of early visual cortices

2.1 Retinotopy-based delineation of early visual cortices

We delineated early visual cortices (V1, V2) non-invasively using the method of phase-encoded retinotopic mapping (Sereno et al., 1995). The mapped visual field covered an eccentricity range from 0.25 to 7.2 degree of visual angle. In the experiment, participants viewed full-contrast flickering checkerboard wedges (radius = 7.2 degree eccentricity) rotating smoothly in clockwise or anti-clockwise direction around a small fixation cross for 10 cycles at a speed of 61.2 seconds per cycle. To maintain participants' attention, at random temporal intervals the checkerboard stimuli underwent a small pattern shift for 200 ms, and participants were asked to indicate whenever this happened with a button press while keeping their eyes fixated at the central cross during the whole experiment. A Fast Fourier Transform was applied to BOLD time series to extract the phase and power at the stimulation frequency. The resulting phase maps were displayed on inflated cortical surfaces reconstructed using FreeSurfer. A statistic map indicating the significance of visual response was calculated by dividing the power at the stimulation frequency with the average power across all frequencies. The polar angle boundaries (representing vertical and horizontal meridians) were delineated manually according to the mirror reversals in the phase map. The eccentricity boundaries (representing 0.25 and 7.2 degree eccentricity) were delineated by thresholding the statistic map at a significance level of $p<0.05$ (uncorrected).

The delineation accuracy of the polar angle boundaries and the eccentricity boundaries were reassured through additional experiments. In particular, to improve the delineation accuracy of polar angle boundaries (representing vertical and horizontal meridians), we compared the polar-angle maps from two different retinotopic mapping experiments using the phase-encoded paradigm (described above) and the population-receptive-field paradigm (described in Supplemental Experimental Procedures Section 4.1), respectively. The comparison was made on a voxel basis for each participant (N = 20). We found that the polar angle values were consistent and correlated between the maps acquired from the two different paradigms (Fig. S1). To improve the delineation accuracy of eccentricity boundaries (representing 0.25 and 7.2 degree

eccentricity), the eccentricity boundaries delineated from the two retinotopic mapping experiments were refined in a third experiment using retinotopic localizer. In the experiment, participants viewed a ring-shaped grating (inner radius = 0.25 degree eccentricity, outer radius = 7.2 degree eccentricity) and a blank screen in a block-design fashion at a speed of 16 seconds per block for 12 blocks per run and 4 runs. Participants maintained their attention and fixation by detecting color change of the central fixation cross. The resulting statistical map was thresholded at $p<0.005$ (uncorrected) to refine the eccentricity boundaries that represented 0.25 and 7.2 degree eccentricity.

2.2 Morphology-based delineation of early visual cortices

As the retinotopy-based delineation covered a part rather than the full extent of early visual cortices, it was potentially confounded by inter-individual differences in the fraction of retinotopy coverage. To address this potential confound, we performed morphology-based delineation where the medial occipital cortex was delineated automatically in Freesurfer according to the cortical folding patterns (Desikan et al., 2006). The morphologically-delineated medial occipital cortex (Freesurfer pericalcarine and cuneus segments) extended along the anterior-posterior axis from the rostral to the caudal ends of the calcarine sulcus, and along the ventral-dorsal axis from the inferomedial end of the calcarine sulcus to the most medial portion of the occipital cortex. Across participants, the morphologically-delineated and the retinotopically-delineated surface area of early visual cortices exhibited correlated inter-individual variability (V1: r = 0.709, $p < 0.001$, N = 20 participants; V2: r = 0.475, $p < 0.05$, N = 20 participants).

The consistency with the morphology-based delineation suggested that the retinotopy-based delineation of early visual cortices was not largely confounded by inter-individual differences in the fraction of retinotopy coverage. To further assess the performance of retinotopic-based delineation, we estimated the fraction of retinotopy coverage for each participant (N = 20), based on the distribution of mapped visual field eccentricity derived from the eccentricity map. This distribution was best fitted with an exponential function $y = ae^{-bx}$. It reflected the percentage of voxels responsive to each visual field eccentricity (Fig. 1B). Given that different voxels were equal in volume, we estimated the retinotopically-delineated part of early visual cortices as the area under the exponential curve from x equaled 0.25 degree eccentricity to x equaled 7.2 degree eccentricity, and the full extent of early visual cortices as the area under the exponential curve from x equaled 0 to x approximated infinite. We found that in both V1 and V2, the retinotopically-delineated part accounted for about three-quarters of the full area. This fraction of retinotopy coverage was rather consistent across participants (V1: mean = 78.3%, std = 3.7%, N = 20; V2: mean = 78.8%, std = 3.3%, N = 20) and did not correlate with inter-individual variability in visual cortical surface area (V1: r = -0.120, p = 0.564, N = 20 participants; V2: r = 0.080, p = 0.768, N = 20 participants). The results reassured us of the reliability of our visual cortical delineation.

3 Visual cortical anatomy

3.1 Influences of MRI analysis software

By applying the surface-based analysis to the T1-weighted MRI data, we measured the thickness at individual visual cortical locations (vertices) and the surface area summed over different visual cortical locations. In the surface-based analysis, the T1-weighted MRI data were preprocessed through skull stripping and non-uniform intensity correction,

after which the pre-processed data were segmented into the white and the gray matter according to intensity-based tissue classification. The white and the gray matter segments were then covered with triangular tessellations to reconstruct the three-dimensional white and pial cortical surfaces in smooth triangle-mesh models. A triangle-mesh model is a collection of vertices (points), edges (connections between vertices), and triangle faces (closet sets of three edges) that defines the shape of a three-dimensional object in geometric modelling. The triangle tessellation was topologically corrected and smoothed using deformable surface algorithm with trilinear interpolation, which allowed the three-dimensional cortical surfaces to be reconstructed at subvoxel accuracy. Based on this three-dimensional cortical surface reconstruction, cortical thickness was computed as the distance between the white and the pial cortical surfaces, and cortical surface area was computed as the summed surface area of triangle faces in a cortical region.

This MRI-based measure of visual cortical anatomy was vulnerable to the confounding influences of data analysis software. To separate the contribution of software specific versus software independent factors, we repeated the analysis in four software, SPM (Ashburner, 2012), Freesurfer (Fischl, 2012), FSL (Jenkinson, Beckmann, Behrens, Woolrich, & Smith, 2012), and MIPAV CBS (http://www.nitrc.org/projects/cbs-tools) (Bogovic, Prince, & Bazin, 2013; Bazin et al., 2013). For each participant, the pre-processing of raw data and the segmentation of cortical tissues were repeated in four different software (SPM, FSL, Freesurfer, MIPAV CBS), after which the reconstruction of cortical surfaces and the computation of cortical thickness were repeated in two different software (Freesurfer, MIPAV CBS).

Across software, we compared the segmentation of cortical tissues, by calculating the standard deviation of the inner cortical boundary (white matter was given the value of one, gray matter the value of zero, the rest of the brain NaN) and that of the outer cortical boundary (gray or white matter was given the value of one, the rest of the brain the value of zero). The standard deviation was calculated on a voxel basis for each participant (N = 20), where the value was 0, or 0.5, or 0.58, if all four, or three, or two software returned the same segmentation result. We also compared the computation of cortical thickness across software, by calculating the correlation in the measure of cortical thickness. Specifically, cortical thickness measured from Freesurfer was plotted against cortical thickness measured from MIPAV CBS, again on a voxel basis. We found that the segmentation standard deviation in occipital lobe (Mazziotta et al., 2001) was low for both the inner cortical boundary (mean = 0.075, std = 0.009, N = 20 participants) and the outer cortical boundary (mean = 0.047, std = 0.009, N = 20 participants). Moreover, the measure of visual cortical thickness was highly correlated between different software ($r = 0.98$, $p < 0.0001$, N = 104158 voxels binned into N = 30 data points according to cortical thickness). Such consistency suggested that the MRI-based measure of visual cortical anatomy was not biased by the specific choice of data analysis software.

3.2 Influences of MRI acquisition sequence

In addition to the concern regarding data analysis software, the MRI-based measure of visual cortical anatomy was potentially vulnerable to the confounding influences of data acquisition sequence. While the T1-weighted MRI sequence we employed is a widely-used standard protocol, the signal in fact represents a combination of magnetic-field-specific and biological-tissue-specific components. Consequently, the T1-weighted MRI images had inhomogenous intensity and low tissue contrast, as illustrated by the mixed peaks of white matter and gray matter in the image intensity histogram (Fig.

S2). This could potentially bias the segmentation of cortical tissues. To address this limitation in quality of the standard T1-weighted MRI images, in control experiments we collected the structural MRI data using a state-of-art quantitative-T1 MRI sequence, at both a high resolution (0.8 mm isotropic voxels, 60 minutes per participant) and a standard resolution (1 mm isotropic voxels, 30 minutes per participant). The quantitative-T1 MRI images were calculated from multiple parametric signals (the proton-density-weighted signals, the magnetization-transfer-weighted signals, the T1-weighted MDEFT signals, the radio-frequency transmit field B1+ signals, the static magnetic field B0 signals) according to the methods developed by (Helms, Dathe, & Dechent, 2008; Weiskopf et al., 2013) including corrections for imperfect spoiling (Preibisch & Deichmann, 2009) and field (B1+, B0) inhomogeneities (Lutti, Hutton, Finsterbusch, Helms, & Weiskopf, 2010; Lutti et al., 2012). Through the consideration of multiple parametric signals, the quantitative-T1 MRI sequence factored out the magnetic-field-specific component and directly reflected the physical property of the underlying biological tissue (Weiskopf et al., 2013). Consequently, the quantitative-T1 MRI images were highly reproducible across different scanning sessions (e.g., 1 mm resolution vs. 0.8 mm resolution) and directly comparable across participants (Fig. S2). Moreover, as illustrated by the clearly separated peaks of white matter and gray matter in the image intensity histogram (Fig. S2), the quantitative-T1 MRI images had high tissue contrast and relatively homogeneous intensity over the cortex. This helped to reduce potential bias in the surface-based analysis.

The surface-based analysis of the quantitative-T1 MRI data were carried out in MIPAV CBS (http://www.nitrc.org/projects/cbs-tools), as the software was developed specifically for high resolution data and was not constrained in the voxel size (Bogovic et al., 2013; Bazin et al., 2013). On a voxel basis, we compared cortical thickness measured from the 1 mm resolution T1-weighted MRI data, the 1 mm resolution quantitative-T1 MRI data, and the 0.8 mm resolution quantitative-T1 MRI data. We found that the measure of visual cortical thickness was consistent across different acquisition sequences and different acquisition resolutions (T1-weighted sequence vs. quantitative-T1 sequence: $r = 0.97$, $p < 0.0001$; 1.0 mm resolution vs. 0.8 mm resolution: $r = 0.97$, $p < 0.0001$; $N = 42538$ voxels binned into $N = 30$ data points according to cortical thickness of 1.0 mm resolution quantitative-T1 MRI data). This observation suggested that although the standard T1-weighted MRI sequence did not offer high image quality, the MRI-based measure of visual cortical anatomy was robust against this limitation.

3.3 Comparison between in-vivo MRI and in-vitro histology

Our control studies reassured us that the MRI-based measure of visual cortical anatomy was not biased by the choice of data analysis software or data acquisition sequence. Nevertheless, while the structural MRI data offer a non-invasive, in-vivo measure of cortical anatomy, the measure is at the same time limited by its indirect nature. In contrast, a direct measure of cortical anatomy (albeit in-vitro) is possible from postmortem histology. Therefore, we further addressed the reliability of our in-vivo MRI measure by comparing it with an in-vitro histology measure derived from postmortem human brain. Conventional analysis of histology data employs a slice-based approach that is constrained by the slice orientation and is consequently limited in sampling coverage. For example, the slice-based measure of cortical thickness is only valid for histology slices orthogonal to the cortical surface. To overcome this limitation, we developed a surface-based histology measure of cortical thickness through reconstructing the three-dimensional triangle-mesh models of the

white and the pial cortical surfaces. The surface-based approach, while demanding, offered a sampling coverage of the full brain that was unconstrained by the slice orientation. This allowed direct comparison in the measure of visual cortical anatomy between the histology data and the structural MRI data.

We applied the surface-based analysis to a dataset of high-resolution (40 μm isotropic pixel), whole-brain (4992 pixel x 3328 pixel), histology images (502 images in total), taken consecutively every 300 μm along the dorsoventral axis of a postmodern human body. The histology data were generously shared by Drs. Yuchun Tang and Shuwei Liu at the Research Center for Sectional and Imaging Anatomy of Shandong University, China, under the approval from the local ethics committee. The postmodern body came from a deceased 38-year-old male who had donated his body for medical research purposes and had no neurological history. After the body was frozen and fixed, the head was dissected from the plane of thyroid cartilage and embedded in blue-stained gelatine. Serial transverse sectioning was performed along the dorsoventral axis using computerised freezing milling technique (milling machine: SKC500, Jinan, China; milling accuracy: 1 μm) (Spitzer, Ackerman, Scherzinger, & Whitlock, 1996). A high-resolution digital camera (Canon EOS 1D MARK II, Japan) was used to take the histology images along with the images of length markers and color charts. The images of length markers and color charts were subsequently used as reference in affine alignment of the histology images. The results of affine alignment were independently checked by two experienced neuroanatomists (Yuchun Tang and Bo Sun) and confirmed through three-dimensional volume reconstruction of the head.

To build the three-dimensional cortical surface models from the two-dimensional histology images, we segmented each histology image into different tissue components. Since performing manual tissue segmentation for a set of 502 images would be labor-intensive and time-consuming, at first we tried to perform automatic tissue segmentation. However, existing algorithms of automatic tissue segmentation were largely based on information of image intensity distribution and were mainly developed for the structural MRI data, yet the histology data differed substantially from the structural MRI data in image intensity distribution. This made MRI-based algorithms unsuitable for analysis of histology data. Indeed, when we applied MRI analysis software (e.g., SPM (Ashburner, 2012), FSL (Jenkinson et al., 2012)) to our histology data, the results of automatic tissue segmentation were rather inaccurate (Fig. S3).

Consequently, we wrote custom code that performed automatic tissue segmentation based on the color information and the consecutive nature of the histology images. We tried several approaches, including using the built-in functions of Mathematica (e.g., ImageForestingComponents, ClusteringComponents), using different saliency detection algorithms (e.g., visual-feature-based, spectral-based), and a combination of both. Nevertheless, neither of these approaches managed to reach satisfactory accuracy. Finally, we resorted to manual tissue segmentation. To improve the segmentation accuracy, we took advantage of the color information of the histology images by performing manual segmentation on the red, green, and blue channel images in addition to the original image. Non-uniform intensity correction (Salvado, Hillenbrand, Zhang, & Wilson, 2006) was also applied, resulting in a total of eight images (corrected/uncorrected red/green/blue/all channel) per transverse section. Based on these images, two researchers (Chen Song and Xiaocheng Sun) performed skull strip and tissue segmentation manually for each transverse section using the software Amira (Stalling, Westerhoff, & Hege, 2005).

Compared to the standard 1 mm resolution of the structural MRI data, the 40 μm in-plane resolution of the histology

data enabled much more accurate tissue segmentation. However, at the same time, the high resolution of the histology data posed challenges for the reconstruction of three-dimensional cortical surface models, because traditional software such as Freesurfer was developed for low resolution data and had constraints regarding voxel size (Fischl, 2012). Nevertheless, the software MIPAV CBS (http://www.nitrc.org/projects/cbs-tools) was developed specifically for high-resolution data and was suitable for our purpose. Using MIPAV CBS, we built the three-dimensional triangle-mesh models of the white and the pial cortical surfaces, from the two-dimensional manual segments of white matter and gray matter. During the analysis, the two-dimensional manual segments were binarized, down-sampled to generate 300 μm isotropic voxels, and refined through three-dimensional topology correction. Based on the three-dimensional cortical surface models, cortical thickness was computed as the distance between the white and the pial cortical surfaces, with the biomechanics of cerebral cortex taken into consideration.

To compare visual cortical thickness measured from the histology data with that measured from the structural MRI data, we delineated the occipital region-of-interest in the histology data according to a cytoarchitectonic atlas (Amunts, Malikovic, Mohlberg, Schormann, & Zilles, 2000) and according to the average retinotopic map from our MRI experiments, respectively. The former approach offered a standardised reference, whereas the later approach improved the comparability between histology-based and MRI-based measure of visual cortical thickness. In both approaches, the regions-of-interests were transformed from the original image space to the histology image space through coregistration and reslice. Regardless of the specific approach, we observed a substantial degree of intra-individual variability in histology-based measure of visual cortical thickness (Fig. S4) that was similar in extent to the MRI-based measure (Fig. 2). Moreover, the dependence of visual cortical thickness on cortical folding and visual field eccentricity that we observed in our structural MRI data was recaptured by our histology data. Specifically, we observed an increase in visual cortical thickness from sulci to gyri for both parafovea (V1: $T = 13.498$, $p < 0.0001$, $N = 87455$ voxels; V2: $T = 18.179$, $p < 0.0001$, $N = 86466$ voxels) and perifovea (V1: $T = 23.822$, $p < 0.0001$, $N = 230387$ voxels; V2: $T = 9.507$, $p < 0.0001$, $N = 50348$ voxels), as well as an increase in visual cortical thickness from parafovea to perifovea for both sulci (V1: $T = 83.929$, $p < 0.0001$, $N = 146834$ voxels; V2: $T = 56.089$, $p < 0.0001$, $N = 72410$ voxels) and gyri (V1: $T = 97.783$, $p < 0.0001$, $N = 171008$ voxels; V2: $T = 49.674$, $p < 0.0001$, $N = 64404$ voxels). This consistency between the MRI-based and the histology-based measures of visual cortical thickness gave us confidence in the application of in-vivo structural MRI data for assessing visual cortical anatomy.

4 Neural population tuning for visual field position

4.1 Measure of position tuning width

Our measure of neural population tuning for visual field position was based on the method of population-receptive-field (pRF) mapping (Dumoulin & Wandell, 2008). In the method, a full-contrast flickered checkerboard bar was presented at 64 different visual field positions, and the BOLD time series of each voxel were deconvolved with hemodynamic response function before fitting with a two-dimensional Gaussian representation of position tuning profile. The two-dimensional Gaussian function quantified the range of visual field positions that the voxel responded to (position tuning width) and the visual field position that the voxel responded strongest to (position tuning peak). This method of

population-receptive-field mapping was conceptually similar to the method of spike-triggered average (reverse correlation) used in extracellular recording for measuring the spatiotemporal receptive field of visual cortical neurons. Both methods relied on the assumption of a linear spatial summation where the response to a stimulus equalled the sum of the responses to the stimulus components. This linearity of spatial summation was observed in BOLD responses from early visual cortices (Hansen, David, & Gallant, 2004), which supported the use of population-receptive-field mapping for estimating neural population tuning.

Each participant (N = 20) took part in two runs of population-receptive-field mapping experiment. In a single experimental run, the checkerboard bar stimulus (width = 1.8 degree of visual angle) moved in the visual field for eight cycles at a speed of sixteen volumes per cycle and one visual field position per volume. The bar stimulus was oriented at one of the four orientations (horizontal, vertical, 45 degree, 135 degree) and moved along the corresponding orthogonal direction (north/south for horizontal bar, west/east for vertical bar, northwest/southeast for 45 degree bar, northeast/southwest for 135 degree bar). The orientation and the moving direction of the bar stimulus were counterbalanced across cycles. A blank screen was inserted into the last quarter of the second, fourth, sixth, and eighth cycle to provide a baseline condition that improved the measurement accuracy. Participants maintained their attention and fixation by detecting color change of the central fixation cross. The BOLD time series of each voxel were deconvolved with hemodynamic response function and then fitted with a two-dimensional Gaussian function f(x_0, y_0, σ) multiplied by the stimulus position function. The peak (x_0, y_0) and the width (σ) of position tuning were estimated for each voxel by varying the parameters x_0, y_0 (limit = 7.2 degree eccentricity) and σ (lower limit = 0.001 degree of visual angle, upper limit = 7.2 degree of visual angle) to find the least square fit. The measure of position tuning width was not confounded by the goodness-of-fit (r = -0.001, p = 0.83) and the voxels with a goodness-of-fit smaller than 15% were excluded in later analysis.

4.2 Influences of fMRI spatial sampling

The voxel-level measure of neural population tuning reflected a combined contribution from the average position tuning width of neurons in the voxel and the heterogeneity in position tuning peak between different neurons in the voxel (Hubel & Wiesel, 1974). To improve the resolution of the measure and minimise intra-voxel heterogeneity in tuning peak, we collected the fMRI data at a high spatial resolution (1.5 mm isotropic voxel) using a 3D EPI sequence with parallel imaging acceleration (Lutti et al., 2013). For tissue volumes as small as 1.5 mm isotropic, the heterogeneity in position tuning peak is smaller than position tuning width of single neurons, and is correlated with the average position tuning width of neurons in the tissue volume (Hubel & Wiesel, 1974). Therefore, the voxel-level measure of position tuning width in effect reflected the average tuning width of neurons in the voxel. Indeed, this voxel-level position tuning width (0.6$\pm$0.35 degree of visual angle) measured here in the retinotopically-delineated part of human primary visual cortex was comparable with neural-level position tuning width (0.35 degree of visual angle) in the corresponding part of macaque primary visual cortex (Hubel & Wiesel, 1974).

In addition to minimising intra-voxel heterogeneity in position tuning peak, the high spatial resolution of our fMRI data also contributed to minimising inter-voxel heterogeneity in spatial sampling. To assess the confounding influences of spatial sampling, three different voxels, at upper, middle, and lower cortical layers, were sampled for each visual

cortical location with high cortical thickness, two different voxels, at upper and lower cortical layers, were sampled for each visual cortical location with medium cortical thickness, and one voxel was sampled for each visual cortical location with low cortical thickness (Fig. S5). We found that the measure of position tuning at individual visual cortical location was consistent across voxels at different cortical depth. Moreover, the gray matter volume in individual voxel was homogeneous across different visual cortical locations. These observations suggested that inter-voxel variability in position tuning width was not an artefact of fMRI spatial sampling.

4.3 Influences of fMRI hemodynamic coupling

Despite the improvement in spatial resolution, the fMRI-based measure of neural population tuning was nonetheless still potentially confounded by the temporal lag between neural responses and fMRI signals due to hemodynamic coupling. This potential confounding factor was taken into consideration during the experiment where the BOLD time series of each voxel were deconvolved with hemodynamic response function before fitting with a two-dimensional Gaussian representation of position tuning profile. In the main experiments, we took a classical fMRI analytic approach where a canonical hemodynamic response function $h(t) = (t/5.4)^6 e^{-(t-5.4)/0.9} - 0.35 * (t/10.8)^{12} e^{-(t-10.8)/0.9}$ was used for all participants and all voxels (Friston et al., 1998; Glover, 1999). Since the volume TR (3.2 second) in our experiment was no smaller than the full-width-half-maximum and the time-to-peak of hemodynamic response function in early visual cortices (Siero, Petridou, Hoogduin, Luijten, & Ramsey, 2011), the effective hemodynamic response function, namely the discrete function sampled every volume TR, was a Dirac delta function that temporally lagged position tuning peak for one volume without influencing position tuning width. Therefore, in theory, inter-voxel variability in position tuning width could not be an artefact of hemodynamic variability.

Nevertheless, to improve the reliability of position tuning measure, in control experiments we estimated the hemodynamic response function for individual voxels through acquisition of the visually evoked BOLD responses (Friston et al., 1998; Glover, 1999). We collected the visually evoked BOLD responses, first using the original 3D EPI sequence with the standard temporal resolution (volume TR = 3.2 second), and then using a new 3D EPI sequence with a high temporal resolution (volume TR = 1.52 second). This high temporal resolution allowed a fine estimation of the hemodynamic response function. For the 3D EPI sequences with volume TR of 3.2 and 1.52 second, a full-contrast flickered checkerboard ring (inner radius = 0.25 degree eccentricity, outer radius = 7.2 degree eccentricity) was presented for 3.2 and 3.04 second, followed by a blank screen of 28.8 and 27.36 second, and repeated for 20 and 40 cycles, respectively. Participants maintained their attention and fixation by detecting color change of the central fixation cross. The BOLD time series of each voxel were fitted with a hemodynamic response function $h(t) = (t/t_1)^{(5.6*t_1^2/w_1^2)} e^{-(t-t_1)*(5.6*t_1/w_1^2)} - d * (t/t_2)^{(5.6*t_2^2/w_2^2)} e^{-(t-t_2)*(5.6*t_2/w_2^2)}$, where the peak t_1, t_2 (limit = 12 second), the full-width-half-maximum w_1, w_2 (limit = 30 second), and the dip d (lower limit = 0.2, upper limit = 1) were varied to find the least square fit.

The parameters of this voxel-level optimized hemodynamic response function (mean of t_1 = 5.4, mean of t_2 = 10.7, mean of w_1 = 5.3, mean of w_2 = 7.8, mean of d = 0.36, N = 42538 voxels) were close in value to the parameters of canonical hemodynamic response function (t_1 = 5.4, t_2 = 10.8, w_1 = 5.2, w_2 = 7.4, d = 0.35). Moreover, the parameters exhibited a rather small degree of inter-voxel variability (std of t_1 = 0.006, std of t_2 = 0.029, std of w_1 = 0.008, std of w_2 =

0.027, std of d = 0.001, N = 42538 voxels) that did not co-vary with the measure of position tuning width (t_1: r = -0.107, p = 0.432; t_2: r = -0.045, p = 0.748; w_1: r = -0.049, p = 0.746; w_2: r = -0.030, p = 0.812; d: r = -0.002, p = 0.978; N = 42538 voxels binned into N = 30 data points according to position tuning with). These observations suggested that the canonical hemodynamic response function used in our main experiments was representative of the voxel-level estimation of hemodynamic response functions.

To further improve the reliability of position tuning measure, we reanalysed the data from the population-receptive-field mapping experiment using the voxel-level estimation of hemodynamic response function rather than the canonical hemodynamic response function. We deconvolved the BOLD time series of each voxel with the hemodynamic response function $h(t) = (t/t_1)^{(5.6*t_1^2/w_1^2)} e^{-(t-t_1)*(5.6*t_1/w_1^2)} - d*(t/t_2)^{(5.6*t_2^2/w_2^2)} e^{-(t-t_2)*(5.6*t_2/w_2^2)}$ whose parameters (t_1, t_2, w_1, w_2, d) were estimated from the visually evoked BOLD responses acquired through control experiments. The rest of the analysis followed the same procedure as before, where the deconvolved BOLD time series of each voxel were fitted with a two-dimensional Gaussian representation of position tuning profile f(x_0, y_0, σ) multiplied by the stimulus position function. We found that position tuning width measured using this voxel-level optimized hemodynamic response function was consistent with that measured using canonical hemodynamic response function (r = 0.9355, p $<$ 0.0001, N = 42538 voxels binned into N = 30 data points according to position tuning width).

This new analysis suggested that our original analysis of neural population tuning was not biased by inter-voxel variability in hemodynamic response function. Nevertheless, both the new and the original analysis were dependent on the application of hemodynamic response model $h(t, t_1, t_2, w_1, w_2, d)$. To test whether the choice of hemodynamic response model might confound the measure of neural population tuning, we further conducted hemodynamic-model-free analysis where the BOLD time series from the population-receptive-field mapping experiment were deconvolved directly with the time series of visually evoked BOLD responses. We found that position tuning width measured from this model-free analysis and position tuning width measured from the model-based analysis exhibited high level of consistency (r = 0.9394, p $<$ 0.0001, N = 42538 voxels binned into N = 30 data points according to position tuning width), suggesting that the measure was not biased by the choice of hemodynamic response model. Together these results suggested that inter-voxel variability in position tuning width more likely reflected neural variability rather than hemodynamic variability.

4.4 Influences of fMRI signal-to-noise ratio

In addition to the confounding influences of hemodynamic coupling, the fMRI-based measure of neural population tuning was also potentially affected by fMRI signal-to-noise ratio. To assess fMRI signal-to-noise ratio, in control experiments we acquired the resting state BOLD data, using the original 3D EPI sequence with standard temporal resolution (volume TR = 3.2 second) as well as the new 3D EPI sequence with a high temporal resolution (volume TR = 1.52 second). A single experiment run lasted for 10 minutes, during which participants maintained awake through eye fixation at a central cross on a black screen, under the monitor of an eye tracker. From the resting state BOLD time series, we calculated fMRI signal-to-noise ratio on a voxel basis, before and after physiological noise correction, respectively. The signal-to-noise ratio was calculated as the mean divided by the standard deviation of the BOLD time series (Murphy et al., 2007; Lutti et al., 2013).

We found that fMRI signal-to-noise ratio did not vary systematically with the measure of position tuning width (r = -0.229, p = 0.114, N = 42538 voxels binned into N = 30 data points according to position tuning width), or with cortical thickness (r = 0.106, p = 0.466, N = 42538 voxels binned into N = 30 data points according to cortical thickness), or with cortical folding (T = 0.061, p = 0.952, N = 42538 voxels). Moreover, while the physiological noise correction significantly improved fMRI signal-to-noise ratio by 35.95%, the degree of improvement did not co-vary with the measure of position tuning width (r = -0.219, p = 0.144, N = 42538 voxels binned into N = 30 data points according to position tuning width), or with cortical thickness (r = -0.004, p = 0.994, N = 42538 voxels binned into N = 30 data points according to cortical thickness), or with cortical folding (T = -0.021, p = 0.983, N = 42538 voxels). These results suggested that inter-voxel variability in position tuning width did not arise from variability in fMRI signal-to-noise ratio.

5 Perceptual discrimination for visual field position

5.1 Measure of position discrimination threshold

We measured the threshold of perceptual discrimination for visual field position using a psychophysical paradigm of spatial comparison, where participants judged the position difference between two concurrently presented stimuli (Vernier stimuli). In a single experimental trial, a pair of collinear bars and a pair of horizontally offset bars were presented in succession on the computer screen with random order (single bar width = 0.15 degree of visual angle, single bar length = 0.6 degree of visual angle, vertical distance between two bars = 0.3 degree of visual angle). The duration of each bar pair was 300 ms and the inter-stimulus-interval was 500 ms. While maintaining central fixation throughout the experiment, participants made an unspeeded forced choice regarding which temporal interval contained the pair of horizontally offset bars. The horizontal position difference between the pair of horizontally offset bars was varied in a 2-up-1-down staircase to assess the threshold at which the performance converged to 70.7% correct. Two consecutive correct answers led to a one-step decrease in the position difference in the next trial, whereas one incorrect answer led to a one-step increase in the position difference. The experiment stopped after eighteen reversals, and position discrimination threshold was calculated as the horizontal position difference averaged over the last ten reversals.

To test the reliability of position discrimination measure, in a separate experiment we employed a psychophysical paradigm of temporal comparison, where participants judged the position difference between two sequentially presented stimuli. In a single experimental trial, two bars were presented in succession on the computer screen where the duration of each bar was 300 ms and the inter-stimulus-interval was 500 ms (bar width = 0.15 degree of visual angle, bar length = 1.5 degree of visual angle). While maintaining central fixation throughout the experiment, participants made an unspeeded forced choice regarding whether the second bar, compared with the first bar, was moved horizontally rightwards or leftwards. The horizontal position difference between the two bars was varied in a 2-up-1-down staircase to assess the threshold at which the performance converged to 70.7% correct. For each participant (N = 20), we measured position discrimination threshold at central fixation (zero eccentricity) using the two different paradigms. We found that position discrimination threshold measured from the spatial and the temporal comparison paradigm was correlated across participants (r = 0.652, $p < 0.01$, N = 20 participants), suggesting that the measure was robust to the psychophysical paradigm.

5.2 Cortical projection of position discrimination threshold

To acquire a cortical map of perceptual acuity, we measured position discrimination threshold at a set of non-overlapping, evenly distributed visual field positions, using the psychophysical paradigm of spatial comparison. For each individual visual field position, we sought to acquire as accurate a measure of position discrimination threshold as possible such that the minimum position difference in the visual stimuli was smaller than participants' minimum position discrimination threshold. At the same time, we hoped to achieve a large visual field coverage that was at least no smaller than the visual field coverage in our fMRI experiments (i.e., central 7.2 degree eccentricity). However, there was a natural trade-off between the spatial resolution and the visual field coverage of any visual display. Therefore, we applied two different setup, one 17" monitor that offered a high spatial resolution (pixel size = 0.005 degree of visual angle) yet a limited visual field coverage (radius = 2.4 degree of visual angle), and one 22" monitor that offered a large visual field coverage (radius = 12.9 degree of visual angle) yet a limited spatial resolution (pixel size = 0.017 degree of visual angle). Balancing the available setup with our needs, we measured position discrimination thresholds at thirteen non-overlapping visual field positions covering three eccentricities (0, 4.7, 6.7 degree) and six polar angles (45, 90, 135, 225, 270, 315 degree). The measure at each visual field position was carried out in an independent experiment. We used the 17" monitor to measure position discrimination threshold at the central visual field (zero eccentricity) and the 22" monitor to measure position discrimination threshold at the peripheral visual field (4.7, 6.7 degree eccentricity).

The measure of position discrimination threshold was then projected onto early visual cortices to generate a cortical map of perceptual acuity. Specifically, the measure at each visual field position was attributed to the corresponding visual cortical locations (vertices) whose extent of position tuning overlapped with that visual field position. In the rare case where more than one position discrimination threshold was attributed to a single visual cortical location, the average of these thresholds was used. When determining whether the extent of position tuning overlapped with a visual field position, we applied four different criteria. The effective visual field position was taken as the area covering either the ends of bar stimuli (extending 0.2 degree of visual angle from the ends of the two bars) or the entire bar stimuli (extending 0.75 degree of visual angle from the stimulus center). The effective position tuning was cut-off at either 2.5σ (where the activity fell to 5% of the maximum activity) or 1.5σ (where the activity fell to 1/e of the maximum activity). Under the four different criteria, the cortical projection of position discrimination threshold covered 55%, 45%, 70%, 60% of the cortical locations in V1, respectively, and 60%, 50%, 90%, 80% of the cortical locations in V2, respectively. In the main manuscript, we reported the results where the effective visual field position was taken as the area covering the ends of bar stimuli and the effective position tuning was cut-off at 2.5σ. However, the specific criterion applied did not affect the results.

References

Amunts, K., Malikovic, A., Mohlberg, H., Schormann, T., & Zilles, K. (2000). Brodmann's areas 17 and 18 brought into stereotaxic space-where and how variable? *Neuroimage*, 66-84.

Ashburner, J. (2012). Spm: A history. *NeuroImage*, *62*, 791-800.

Bazin, P. L., Weiss, M., Dinse, J., Schafer, A., Trampel, R., & Turner, R. (2013). A computational framework for ultra-high resolution cortical segmentation at 7 tesla. *Neuroimage*, 10.1016.

Bogovic, J. A., Prince, J. L., & Bazin, P. L. (2013). A multiple object geometric deformable model for image segmentation. *Computer Vision and Image Understanding*, *117*, 145-157.

Desikan, R. S., Segonne, F., Fischl, B., Quinn, B. T., Dickerson, B. C., Blacker, D., et al. (2006). An automated labeling system for subdividing the human cerebral cortex on mri scans into gyral based regions of interest. *Neuroimage*, *31*, 968-980.

Dumoulin, S. O., & Wandell, B. A. (2008). Population receptive field estimates in human visual cortex. *Neuroimage*, *39*, 647-660.

Fischl, B. (2012). Freesurfer. *NeuroImage*, *62*, 774-781.

Friston, K. J., Fletcher, P., Josephs, O., Holmes, A., Rugg, M. D. M., & Turner, R. (1998). Event-related fmri: characterizing differential responses. *Neuroimage*, *7*, 30-40.

Glover, G. H. (1999). Deconvolution of impulse response in event-related bold fmri. *Neuroimage*, *9*, 416-429.

Hansen, K. A., David, S. V., & Gallant, J. L. (2004). Parametric reverse correlation reveals spatial linearity of retinotopic human v1 bold response. *Neuroimage*, *23*, 233-241.

Helms, G., Dathe, H., & Dechent, P. (2008). Quantitative flash mri at 3t using a rational approximation of the ernst equation. *Magnetic Resonance in Medicine*, *59*, 667-672.

Hubel, D. H., & Wiesel, T. N. (1974). Uniformity of monkey striate cortex: a parallel relationship between field size, scatter, and magnification factor. *The Journal of comparative neurology*, *158*, 295-306.

Jenkinson, M., Beckmann, C. F., Behrens, T. E., Woolrich, M. W., & Smith, S. M. (2012). Fsl. *NeuroImage*, *62*, 782-790.

Lutti, A., Hutton, C., Finsterbusch, J., Helms, G., & Weiskopf, N. (2010). Optimization and validation of methods for mapping of the radiofrequency transmit field at 3t. *Magnetic Resonance in Medicine*, *64*, 229-238.

Lutti, A., Stadler, J., Josephs, O., Windischberger, C., Speck, O., Bernarding, J., et al. (2012). Robust and fast whole brain mapping of the rf transmit field b1 at 7t. *PLoS One*, *7*, e32379.

Lutti, A., Thomas, D. L., Hutton, C., & Weiskopf, N. (2013). High-resolution functional mri at 3 t: 3d/2d echo-planar imaging with optimized physiological noise correction. *Magnetic Resonance in Medicine*, 1657-1664.

Mazziotta, J., Toga, A., Evans, A., Fox, P., Lancaster, J., Zilles, K., et al. (2001). A probabilistic atlas and reference system for the human brain: International consortium for brain mapping (icbm). *Computer Vision and Image Understanding*, *356*, 1293-1322.

Murphy, K., Bodurka, J., & Bandettinia, P. A. (2007). How long to scan? the relationship between fmri temporal signal to noise ratio and necessary scan duration. *Neuroimage*, 565-574.

Preibisch, C., & Deichmann, R. (2009). Influence of rf spoiling on the stability and accuracy of t1 mapping based on spoiled flash with varying flip angles. *Magnetic Resonance in Medicine*, *61*, 125-135.

Salvado, O., Hillenbrand, C., Zhang, S., & Wilson, D. L. (2006). Method to correct intensity inhomogeneity in mr images for atherosclerosis characterization. *IEEE Transactions on Medical Imaging*, *25*, 539-552.

Sereno, M. I., Dale, A. M., Reppas, J. B., Kwong, K. K., Belliveau, J. W., Brady, T. J., et al. (1995). Borders of multiple visual areas in human revealed by functional magnetic resonance imaging. *Science*, *268*, 889-893.

Siero, J. C. W., Petridou, N., Hoogduin, H., Luijten, P. R., & Ramsey, N. F. (2011). Cortical depth-dependent temporal dynamics of the bold response in the human brain. *Journal of Cerebral Blood Flow Megabolism*, *31*, 1999-2008.

Spitzer, V., Ackerman, M. J., Scherzinger, A. L., & Whitlock, D. (1996). The visible human male: a technical report. *Journal of the American Medical Informatics Association*, *3*, 118-130.

Stalling, D., Westerhoff, M., & Hege, H. C. (2005). Amira: A highly interactive system for visual data analysis. *The Visualization Handbook (Elsevier)*, 749-767.

Weiskopf, N., Suckling, J., Williams, G., Correia, M. M., Inkster, B., Tait, R., et al. (2013). Quantitative multi-parameter mapping of r1, pd(*), mt, and r2(*) at 3t: a multi-center validation. *Frontiers in Neuroscience*, *78*, doi: 10.3389.
