# Supplementary material for: Neural Population Tuning Links Visual Cortical Anatomy to Human Visual Perception
Source: Neuron. 2015 Feb 4;85(3):641–56. doi: 10.1016/j.neuron.2014.12.041 (PMC4321887; doi:10.1016/j.neuron.2014.12.041)
Supplement: Document S2. Article plus Supplemental Information [file mmc2.pdf]

Article

Neuron

Neural Population Tuning Links Visual Cortical Anatomy to Human Visual Perception

Highlights

- Variability in cortical thickness and surface area has opposite functional impacts
- Smaller human visual cortical thickness links to high neural and perceptual acuity
- Larger human visual cortical surface area links to high neural and perceptual acuity

Authors

Chen Song, Dietrich Samuel Schwarzkopf, Ryota Kanai, Geraint Rees

Correspondence

chen.song.09@ucl.ac.uk

In Brief

Song et al. showed that large brains are not necessarily advantageous. Instead, the two dimensions, thickness and surface area, of human brain have opposite impacts on visual perception. A perceptually advantageous brain is a thinned one with enlarged surface area.

Song et al., 2015, Neuron *85*, 641–656
February 4, 2015 ©2015 The Authors
http://dx.doi.org/10.1016/j.neuron.2014.12.041

CellPress

Neural Population Tuning Links Visual Cortical Anatomy to Human Visual Perception

Chen Song,[1,2,*] Dietrich Samuel Schwarzkopf,[1,2] Ryota Kanai,[1,3] and Geraint Rees[1,2]
[1]Institute of Cognitive Neuroscience, University College London, 17 Queen Square, London WC1N 3AR, UK
[2]Wellcome Trust Centre for Neuroimaging, University College London, 12 Queen Square, London WC1N 3BG, UK
[3]School of Psychology, University of Sussex, Sussex House, Brighton BN1 9QH, UK
*Correspondence: chen.song.09@ucl.ac.uk
http://dx.doi.org/10.1016/j.neuron.2014.12.041
This is an open access article under the CC BY license (http://creativecommons.org/licenses/by/4.0/).

SUMMARY

The anatomy of cerebral cortex is characterized by two genetically independent variables, cortical thickness and cortical surface area, that jointly determine cortical volume. It remains unclear how cortical anatomy might influence neural response properties and whether such influences would have behavioral consequences. Here, we report that thickness and surface area of human early visual cortices exert opposite influences on neural population tuning with behavioral consequences for perceptual acuity. We found that visual cortical thickness correlated negatively with the sharpness of neural population tuning and the accuracy of perceptual discrimination at different visual field positions. In contrast, visual cortical surface area correlated positively with neural population tuning sharpness and perceptual discrimination accuracy. Our findings reveal a central role for neural population tuning in linking visual cortical anatomy to visual perception and suggest that a perceptually advantageous visual cortex is a thinned one with an enlarged surface area.

INTRODUCTION

The cerebral cortex is a neural sheet composed vertically of ontogenetic cortical columns and horizontally of laminar layers (Rakic, 1988; Mountcastle, 1997). The surface area of cerebral cortex depends on the proliferation of cortical columns, whereas the thickness of cerebral cortex depends on the generation of laminar layers (Rakic, 1974; Bugbee and Goldman-Rakic, 1983; Jones, 2000). These two elementary dimensions, cortical thickness and cortical surface area, jointly determine cortical volume. However, controlled by independent sets of genetic-developmental factors, cortical thickness and cortical surface area exhibit distinct patterns of variability (Rakic, 1988; Panizzon et al., 2009; Joyner et al., 2009; Chen et al., 2011). Cortical surface area has expanded over 1,000-fold from small mammals to humans (Blinkov and Glezer, 1968; Rakic, 1988). Even within the human species, the surface area of a cortical region, such as visual cortical surface area, can vary up to 3-fold across healthy adults (Dougherty et al., 2003). By contrast, cortical thickness has only doubled during mammalian evolution and differs marginally across human individuals (Blinkov and Glezer, 1968; Rakic, 1988). Nevertheless, cortical thickness can vary over 3-fold across different cortical locations within the same cortical region of the same individual (Fischl and Dale, 2000; Hilgetag and Barbas, 2006).

This substantial variability in cortical anatomy has attracted great interest in the study of its behavioral consequences, and recent progress has been made in identifying correlations between higher performance on a variety of behavioral tasks and larger local cortical volume in task-relevant cortical regions (Kanai and Rees, 2011). However, the fundamental questions of whether a larger cortical volume is in essence behaviorally advantageous and why cortical volume is behaviorally relevant remain to be addressed. An intuitive hypothesis is that increases in cortical volume, arising either from increased cortical thickness or cortical surface area, improve behavioral performance by engaging responses from more neurons (Haug, 1987) and increasing the overall signal-to-noise ratio (Gilbert et al., 2001). Alternatively, changes in behavioral performance may be driven by changes in neural response properties that are associated with variation in local cortical volume and potentially associated differently with cortical thickness versus cortical surface area, as these two anatomical dimensions exhibit distinct natures that affect different aspects of intracortical processing (Kaas, 2000). Specifically, cortical thickness characterizes local (point-level) cortical anatomy, where the thickness at different cortical locations within a cortical region can be independently assessed and reflects the result of tissue proliferation. By contrast, cortical surface area characterizes global (region-level) cortical anatomy, where the surface area of a cortical region is determined jointly by the set of cortical locations it bounds and reflects the result of cortical arealization. As such, variability in the surface area of a cortical region might globally influence all the cortical columns within that region and intercolumnar processing between them, whereas variability in the thickness at a cortical location might locally influence the cortical column at that location and interlaminar processing within it.

To test our two hypotheses, we therefore investigated whether cortical thickness and cortical surface area, which both contribute to cortical volume, had similar or different functional impacts for neural response properties and human behavioral performance. In human cerebral cortex, the neural response

CrossMark

properties of many cortical regions are hard to characterize using noninvasive neuroimaging techniques. Due to the limited spatial resolution of fMRI signals, different neurons within a single fMRI voxel tend to exhibit heterogeneous response properties that often render the voxel-level characterization of neural responses qualitatively different from the response properties of single neurons. An exception to this limitation is early retinotopic visual cortices. Neurons in early visual cortices respond to visual field position in an orderly fashion, where cortically adjacent neurons are tuned to spatially adjacent visual field positions (Hubel and Wiesel, 1974; Sereno et al., 1995). This relative similarity in tuning responses between different neurons within a single fMRI voxel allows fMRI-based characterization of neural population tuning (Dumoulin and Wandell, 2008; Fischer and Whitney, 2009). Given the close correspondence between neural tuning properties and perceptual discrimination performance (Purushothaman and Bradley, 2005), this fMRI-based characterization of neural population tuning enabled us to explore the behavioral significance of any influence that cortical anatomy may have on neural response properties.

Using human early visual cortices (V1 and V2) as a model system, we investigated how visual cortical thickness and visual cortical surface area influenced neural population tuning for visual field position, and whether such influences had behavioral consequences on perceptual discrimination for visual field position. Since visual cortical thickness captures the differences between visual field positions in the cortical architecture at corresponding visual cortical locations, we studied how thickness at different visual cortical locations related to the width of neural population tuning and the0 of perceptual discrimination for corresponding visual field positions. On the other hand, since visual cortical surface area reflects the differences between individuals in the proportion of cortex devoted to early visual processing, we studied how surface area of early visual cortices influenced the position tuning width and position discrimination threshold across the visual field in general.

RESULTS

For a group of 20 healthy participants, we used structural MRI, high-resolution fMRI, and visual psychophysics to measure anatomy of early visual cortices (V1 and V2), population tuning of visual cortical neurons, and performance in perceptual discrimination, respectively. The psychophysical experiments assessing perceptual discrimination were conducted outside the scanner, while the neuroimaging experiments assessing visual cortical anatomy and neural population tuning were performed inside the scanner. During data analysis, we explored the relationships among these independent measures reflecting cortical anatomy, neural response properties, and behavioral performance, respectively.

Variability in Visual Cortical Anatomy

Delineation of early visual cortices (V1 and V2) used the standard method of retinotopic mapping (Sereno et al., 1995). The mapped visual field covered an eccentricity range from 0.25 to 7.2 degree of visual angle. To improve the delineation accuracy of polar angle boundaries (representing vertical and horizontal meridians), we conducted two different retinotopic mapping experiments using phase-encoded paradigm (Sereno et al., 1995) and population-receptive-field paradigm (Dumoulin and Wandell, 2008), respectively. To improve the delineation accuracy of eccentricity boundaries (representing 0.25 and 7.2 degree eccentricity), the eccentricity boundaries delineated from the two retinotopic mapping experiments were refined in a third experiment using a retinotopic localizer. The performance of retinotopic-based delineation was assessed through comparison with morphology-based delineation, where the medial occipital cortex was segmented according to the cortical folding patterns (Desikan et al., 2006). We found that the delineation of early visual cortices was consistent across different delineation protocols (Figure S1 available online; Supplemental Experimental Procedures section 2).

After the delineation of early visual cortices, we measured visual cortical thickness and visual cortical surface area by applying the surface-based analysis on the structural MRI data collected using the standard T1-weighted MRI sequence. In the analysis, the structural MRI data were segmented into different cortical tissues, from which the 3D cortical surface was reconstructed in a smooth triangle-mesh model, with each vertex of this mesh representing a single cortical location distinguishable by MRI (Dale et al., 1999). Based on this 3D cortical surface reconstruction, we measured the thickness at individual visual cortical locations (vertices) and the surface area summed over different visual cortical locations. The MRI-based measure of visual cortical anatomy was potentially confounded by the choice of data analysis software. Therefore, we repeated the analysis in four established software packages, SPM (Ashburner, 2012), Freesurfer (Fischl, 2012), FSL (Jenkinson et al., 2012), and MIPAV CBS (Bazin et al., 2013), in order to separate the contributions of software specific versus software independent factors. We found that the MRI-based measure of visual cortical anatomy was not biased by the specific choice of data analysis software (Supplemental Experimental Procedures section 3.1).

Our MRI-based measure of visual cortical anatomy was also potentially vulnerable to any confounding influences of data acquisition sequence. Specifically, while the T1-weighted MRI sequence we employed is a widely used standard protocol, the signal in fact represents a combination of magnetic-field-specific and biological-tissue-specific components. As a result, the T1-weighted MRI images had inhomogenous intensity and low tissue contrast that could potentially lead to bias in the segmentation of cortical tissues. To address this limitation in quality of the standard T1-weighted MRI sequence, in control experiments, we collected the structural MRI data using a state-of-art quantitative-T1 MRI sequence, at both a high resolution (0.8 millimeter [mm] isotropic voxels) and a standard resolution (1 mm isotropic voxels). Through the detection of multiple parametric signals, the quantitative-T1 MRI sequence factored out the magnetic-field-specific component and directly reflected the physical property of the underlying biological tissue (Weiskopf et al., 2013). As such, the quantitative-T1 MRI images had homogeneous intensity and high tissue contrast that greatly reduced potential bias in the surface-based analysis (Figure S2). Although the standard T1-weighted MRI sequence had image

CellPress

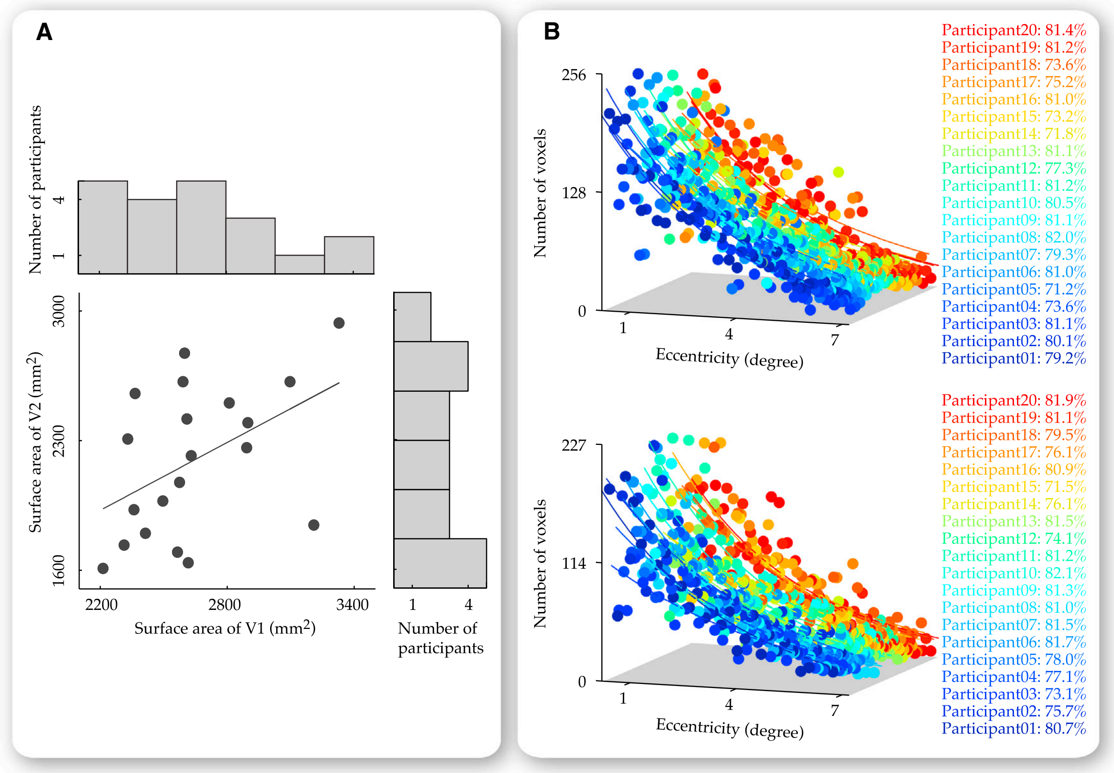

Figure 1. Variability in Visual Cortical Surface Area

Variability in visual cortical surface area was studied in a group of 20 participants, where we applied the standard method of retinotopic mapping to delineate the part of early visual cortices (V1 and V2) that responded to the visual field between 0.25 and 7.2 degree eccentricity. Based on the retinotopy delineation, visual cortical surface area was calculated as the surface area summed over all cortical locations in the retinotopically delineated part of V1 or V2. This retinotopically delineated visual cortical surface area exhibited a 2-fold interindividual variability (illustrated in the marginal histogram of A) that was correlated between V1 and V2 (illustrated in the scatter plot of A). To quantify the fraction of retinotopically delineated V1 or V2 to full V1 or V2, the distribution of mapped visual field eccentricity was plotted on a voxel basis, where voxels responsive to similar eccentricity were binned to generate 30 data points for each participant (B). From the exponential fit to the eccentricity distribution, we estimated the retinotopically delineated V1 or V2 as the area under the exponential fit between x equaled 0.25 and x equaled 7.2, and the full V1 or V2 as the area under the exponential fit between x equaled 0 and x approximated infinite. Data points are color coded according to the participant (B). Parameters reflect the fraction of retinotopically delineated V1 or V2 (B).

quality limitations, we found that the MRI-based measure of visual cortical anatomy was nonetheless robust against such limitations and was strongly correlated across different data acquisition sequences (Supplemental Experimental Procedures section 3.2).

From our MRI-based measure of visual cortical anatomy, we studied variability in visual cortical surface area and visual cortical thickness. Consistent with previous reports (Dougherty et al., 2003), the retinotopically delineated visual cortical surface area exhibited a 2-fold interindividual variability (Figure 1A, summed across left and right hemispheres; V1, 2,213 mm^2 to 3,328 mm^2 and V2, 1,611 mm^2 to 2,936 mm^2) that was correlated between V1 and V2 (Figure 1A; $r = 0.568$, $p < 0.05$, $n = 20$). As the retinotopy delineation covered a part rather than the full extent of early visual cortices, we further explored interindividual variability in the fraction of retinotopy coverage, based on the distribution of mapped visual field eccentricity derived from the eccentricity map. This distribution was best fitted with an exponential function $y = ae^{-bx}$, which reflected the percentage of voxels responsive to each visual field eccentricity (Figure 1B). Given that different voxels were equal in volume, we estimated the retinotopically delineated part of early visual cortices as the area under the exponential curve from x equaled 0.25 degree eccentricity to x equaled 7.2 degree eccentricity, and the full extent of early visual cortices as the area under the exponential curve from x equaled 0 to x approximated infinite. We found that in both V1 and V2, the retinotopically delineated part accounted for about three-quarters of the full area. This fraction of retinotopy coverage was rather consistent across participants (V1, mean = 78.3%, SD = 3.7%, n = 20 and V2, mean = 78.8%, SD = 3.3%, n = 20) and did not correlate with the measure of visual cortical surface area (V1, $r = -0.120$, $p = 0.564$, $n = 20$ and V2, $r = 0.080$, $p = 0.768$, $n = 20$). Therefore, we concluded that the retinotopically delineated visual cortical surface area captured true anatomical variability.

Compared to the large degree of interindividual variability in visual cortical surface area, the average thickness of early visual cortices varied across participants to a much smaller degree from 2 mm to 2.5 mm. Nevertheless, within individual participants, visual cortical thickness varied across different visual

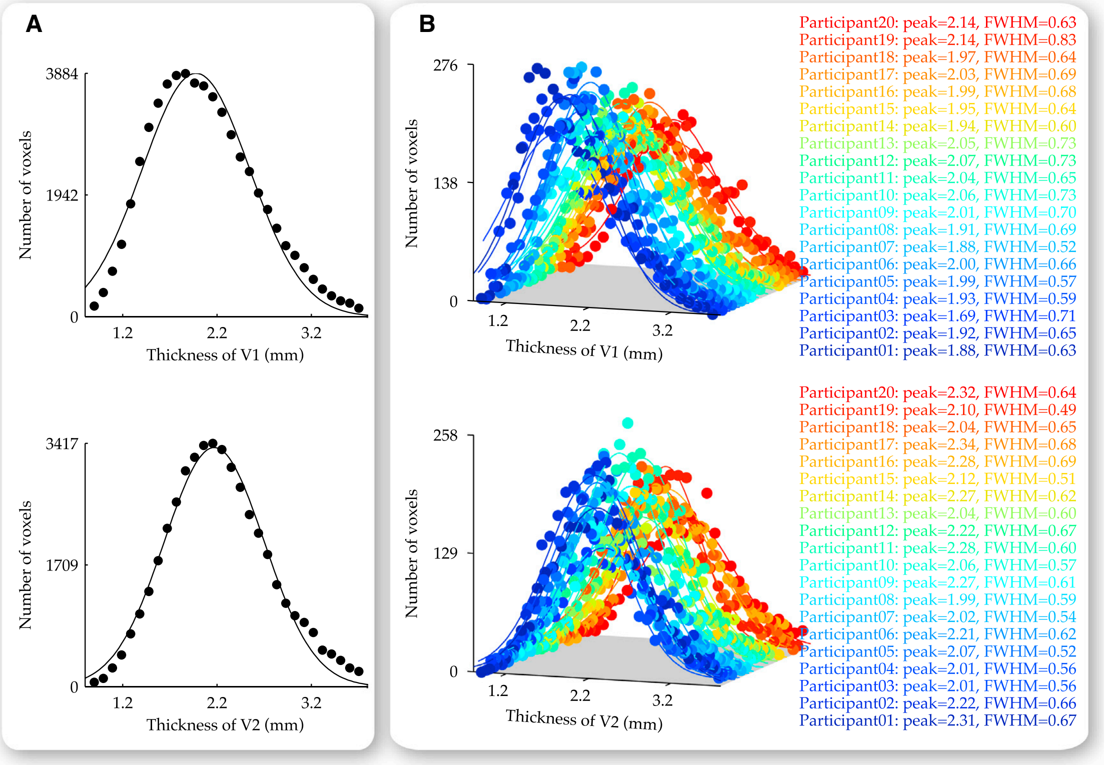

Figure 2. Variability in Visual Cortical Thickness

Based on the retinotopy delineation of early visual cortices (V1 and V2), visual cortical thickness was calculated at individual cortical locations in the retinotopically delineated part of V1 or V2. The distribution of V1 or V2 thickness was plotted on a voxel basis, where voxels with similar thickness were binned to generate 30 data points for the group of 20 participants (A) or for each participant in the group (B). The mean and the SD of V1 or V2 thickness derived from the Gaussian fit to the thickness distribution illustrated the variability in visual cortical thickness across different visual cortical locations. Data points are color coded according to the participant (B). Parameters are derived from the Gaussian fit to the thickness distribution (B).

cortical locations from 1 mm to 4 mm following a Gaussian distribution (Figure 2). In addition to this general intraindividual variability in visual cortical thickness, we observed an intraindividual increase in visual cortical thickness from sulci to gyri and from parafovea (central 2.0 degree eccentricity) to perifovea. This increase in visual cortical thickness from parafovea to perifovea was observed for both sulci (V1, T = 6.533, $p < 0.0001$, n = 20 and V2, T = 8.359, $p < 0.0001$, n = 20) and gyri (V1, T = 6.509, $p < 0.0001$, n = 20 and V2, T = 8.874, $p < 0.0001$, n = 20). In a similar fashion, the increase in visual cortical thickness from sulci to gyri was observed for both parafovea (V1, T = 7.113, $p < 0.0001$, n = 20 and V2, T = 8.357, $p < 0.0001$, n = 20) and perifovea (V1, T = 9.972, $p < 0.0001$, n = 20 and V2, T = 9.471, $p < 0.0001$, n = 20).

While the structural MRI data allowed a noninvasive, in vivo measure of visual cortical anatomy, the measure was, at the same time, limited by its indirect nature. In contrast, a direct measure of cortical anatomy (albeit in vitro) is possible from postmortem histology. Therefore, we further addressed the reliability of our in vivo MRI measure by comparing it with an in vitro histology measure derived from postmortem human brain. Conventional analysis of histology data employs a slice-based approach that is constrained by the slice orientation and has a limited sampling coverage. For example, the slice-based measure of cortical thickness is only valid for histology slices orthogonal to the cortical surface. To overcome this limitation, we developed a surface-based approach and applied it to a data set of high-resolution (40 μm isotropic pixel) whole-brain (4,992 pixel × 3,328 pixel) histology images (502 images in total), taken consecutively every 300 μm along the dorsoventral axis of a postmortem human brain. In the analysis, we manually segmented each histology image into different cortical tissues, from which we reconstructed the 3D cortical surface and acquired the surface-based measure of visual cortical anatomy (Figure S3; Supplemental Experimental Procedures section 3.3). While time-consuming, this surface-based analysis offered a sampling coverage of the full brain that was unconstrained by the slice orientation.

From this histology-based measure of visual cortical anatomy, we observed a substantial degree of intraindividual variability in visual cortical thickness (Figure S4) that was similar in extent to the MRI-based measure (Figure 2). Moreover, the dependence of visual cortical thickness on cortical folding and visual field eccentricity that we observed in the structural MRI data was recaptured by the histology data. Specifically, we observed an increase in visual cortical thickness from sulci to gyri for both parafovea (V1, T = 13.498, $p < 0.0001$, n = 87,455 voxels and

V2, $T = 18.179$, $p < 0.0001$, $n = 86{,}466$ voxels) and perifovea (V1, $T = 23.822$, $p < 0.0001$, $n = 230{,}387$ voxels and V2, $T = 9.507$, $p < 0.0001$, $n = 50{,}348$ voxels), as well as an increase in visual cortical thickness from parafovea to perifovea for both sulci (V1, $T = 83.929$, $p < 0.0001$, $n = 146{,}834$ voxels and V2, $T = 56.089$, $p < 0.0001$, $n = 72{,}410$ voxels) and gyri (V1, $T = 97.783$, $p < 0.0001$, $n = 171{,}008$ voxels and V2, $T = 49.674$, $p < 0.0001$, $n = 64{,}404$ voxels). The consistency with the in vitro histology measure validated our application of in vivo structural MRI data to assess visual cortical anatomy.

Variability in Neural Population Tuning Width

Our measure of neural population tuning for visual field position was based on the established method of population-receptive-field mapping (Dumoulin and Wandell, 2008). In the experiment, a bar stimulus was presented at different visual field positions, and the BOLD time series recorded from each voxel in early visual cortices was deconvolved with a hemodynamic response function before fitting with a 2D Gaussian characterization of position tuning profile (Supplemental Experimental Procedures section 4.1). The 2D Gaussian function characterized the range of visual field positions that the voxel responded to (position tuning width) and the visual field position where the voxel responded the strongest (position tuning peak). We acquired the measure of position tuning for individual visual cortical locations (vertices) from single fMRI voxels, where the exact cortical depth of the voxels did not affect the measure (Figure S5; Supplemental Experimental Procedures section 4.2). This measure of neural population tuning reflected a combined contribution from the average position tuning width of neurons in the voxel and the heterogeneity in position tuning peak between different neurons in the voxel (Hubel and Wiesel, 1974). To improve the resolution of the measure and minimize intravoxel heterogeneity in position tuning peak, we collected the fMRI data at a high spatial resolution (1.5 mm isotropic voxel) using a 3D echo plannar imaging acquisition sequence with parallel imaging acceleration (Lutti et al., 2013). For tissue volumes as small as 1.5 mm isotropic, the heterogeneity in position tuning peak is smaller than the position tuning width of single neurons and is correlated with the average position tuning width of neurons in the tissue volume (Hubel and Wiesel, 1974). As such, the voxel-level measure of the position tuning width in effect reflected the average tuning width of neurons in the voxel. Indeed, this voxel-level position tuning width (0.6 ± 0.35 degree of visual angle) measured here in the retinotopically delineated part of human primary visual cortex was comparable with neural-level position tuning width (0.35 degree of visual angle) in the corresponding part of macaque primary visual cortex (Hubel and Wiesel, 1974).

Despite the improvement in spatial resolution offered by the advanced fMRI acquisition sequence, our measure of the position tuning width was nonetheless still potentially confounded by the temporal lag between neural responses and fMRI signals due to hemodynamic coupling. This potential confounding factor was taken into consideration during the experiment where the BOLD time series was deconvolved with an empirically derived hemodynamic response function before fitting with a 2D Gaussian characterization of position tuning profile. Across voxels, we did not observe significant correlations between the measure of the position tuning width and the parameters of the hemodynamic response function, suggesting that the measure largely reflected the neural response properties rather than the hemodynamic response properties (Supplemental Experimental Procedures section 4.3). In control experiments, we further tested whether the fMRI signal quality might confound our measure of the position tuning width. We found that the fMRI signal-to-noise ratio was rather homogenous across the cortical surface and did not vary systematically with the measure of the position tuning width (Supplemental Experimental Procedures section 4.4). These control studies suggested that our measure of neural population tuning for visual field position was not confounded by intervoxel variability in fMRI signal properties.

From the fMRI-based measure of neural population tuning, we studied variability within and across participants in the position tuning width. Similar to variability in visual cortical thickness, we observed an intraindividual increase in the position tuning width from parafovea to perifovea (V1 sulci, $T = 20.894$, $p < 0.0001$, $n = 20$; V1 gyri, $T = 16.458$, $p < 0.0001$, $n = 20$; V2 sulci, $T = 22.849$, $p < 0.0001$, $n = 20$; and V2 gyri, $T = 20.240$, $p < 0.0001$, $n = 20$) and from sulci to gyri (V1 parafovea, $T = 7.834$, $p < 0.0001$, $n = 20$; V1 perifovea, $T = 5.478$, $p < 0.0001$, $n = 20$; V2 parafovea, $T = 6.675$, $p < 0.0001$, $n = 20$; and V2 perifovea, $T = 6.182$, $p < 0.0001$, $n = 20$), where the slope of this eccentricity-dependent increase varied across participants over 2-fold. In addition to this eccentricity-dependent variability (Dumoulin and Wandell, 2008), we found that the position tuning width also varied intraindividually across different visual field positions at the same eccentricity and the same cortical folding. Moreover, even for the same visual field position, the position tuning width still exhibited a substantial degree of variability across participants. As such, variability in the position tuning width could be decomposed into an eccentricity-dependent and an eccentricity-independent component, respectively.

Variability in Perceptual Discrimination Threshold

The measure of perceptual discrimination for visual field position was based on the standard staircase procedure with a two-alternative forced-choice task. In the experiment, the visual field position difference between two horizontally offset stimuli was varied in a 2-up-1-down staircase to assess the minimum position difference that participants could discriminate. To improve the reliability of this perceptual discrimination measure, we conducted two separate experiments, employing respectively, a spatial forced-choice task where participants discriminated the position difference between two concurrently presented stimuli, and a temporal forced-choice paradigm where participants discriminated the position difference between two sequentially presented stimuli. We found that the measure of the position discrimination threshold from these two different experiments was correlated across participants ($r = 0.652$, $p < 0.01$, $n = 20$), suggesting that the measure reflected a robust, trait-like perceptual variability (Supplemental Experimental Procedures section 5.1).

For each participant, the position discrimination threshold was measured at 13 nonoverlapping visual field positions in 13 independent experiments. The 13 visual field positions covered three eccentricities (0, 4.7, and 6.7 degree) and six polar angles (45,

90, 135, 225, 270, and 315 degree). Such a distributed coverage of the visual field allowed a comprehensive characterization of intraindividual perceptual variability. Specifically, by distributing the 13 visual field positions along both the axis of eccentricity and the axis of polar angle, we separately assessed the eccentricity-dependent and the eccentricity-independent component of intraindividual perceptual variability that reflected respectively, how the position discrimination threshold changed along visual field eccentricity and varied across different visual field positions at the same eccentricity (Supplemental Experimental Procedures section 5.2).

From the psychophysical measure of perceptual discrimination, we studied variability within and across participants in the position discrimination threshold. Consistent with previous reports (Duncan and Boynton, 2003), we observed an intraindividual increase in the position discrimination threshold from parafovea to perifovea, where the slope of this eccentricity-dependent increase varied across participants over 2-fold. When the factor of eccentricity was controlled for, we found that the position discrimination threshold still exhibited a substantial degree of variability. In particular, at a fixed visual field eccentricity, the position discrimination threshold varied across different visual field positions for the same participant and across different participants for the same visual field position. Therefore, similar to variability in the position tuning width, variability in the position discrimination threshold could be decomposed into an eccentricity-dependent and an eccentricity-independent component, respectively.

Dependence of Neural Population Tuning Width and Perceptual Discrimination Threshold on Visual Cortical Anatomy at a Fixed Visual Field Eccentricity

As variability in the position tuning width and position discrimination threshold consisted of both eccentricity-independent and eccentricity-dependent components, we conducted separate analyses to explore the influences that visual cortical anatomy exerted on these two components, respectively. To control for the factor of eccentricity and study the eccentricity-independent component, we analyzed the relationships between visual cortical anatomy, neural population tuning width, and perceptual acuity at a fixed visual field eccentricity (4.7 degree). Across a total of 20 participants and six visual field positions at 4.7 degree eccentricity, we plotted the position tuning width of V1 neural populations and position discrimination threshold of human participants, first against each other to address the behavioral significance of neural population tuning (Figure 3A) and then against thickness or surface area of V1 to address the functional impacts of visual cortical anatomy (Figures 3B and 3C).

We found that the position tuning width of V1 neural populations correlated positively with the position discrimination threshold of our participants (Figure 3A; $r = 0.356$, $p < 0.0001$, $n = 120$). This correlation reflected a combined contribution of intraindividual and interindividual factors. To address the contribution of each factor, we conducted separate analysis where we calculated, for each participant ($n = 20$), the position discrimination threshold as well as the position tuning width averaged across the six visual field positions, and for each visual field position ($n = 6$), the position discrimination threshold as well as the position tuning width averaged across the 20 participants. By subtracting the averages of individual participants, we factored out interindividual variability and studied the contribution of intraindividual factors. Similarly, by subtracting the averages of individual visual field positions, we factored out intraindividual variability and addressed the contribution of interindividual factors. In both cases, we still observed a positive correlation between the position tuning width of V1 neural populations and position discrimination threshold of our participants (intraindividually, $r = 0.350$, $p < 0.0001$, $n = 120$; interindividually, $r = 0.359$, $p < 0.0001$, $n = 120$). This observation illustrated a close correspondence between neural population tuning and human perceptual discrimination.

In addition to the correlation with perceptual discrimination, the position tuning width of V1 neural populations also exhibited correlations with V1 anatomy. Specifically, neural populations in V1 with a larger surface area tended to have a smaller position tuning width (Figure 3B; $r = -0.249$, $p < 0.01$, $n = 120$). In contrast to this negative correlation with V1 surface area, the position tuning width of V1 neural populations exhibited a positive correlation with V1 thickness, where neural populations at V1 locations with a greater thickness tended to have a larger position tuning width (Figure 3B; $r = 0.465$, $p < 0.0001$, $n = 120$). This correlation between the position tuning width and V1 thickness was observed both within individuals ($r = 0.394$, $p < 0.0001$, $n = 120$, interindividual variability factored out), and across individuals ($r = 0.423$, $p < 0.0001$, $n = 120$, intraindividual variability factored out). These findings suggested that the two anatomical dimensions, thickness and surface area, of V1 had opposite impacts on neural population tuning for visual field position.

The functional impacts of visual cortical anatomy on neural population tuning were further reflected in perceptual discrimination. We found that thickness and surface area of V1 both exhibited correlations with the position discrimination threshold of our participants. Specifically, participants with a larger V1 surface area tended to have a smaller position discrimination threshold (Figure 3C; $r = -0.318$, $p < 0.001$, $n = 120$). In contrast to this negative correlation between the position discrimination threshold and V1 surface area, a positive correlation was observed between the position discrimination threshold at different visual field positions and V1 thickness at corresponding cortical locations (Figure 3C; $r = 0.307$, $p < 0.001$, $n = 120$), within individuals ($r = 0.339$, $p < 0.001$, $n = 120$, interindividual variability factored out) as well as across individuals ($r = 0.311$, $p < 0.001$, $n = 120$, intraindividual variability factored out).

These observations revealed that thickness and surface area of human V1 had influences on both neural population tuning and perceptual discrimination. To explore the influences that V2 anatomy might exert on the position tuning width of V2 neural populations and the position discrimination threshold of our participants, we applied a similar analysis. We plotted the position tuning width of V2 neural populations and the position discrimination threshold of our participants, first against each other and then against thickness or surface area of V2, for a total of 20 participants and six visual field positions at 4.7 degree eccentricity. Similar to our observations in V1, the position tuning width of V2 neural populations correlated positively with the position discrimination threshold of our participants (Figure 4A; $r = 0.252$, $p < 0.01$, $n = 120$; intraindividually, $r = 0.230$, $p < 0.05$,

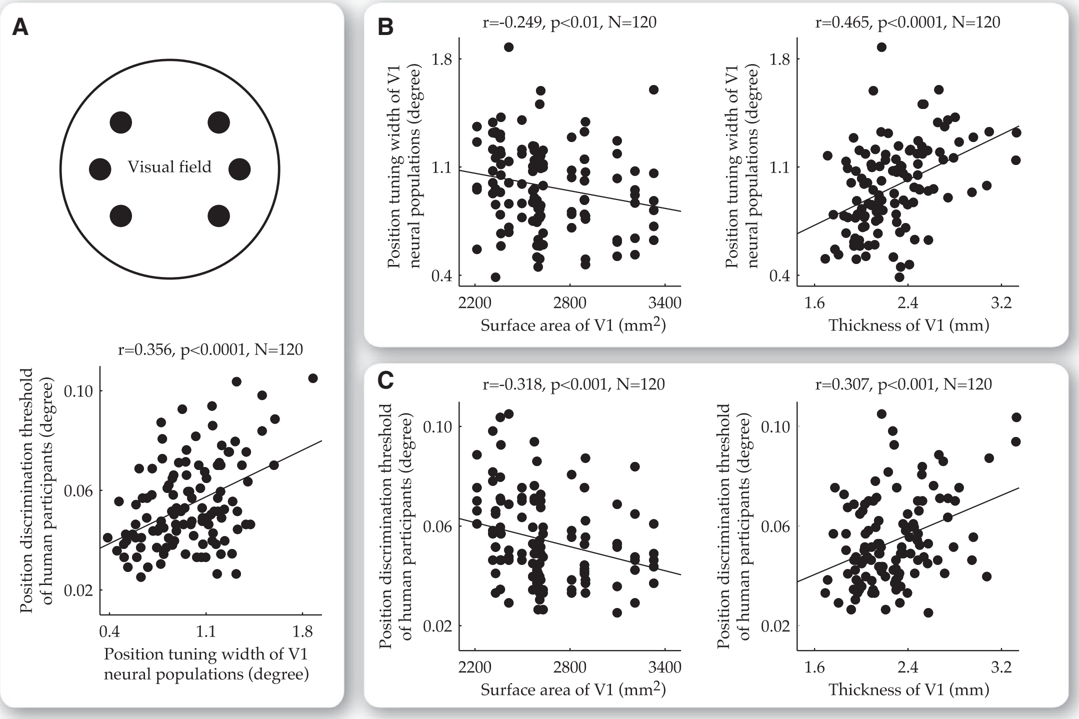

Figure 3. Dependence of Neural Population Tuning Width and Perceptual Discrimination Threshold on V1 Anatomy at a Fixed Visual Field Eccentricity

Across a total of 20 participants and six visual field positions at 4.7 degree eccentricity, the position tuning width of V1 neural populations (measured using fMRI) and the position discrimination threshold of human participants (measured using psychophysics) were plotted against each other (A) and against V1 surface area or V1 thickness (B and C). These analyses revealed a positive correlation between the position discrimination threshold of our participants and the position tuning width of V1 neural populations (A), as well as a dependence of both the position tuning width (B) and position discrimination threshold (C) on V1 anatomy. Specifically, the position tuning width of V1 neural populations (B), and the position discrimination threshold of our participants (C), correlated positively with V1 surface area, but negatively with V1 thickness. A further analysis, where interindividual variability and intraindividual variability were regressed out respectively, revealed that these correlations between neural population tuning width, perceptual acuity, and V1 anatomy existed both within and across individuals. Each data point represents the measures at a single visual field position from a single participant. Statistical values reflect permutation-based Spearman's rank correlation with familywise error (FWE) correction for multiple comparisons.

$n = 120$; and interindividually, $r = 0.261$, $p < 0.01$, $n = 120$). Moreover, both the position tuning width and position discrimination threshold exhibited correlations with V2 anatomy. Specifically, participants with a larger V2 surface area tended to have a smaller position tuning width (Figure 4B; $r = -0.295$, $p < 0.01$, $n = 120$) and a smaller position discrimination threshold (Figure 4C; $r = -0.315$, $p < 0.001$, $n = 120$). In contrast, a larger position tuning width (Figure 4B; $r = 0.322$, $p < 0.001$, $n = 120$; intraindividually, $r = 0.366$, $p < 0.0001$, $n = 120$; and interindividually, $r = 0.276$, $p < 0.01$, $n = 120$) and a larger position discrimination threshold (Figure 4C; $r = 0.205$, $p < 0.05$, $n = 120$; intraindividually, $r = 0.200$, $p < 0.05$, $n = 120$; and interindividually, $r = 0.193$, $p < 0.05$, $n = 120$) were observed at the visual field positions that corresponded to V2 locations with a larger thickness.

Dependence of Neural Population Tuning Width and Perceptual Discrimination Threshold on Visual Cortical Anatomy along Visual Field Eccentricity

Our analyses at a fixed visual field eccentricity (4.7 degree) suggested that thickness and surface area of human early visual cortices (V1 and V2) had opposite impacts on neural population tuning that in turn affected perceptual discrimination. To study whether these observations were specific to certain eccentricity or were generalizable across the visual field, we conducted further analyses to address the impacts that visual cortical anatomy had on eccentricity-dependent variability in neural population tuning width and perceptual discrimination threshold. Specifically, by fitting the position tuning width and position discrimination threshold as a function of visual field eccentricity, we explored how visual cortical anatomy might relate to the slope and the intercept of the fit. A relationship with the slope of the fit would indicate that the position tuning width and position discrimination threshold got more dependent on visual cortical anatomy as one approached the peripheral visual field, whereas a relationship with the intercept of the fit would indicate an increased dependence toward the central visual field.

Across the group of 20 participants and the retinotopically delineated coverage of the visual field (0.25–7.2 degree eccentricity), we plotted the position tuning width at individual V1 locations (vertices) against visual field eccentricities these locations

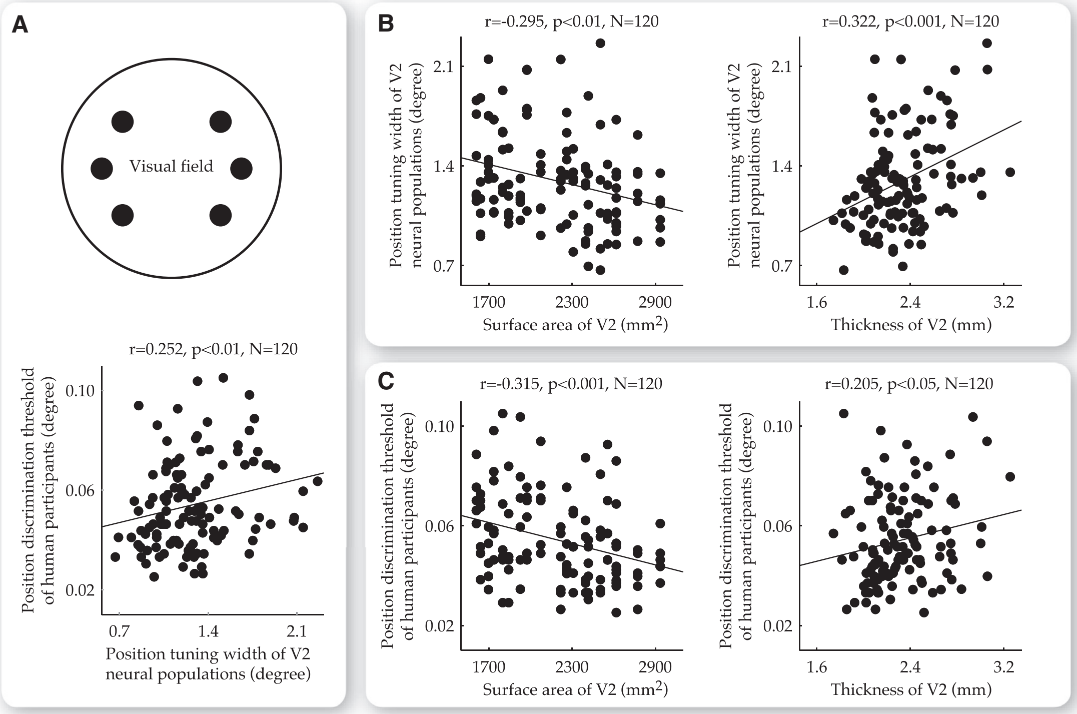

Figure 4. Dependence of Neural Population Tuning Width and Perceptual Discrimination Threshold on V2 Anatomy at a Fixed Visual Field Eccentricity

Across a total of 20 participants and six visual field positions at 4.7 degree eccentricity, the position tuning width of V2 neural populations (measured using fMRI) and the position discrimination threshold of human participants (measured using psychophysics) were plotted against each other (A) and against V2 surface area or V2 thickness (B and C). These analyses revealed a positive correlation between the position discrimination threshold of our participants and the position tuning width of V2 neural populations (A), as well as a dependence of both the position tuning width (B), and position discrimination threshold (C) on V2 anatomy. Specifically, the position tuning width of V2 neural populations (B), and the position discrimination threshold of our participants (C), correlated positively with V2 surface area, but negatively with V2 thickness. A further analysis, where interindividual variability and intraindividual variability were regressed out respectively, revealed that these correlations between neural population tuning width, perceptual acuity, and V2 anatomy existed both within and across individuals. Each data point represents the measures at a single visual field position from a single participant. Statistical values reflect permutation-based Spearman's rank correlation with FWE correction for multiple comparisons.

responded to and V1 surface area of the participants. The data were binned into a data grid where individual data points represented the position tuning width averaged over V1 locations (vertices) that were from the same participant and responded to similar eccentricities (Figure 5A). This 3D data grid allowed us to separately address the influences that visual field eccentricity and V1 surface area exerted on the position tuning width of V1 neural populations. Along the axis of V1 surface area, we fitted individual plots of the position tuning width, visual field eccentricity with linear regression functions, where each plot represented the data from a single participant (Figure 5B). We found that the slope ($r = -0.549$, $p < 0.05$, $n = 20$) and the intercept ($r = -0.614$, $p < 0.01$, $n = 20$) of the linear fit both correlated negatively with surface area of V1, while the goodness of the fit did not exhibit such correlation ($r = 0.272$, $p = 0.245$, $n = 20$). Therefore, neural populations in V1 with a larger surface area tended to have a smaller position tuning width near fovea, as well as a slower position tuning width increase along visual field eccentricity. These results suggested that the dependence of the position tuning width on V1 surface area was likely to be a general observation that spanned the visual field. Indeed, when correlation analysis was applied along the axis of visual field eccentricity directly on individual plots of the position tuning width, V1 surface area (Figure 5C), we observed negative correlations within individual ranges of visual field eccentricity between the position tuning width and V1 surface area.

Thus, the functional impacts of V1 surface area on neural population tuning width, which we observed at 4.7 degree eccentricity, were generalizable across the visual field. To study the functional impacts of V1 thickness, we applied a similar analytic approach. Across the group of 20 participants and the retinotopically delineated coverage of visual field (0.25−7.2 degree eccentricity), we plotted the position tuning width at individual V1 locations (vertices) against visual field eccentricities that these locations responded to and V1 thickness at these locations. The data were binned into a data grid where individual data points represented the position tuning width averaged over V1 locations (vertices) that were similar in thickness and responded to similar eccentricities (Figure 5A). Through this 3D data grid, we separately addressed the influences that visual field eccentricity

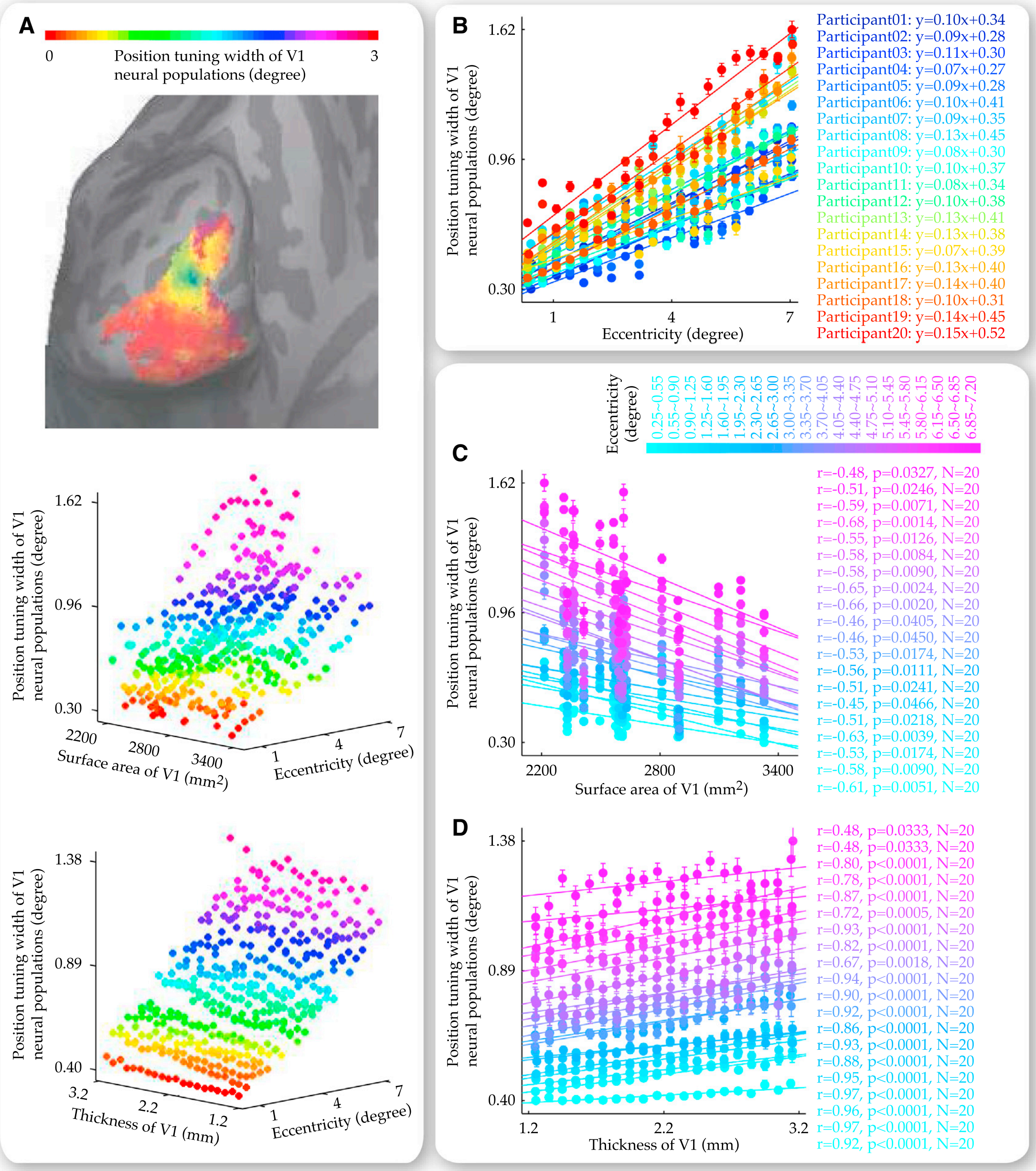

Figure 5. Relationship between Neural Population Tuning Width and V1 Anatomy along Visual Field Eccentricity

The cortical surface map from a representative participant illustrated the width of neural population tuning at individual V1 cortical surface locations (vertices) for corresponding visual field positions (A). Based on the cortical surface maps from all 20 participants, we plotted the position tuning width at individual V1 locations against visual field eccentricities these locations responded to and V1 anatomy at these locations. The 3D plots were binned into data grids where individual data points represented the position tuning width averaged over V1 locations that responded to similar eccentricities and were from the same participant or had similar thickness (A). The data grids allowed us to disentangle the influences that visual field eccentricity (B) and V1 anatomy (C and D) exerted on the position tuning width of V1 neural populations. Specifically, along the axis of V1 surface area, each plot of the position tuning width, visual field eccentricity represented the data from a single participant and illustrated the increase in the position tuning width with visual field eccentricity (B). Along the axis of visual field eccentricity, each plot of the position tuning width, V1 anatomy represented the data from a single eccentricity range and illustrated the dependence of the position tuning width on V1 surface area (C) or V1 thickness (D). Data points are color coded according to the position tuning width (A), the participant (B), or the visual field eccentricity (C and D). Equations (B) reflect linear fit to the plot of the position tuning width, visual field eccentricity. Statistical values (C and D) reflected permutation-based Spearman's rank correlation with FWE correction for multiple comparisons. Error bars represent 1 SEM.

and V1 thickness exerted on the position tuning width of V1 neural populations. Along the axis of V1 thickness, we fitted individual plots of the position tuning width, visual field eccentricity with linear regression functions, where each plot represented the data from a single thickness range of 0.1 mm. We found that the slope ($r = 0.528$, $p < 0.05$, $n = 20$) and the intercept ($r = 0.935$, $p < 0.0001$, $n = 20$) of the linear fit both correlated positively with thickness of V1, while the goodness of the fit did not covary with V1 thickness ($r = -0.147$, $p = 0.534$, $n = 20$). The influences of V1 thickness on both the slope and the intercept of the fit suggested that the dependence of position tuning width on V1 thickness was not specific to certain eccentricity ranges, but was instead generalizable across the visual field. To verify this, we applied correlation analysis directly to individual plots of the position tuning width, V1 thickness, along the axis of visual field eccentricity (Figure 5D). We observed positive correlations between the position tuning width and V1 thickness, within individual ranges of visual field eccentricity.

These analyses suggested that, across the visual field, neural population tuning in V1 for a specific visual field position was affected jointly by thickness at corresponding V1 locations and surface area of V1. We further investigated whether such influences on neural population tuning would have behavioral consequences on perceptual discrimination. The threshold of perceptual discrimination, measured at 13 nonoverlapping visual field positions covering three eccentricities (0, 4.7, and 6.7 degree) and six polar angles (45, 90, 135, 225, 270, and 315 degree), was projected onto V1 to generate a cortical map of perceptual acuity. This cortical projection allowed us to relate perceptual acuity at different visual field positions with V1 anatomy at corresponding cortical locations, using analyses similar to the ones on neural population tuning width.

On a vertex-by-vertex level, we plotted the position discrimination threshold at individual V1 locations (vertices) against visual field eccentricities that these locations responded to and V1 thickness at these locations or V1 surface area of the participants (Figure 6A). The data were binned into data grids characterizing the increase in the position discrimination threshold along visual field eccentricity, as well as the relationships between the position discrimination threshold and V1 anatomy within individual ranges of visual field eccentricity. Mirroring the observations on neural population tuning width, we found that the functional impacts of V1 surface area and V1 thickness on perceptual acuity, which we observed at 4.7 degree eccentricity, were generalizable across the visual field. Specifically, the position discrimination threshold correlated negatively with V1 surface area (Figure 6C), in a fashion that participants with a larger V1 surface area had not only a smaller position discrimination threshold in the fovea ($-r = 0.675$, $p < 0.01$, $n = 20$), but also a slower position discrimination threshold increase along visual field eccentricity ($-r = 0.532$, $p < 0.05$, $n = 20$). In contrast, positive correlations were observed within individual ranges of visual field eccentricity between the position discrimination threshold and V1 thickness (Figure 6D).

Together, these analyses suggested that our observations at 4.7 degree eccentricity, where thickness and surface area of V1 exerted opposite influences on the position tuning width of V1 neural populations with behavioral consequences on the position discrimination threshold of human participants, were generalizable across the visual field. To investigate whether such generalization was also observable in V2, we plotted the position tuning width (Figure 7) and position discrimination threshold (Figure 8) at individual V2 locations (vertices) against visual field eccentricities these locations responded to and V2 anatomy at these locations. Similar to our observations in V1, surface area of V2 correlated negatively with the position tuning width of V2 neural populations (Figure 7C) and the position discrimination threshold of our participants (Figure 8C), and specifically, with their value near the fovea (tuning width, $r = -0.729$, $p < 0.001$, $n = 20$ and discrimination threshold, $r = -0.596$, $p < 0.01$, $n = 20$), as well as their slope of increase along visual field eccentricity (tuning width, $r = -0.705$, $p < 0.001$, $n = 20$ and discrimination threshold, $r = -0.642$, $p < 0.01$, $n = 20$). In contrast, thickness of V2 exhibited positive correlations with the position tuning width (Figure 7D) and position discrimination threshold (Figure 8D), within individual ranges of visual field eccentricity.

DISCUSSION

It is intuitive to assume that a larger cortical volume has some behavioral advantage. Indeed, within species, a positive correlation is usually observed between performance on behavioral tasks and local cortical volume in task-relevant cortical regions (Kanai and Rees, 2011). However, the fundamental questions of whether a larger cortical volume is indeed the critical factor and why cortical volume is even relevant to understanding behavioral performance remain unaddressed. Here, we suggest two possible mechanisms. It is plausible that the influences of cortical anatomy on behavioral performance are mediated simply by the volume of cortical tissue available for information processing and, correspondingly, the signal-to-noise ratio during information processing. Alternatively, the neural response properties that are associated with cortical anatomy may underlie its influences on behavioral performance. To disentangle these two hypotheses, we separately studied the two anatomical dimensions, thickness and surface area, of cerebral cortex. These two elementary dimensions both contribute to cortical volume, yet characterize distinct aspects of volumetric changes (local versus global) that may differently affect the neural response properties. This allowed us to address whether it is cortical volumes per se or the associated neural response properties that link cortical anatomy to behavioral performance.

We used early visual cortices as a model system, since the orderly representation of visual field position in early visual cortices allowed fMRI-based, noninvasive characterization of neural population tuning (Dumoulin and Wandell, 2008; Fischer and Whitney, 2009). Utilizing this fMRI-based measure of neural population tuning, we investigated how the anatomy of human early visual cortices influenced the population tuning properties of visual cortical neurons and whether such influences were behaviorally significant. We found that neural population tuning and perceptual discrimination were finer in individuals with a larger surface area of early visual cortices. Therefore, a larger visual cortical volume, if it came from a larger visual cortical surface area, was associated with a better performance in

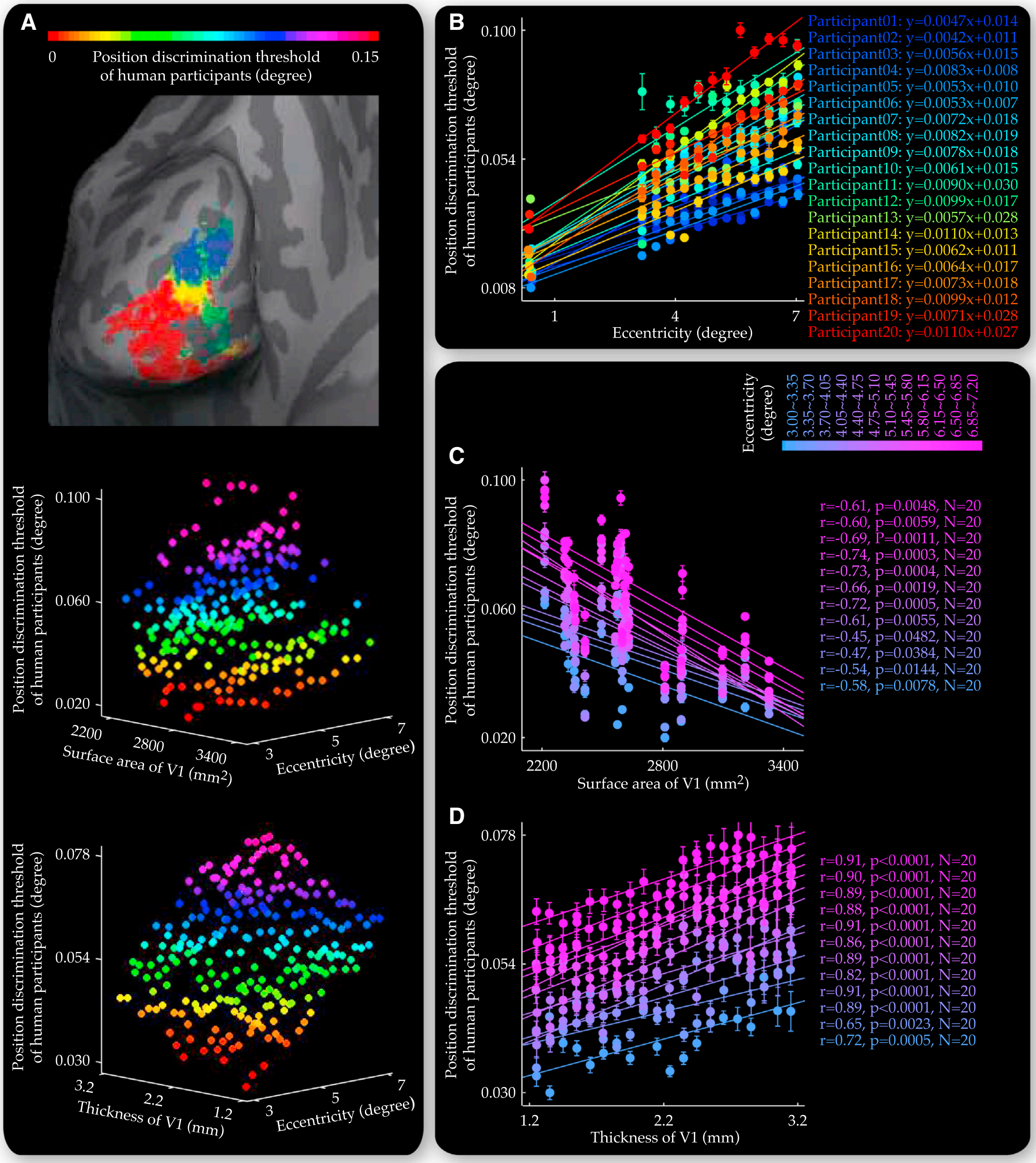

Figure 6. Relationship between Perceptual Discrimination Threshold and V1 Anatomy along Visual Field Eccentricity

The threshold of perceptual discrimination, measured at 13 nonoverlapping visual field positions covering three eccentricities (0, 4.7, and 6.7 degree) and six polar angles (45, 90, 135, 225, 270, and 315 degree), was projected onto V1 to generate a cortical surface map for each participant that illustrated variability across different V1 cortical surface locations (vertices) in perceptual discrimination threshold for corresponding visual field positions (A). Based on the cortical surface maps from all 20 participants, we plotted the position discrimination threshold at individual V1 locations against visual field eccentricities these locations responded to and V1 anatomy at these locations. The 3D plots were binned into data grids where individual data points represented the position discrimination threshold averaged over V1 locations that responded to similar eccentricities and were from the same participant or had similar thickness (A). For each participant, different V1 locations that were projected with the measure of position discrimination threshold at the central visual field (zero eccentricity) were binned into a single data point. The data grids allowed us to disentangle the influences that visual field eccentricity (B) and V1 anatomy (C and D) exerted on the position discrimination threshold. Specifically, along the axis of V1 surface area, each plot of the position discrimination threshold, visual field eccentricity represented the data from a single participant and illustrated the increase in the position discrimination threshold with visual field eccentricity (B). Along the axis of visual field eccentricity, each plot of the position discrimination threshold, V1 anatomy represented the data from a single eccentricity range and illustrated the dependence of the position discrimination threshold on V1 surface area (C) or V1 thickness (D). Data points are color coded according to the position discrimination threshold (A), the participant (B), or the visual field eccentricity (C and D). Equations (B) reflect linear fit to the plot of the position discrimination threshold, visual field eccentricity. Statistical values (C and D) reflected permutation-based Spearman's rank correlation with FWE correction for multiple comparisons. Error bars represent 1 SEM.

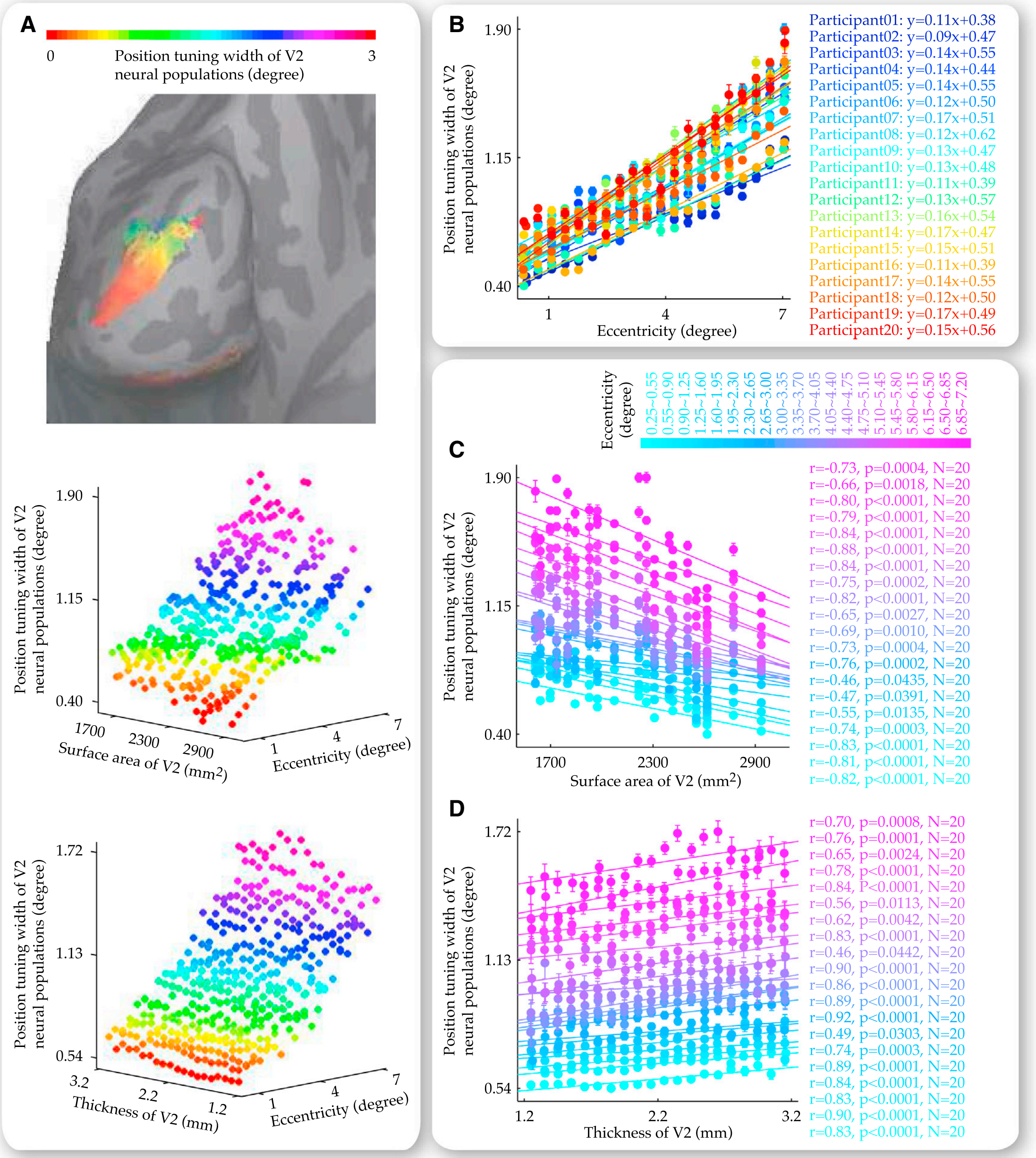

Figure 7. Relationship between Neural Population Tuning Width and V2 Anatomy along Visual Field Eccentricity

The cortical surface map from a representative participant illustrated the width of neural population tuning at individual V2 cortical surface locations (vertices) for corresponding visual field positions (A). Based on the cortical surface maps from all 20 participants, we plotted the position tuning width at individual V2 locations against visual field eccentricities these locations responded to and V2 anatomy at these locations. The 3D plots were binned into data grids where individual data points represented the position tuning width averaged over V2 locations that responded to similar eccentricities and were from the same participant or had similar thickness (A). The data grids allowed us to disentangle the influences that visual field eccentricity (B) and V2 anatomy (C and D) exerted on the position tuning width of V2 neural populations. Specifically, along the axis of V2 surface area, each plot of the position tuning width, visual field eccentricity represented the data from a single participant and illustrated the increase in the position tuning width with visual field eccentricity (B). Along the axis of visual field eccentricity, each plot of the position tuning width, V2 anatomy represented the data from a single eccentricity range and illustrated the dependence of the position tuning width on V2 surface area (C) or V2 thickness (D). Data points are color coded according to the position tuning width (A), the participant (B), or the visual field eccentricity (C and D). Equations (B) reflect linear fit to the plot of the position tuning width, visual field eccentricity. Statistical values (C and D) reflected permutation-based Spearman's rank correlation with FWE correction for multiple comparisons. Error bars represent 1 SEM.

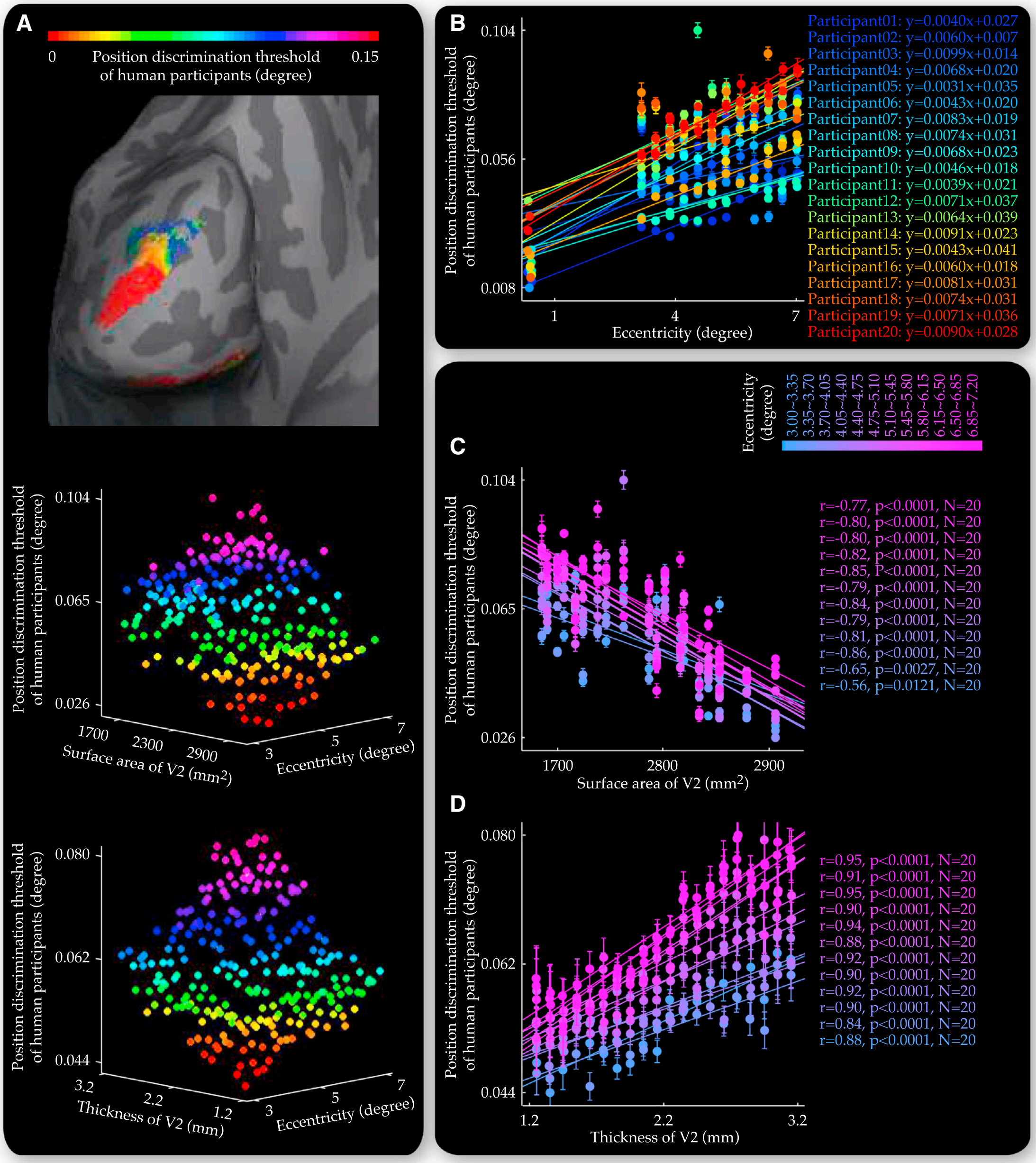

Figure 8. Relationship between Perceptual Discrimination Threshold and V2 Anatomy along Visual Field Eccentricity

The threshold of perceptual discrimination, measured at 13 nonoverlapping visual field positions covering three eccentricities (0, 4.7, and 6.7 degree) and six polar angles (45, 90, 135, 225, 270, and 315 degree), was projected onto V2 to generate a cortical surface map for each participant that illustrated variability across different V2 cortical surface locations (vertices) in perceptual discrimination threshold for corresponding visual field positions (A). Based on the cortical surface maps from all 20 participants, we plotted the position discrimination threshold at individual V2 locations against visual field eccentricities these locations responded to and V2 anatomy at these locations. The 3D plots were binned into data grids where individual data points represented the position discrimination threshold averaged over V2 locations that responded to similar eccentricities and were from the same participant or had similar thickness (A). For each participant, different V2 locations that were projected with the measure of the position discrimination threshold at the central visual field (zero eccentricity) were binned into a single data point. The data grids allowed us to disentangle the influences that visual field eccentricity (B) and V2 anatomy (C and D) exerted on the position discrimination threshold. Specifically, along the axis of V2 surface area, each plot of the position discrimination threshold, visual field eccentricity represented the data from a single participant and illustrated the increase in the position discrimination threshold with visual field eccentricity (B). Along the axis of visual field eccentricity, each plot of the position discrimination threshold, V2 anatomy represented the data from a single eccentricity range and illustrated the dependence of the position discrimination threshold on V2 surface area (C) or V2 thickness (D). Data points are color coded according to the position discrimination threshold (A), the participant (B), or the visual field eccentricity (C and D). Equations (B) reflect linear fit to the plot of the position discrimination threshold, visual field eccentricity. Statistical values (C and D) reflected permutation-based Spearman's rank correlation with FWE correction for multiple comparisons. Error bars represent 1 SEM.

perceptual discrimination and a higher selectivity in neural population tuning. Intriguingly, the exact opposite impacts were observed for visual cortical thickness, where neural population tuning and perceptual discrimination were finer for visual field positions corresponding to thinner parts of early visual cortices. As such, a larger visual cortical volume, if it came from a larger visual cortical thickness, was associated with a poorer performance in perceptual discrimination and a lower selectivity in neural population tuning.

Our findings suggested a larger visual cortical volume is not in itself advantageous for visual perception. Instead, a perceptually advantageous cortical design may involve a thinned visual cortex with an enlarged surface area. This is consistent with the developmental trend that sensory experience drives the expansion of sensory cortical maps, but thinning of sensory cortex (Gilbert et al., 2001; Jiang et al., 2009). Moreover, the association between a thinner visual cortex and a finer visual function is consistent with a similar trend in the retina. In the retina, the part with the highest acuity, the fovea, is also the thinnest. The fovea has only one photoreceptor layer that potentially minimizes the absorption of light signal along the retinal pathway (Jacobson et al., 2007). As such, a finer visual function may in general be achieved not through a simple increase in tissue volume, but instead through the optimization of tissue distribution. Indeed, a thinned visual cortex with an enlarged surface area is likely to optimize the selectivity of visual cortical neurons by maximizing the number of intercolumnar processing units and minimizing the delay of interlaminar processing (Bugbee and Goldman-Rakic, 1983; Rakic, 1988; Mountcastle, 1997; Jones, 2000; Kaas, 2000).

Specifically, thickening of visual cortex is likely to burden intracortical processing, as the axons and the dendrites of interlaminar connections would need to double and quadruple in diameter to improve interlaminar conduction speed in order to maintain the same interlaminar processing time (delay) (Kaas, 2000). Due to the physical constrains on wiring costs, interlaminar connections tend to fall behind the increase in cortical thickness, leading to increased interlaminar processing time (Ringo, 1991; Kaas, 2000; Angelucci et al., 2002; Sporns and Zwi, 2004; Lewis et al., 2009). Such an increase in interlaminar processing time would facilitate response synchronization among different cortical columns, and in turn, decrease the functional specificity (selectivity) of individual cortical columns (Koch, 1984; Ringo, 1991; Kaas, 2000; Womelsdorf et al., 2007; Sun and Dan, 2009). Therefore, a larger visual cortical thickness is likely to be associated with a lower selectivity in neural tuning, and correspondingly, a poorer performance in perceptual discrimination. By contrast, the enlargement of visual cortical surface area is likely to benefit intracortical processing through an increase in the number of cortical columns available for intercolumnar processing. This increased number of cortical columns, at the same time, would be accompanied by a proportionally decreased connectivity between different cortical columns, as the absolute length of intercolumnar connections is physically constrained and remains independent of visual cortical size (Bugbee and Goldman-Rakic, 1983; Rakic, 1988; Ringo, 1991; Mountcastle, 1997; Jones, 2000; Kaas, 2000). Such a decrease in the proportion of cortical columns with which an individual cortical column connects would in turn increase the functional specificity (selectivity) of individual cortical columns. Therefore, a larger visual cortical surface area is likely to be associated with a higher selectivity in neural tuning, and correspondingly, a better performance in perceptual discrimination.

Limited by the current resolution of noninvasive neuroimaging techniques, an empirical assessment of intracortical processing in human participants is not easy. Nevertheless, it might be of interest for future studies to explore whether a visual cortical model that incorporated these hypothetical changes in intracortical processing could reproduce our empirically observed correlations between visual cortical anatomy and neural population tuning. Regardless of the underlying mechanisms, our findings revealed that the population tuning properties of visual cortical neurons play an important role in linking visual cortical anatomy to visual perception. We showed that it is not cortical volume per se, but rather the associated neural response properties that mediate the influences of cortical anatomy on behavioral performance. This raises concerns for the classical approach taken in studying the anatomical basis of behavioral performance, where one simply searches for cortical regions whose local volume correlates positively with behavioral performance (Kanai and Rees, 2011). By demonstrating that the two determinants of cortical volume, cortical thickness and cortical surface area, may have opposite functional impacts (at least for visual perception), our findings call for a more nuanced approach to be taken in future research, where the effects of variability in cortical thickness and cortical surface area are examined independently, and any negative correlation between cortical volume and behavioral performance is not overlooked. Moreover, by showing (albeit implicitly) that a thinned visual cortex with an enlarged surface area is perceptually advantageous, our findings suggested a future research direction where one may explicitly study what constitutes a behaviorally advantageous cortical design.

EXPERIMENTAL PROCEDURES

In a group of 20 healthy human adults, we studied the relationships among the anatomy of early visual cortices (V1 and V2), the width of neural population tuning, and the threshold of perceptual discrimination, measured respectively using structural imaging, fMRI, and visual psychophysics. First, we acquired the measure of visual cortical anatomy by applying the surface-based analysis to early visual cortices delineated on the structural imaging data. To improve the reliability of the measure, we used different experimental paradigms, where we delineated early visual cortices retinotopically according to the phase-encoded map (Sereno et al., 1995), retinotopically according to the population-receptive-field map (Dumoulin and Wandell, 2008), or morphologically according to the cortical folding patterns (Desikan et al., 2006), acquired the structural data from in vivo T1-weighted MRI imaging, in vivo quantitative-T1 MRI imaging, or in vitro histology sectioning, and conducted the surface-based analysis in SPM (Ashburner, 2012), FSL (Jenkinson et al., 2012), Freesurfer (Fischl, 2012), or MPAV CBS (Bazin et al., 2013). Then, we measured neural population tuning for visual field position using the method of population-receptive-field mapping (Dumoulin and Wandell, 2008), where a bar stimulus was presented at 64 different visual field positions, and the fMRI BOLD time series recorded from each voxel in early visual cortices was fitted with a 2D Gaussian function quantifying the position tuning width and position tuning peak. We took into consideration the potential confounding factor of fMRI signal properties by conducting control experiments addressing the influences of fMRI spatial sampling, fMRI hemodynamic coupling, and fMRI signal-to-noise ratio on the measure of neural population tuning. Finally, we assessed the threshold

for perceptual discrimination of visual field position, based on the psychophysical staircase procedure with a forced-choice task. To test whether the measure represented a perceptual trait robust against the experimental paradigm, we performed separate experiments employing respectively, a spatial forced-choice task where participants discriminated the visual field position difference between two concurrently presented stimuli, and a temporal forced-choice paradigm where participants discriminated the visual field position difference between two sequentially presented stimuli. For each participant, the threshold of perceptual discrimination, measured at 13 nonoverlapping visual field positions covering three eccentricities (0, 4.7, and 6.7 degree) and six polar angles (45, 90, 135, 225, 270, and 315 degree), was projected onto early visual cortices to generate a personalized cortical map of perceptual acuity. Together these measures allowed us to relate, on a voxel basis, the anatomy at different visual cortical locations with the width of neural population tuning and the threshold of perceptual discrimination for corresponding visual field positions. The experiment details are described in Supplemental Experimental Procedures.

SUPPLEMENTAL INFORMATION

Supplemental Information includes Supplemental Experimental Procedures and five figures and can be found with this article online at http://dx.doi.org/10.1016/j.neuron.2014.12.041.

ACKNOWLEDGMENTS

We thank Antoine Lutti and Nikolaus Weiskopf for help with collecting and analyzing quantitative-T1 MRI data; Yuchun Tang and Shuwei Liu for sharing histology data; Xiaocheng Sun, Junhai Xu, Yuchun Tang, and Bo Sun for help with preprocessing and segmenting histology data; Pierre-Louis Bazin, Christine Tardif, and Robert Turner for help with cortical surface reconstruction of histology data; and Karl Zilles, Katrin Amunts, and Marty Sereno for general discussions on histology data analysis. This work was supported by the Brain Research Trust (C.S.), the European Research Council (D.S.S.), the Japan Society for the Promotion of Science (R.K.), and the Wellcome Trust (G.R.).

Accepted: December 9, 2014
Published: January 22, 2015

REFERENCES

Angelucci, A., Levitt, J.B., Walton, E.J., Hupe, J.M., Bullier, J., and Lund, J.S. (2002). Circuits for local and global signal integration in primary visual cortex. J. Neurosci. *22*, 8633–8646.

Ashburner, J. (2012). SPM: a history. Neuroimage *62*, 791–800.

Bazin, P.L., Weiss, M., Dinse, J., Schäfer, A., Trampel, R., and Turner, R. (2013). A computational framework for ultra-high resolution cortical segmentation at 7 Tesla. Neuroimage. Published online April 25, 2013. http://dx.doi.org/10.1016/j.neuroimage.2013.03.077.

Blinkov, S.M., and Glezer, I.J. (1968). The Human Brain in Figures and Tables: A Quantitative Handbook. (New York: Plenum Press).

Bugbee, N.M., and Goldman-Rakic, P.S. (1983). Columnar organization of corticocortical projections in squirrel and rhesus monkeys: similarity of column width in species differing in cortical volume. J. Comp. Neurol. *220*, 355–364.

Chen, C.H., Panizzon, M.S., Eyler, L.T., Jernigan, T.L., Thompson, W., Fennema-Notestine, C., Jak, A.J., Neale, M.C., Franz, C.E., Hamza, S., et al. (2011). Genetic influences on cortical regionalization in the human brain. Neuron *72*, 537–544.

Dale, A.M., Fischl, B., and Sereno, M.I. (1999). Cortical surface-based analysis. I. Segmentation and surface reconstruction. Neuroimage *9*, 179–194.

Desikan, R.S., Ségonne, F., Fischl, B., Quinn, B.T., Dickerson, B.C., Blacker, D., Buckner, R.L., Dale, A.M., Maguire, R.P., Hyman, B.T., et al. (2006). An automated labeling system for subdividing the human cerebral cortex on MRI scans into gyral based regions of interest. Neuroimage *31*, 968–980.

Dougherty, R.F., Koch, V.M., Brewer, A.A., Fischer, B., Modersitzki, J., and Wandell, B.A. (2003). Visual field representations and locations of visual areas V1/2/3 in human visual cortex. J. Vis. *3*, 586–598.

Dumoulin, S.O., and Wandell, B.A. (2008). Population receptive field estimates in human visual cortex. Neuroimage *39*, 647–660.

Duncan, R.O., and Boynton, G.M. (2003). Cortical magnification within human primary visual cortex correlates with acuity thresholds. Neuron *38*, 659–671.

Fischer, J., and Whitney, D. (2009). Attention narrows position tuning of population responses in V1. Curr. Biol. *19*, 1356–1361.

Fischl, B. (2012). FreeSurfer. Neuroimage *62*, 774–781.

Fischl, B., and Dale, A.M. (2000). Measuring the thickness of the human cerebral cortex from magnetic resonance images. Proc. Natl. Acad. Sci. USA *97*, 11050–11055.

Gilbert, C.D., Sigman, M., and Crist, R.E. (2001). The neural basis of perceptual learning. Neuron *31*, 681–697.

Haug, H. (1987). Brain sizes, surfaces, and neural sizes of the cerebral cortex: A stereological investigation of man and his variability and a comparison with some mammals. Am. J. Anat. *180*, 126–142.

Hilgetag, C.C., and Barbas, H. (2006). Role of mechanical factors in the morphology of the primate cerebral cortex. PLoS Comput. Biol. *2*, e22.

Hubel, D.H., and Wiesel, T.N. (1974). Uniformity of monkey striate cortex: a parallel relationship between field size, scatter, and magnification factor. J. Comp. Neurol. *158*, 295–305.

Jacobson, S.G., Aleman, T.S., Cideciyan, A.V., Heon, E., Golczak, M., Beltran, W.A., Sumaroka, A., Schwartz, S.B., Roman, A.J., Windsor, E.A., et al. (2007). Human cone photoreceptor dependence on RPE65 isomerase. Proc. Natl. Acad. Sci. USA *104*, 15123–15128.

Jenkinson, M., Beckmann, C.F., Behrens, T.E., Woolrich, M.W., and Smith, S.M. (2012). FSL. Neuroimage *62*, 782–790.

Jiang, J., Zhu, W., Shi, F., Liu, Y., Li, J., Qin, W., Li, K., Yu, C., and Jiang, T. (2009). Thick visual cortex in the early blind. J. Neurosci. *29*, 2205–2211.

Jones, E.G. (2000). Microcolumns in the cerebral cortex. Proc. Natl. Acad. Sci. USA *97*, 5019–5021.

Joyner, A.H., J, C.R., Bloss, C.S., Bakken, T.E., Rimol, L.M., Melle, I., Agartz, I., Djurovic, S., Topol, E.J., Schork, N.J., et al. (2009). A common MECP2 haplotype associates with reduced cortical surface area in humans in two independent populations. Proc. Natl. Acad. Sci. USA *106*, 15483–15488.

Kaas, J. (2000). Why is brain size so important: Design problems and solutions as neocortex gets bigger or smaller. Brain Mind *1*, 7–23.

Kanai, R., and Rees, G. (2011). The structural basis of inter-individual differences in human behaviour and cognition. Nat. Rev. Neurosci. *12*, 231–242.

Koch, C. (1984). Cable theory in neurons with active, linearized membranes. Biol. Cybern. *50*, 15–33.

Lewis, J.D., Theilmann, R.J., Sereno, M.I., and Townsend, J. (2009). The relation between connection length and degree of connectivity in young adults: a DTI analysis. Cereb. Cortex *19*, 554–562.

Lutti, A., Thomas, D.L., Hutton, C., and Weiskopf, N. (2013). High-resolution functional MRI at 3 T: 3D/2D echo-planar imaging with optimized physiological noise correction. Magn. Reson. Med. *69*, 1657–1664.

Mountcastle, V.B. (1997). The columnar organization of the neocortex. Brain *120*, 701–722.

Panizzon, M.S., Fennema-Notestine, C., Eyler, L.T., Jernigan, T.L., Prom-Wormley, E., Neale, M., Jacobson, K., Lyons, M.J., Grant, M.D., Franz, C.E., et al. (2009). Distinct genetic influences on cortical surface area and cortical thickness. Cereb. Cortex *19*, 2728–2735.

Purushothaman, G., and Bradley, D.C. (2005). Neural population code for fine perceptual decisions in area MT. Nat. Neurosci. *8*, 99–106.

Rakic, P. (1974). Neurons in rhesus monkey visual cortex: systematic relation between time of origin and eventual disposition. Science *183*, 425–427.

Rakic, P. (1988). Specification of cerebral cortical areas. Science *241*, 170–176.

Ringo, J.L. (1991). Neuronal interconnection as a function of brain size. Brain Behav. Evol. *38*, 1–6.

Sereno, M.I., Dale, A.M., Reppas, J.B., Kwong, K.K., Belliveau, J.W., Brady, T.J., Rosen, B.R., and Tootell, R.B. (1995). Borders of multiple visual areas in humans revealed by functional magnetic resonance imaging. Science *268*, 889–893.

Sporns, O., and Zwi, J.D. (2004). The small world of the cerebral cortex. Neuroinformatics *2*, 145–162.

Sun, W., and Dan, Y. (2009). Layer-specific network oscillation and spatiotemporal receptive field in the visual cortex. Proc. Natl. Acad. Sci. USA *106*, 17986–17991.

Weiskopf, N., Suckling, J., Williams, G., Correia, M.M., Inkster, B., Tait, R., Ooi, C., Bullmore, E.T., and Lutti, A. (2013). Quantitative multi-parameter mapping of R1, PD, MT, and R2 at 3T: a multi-center validation. Front. Neurosci. *7*, 95.

Womelsdorf, T., Schoffelen, J.M., Oostenveld, R., Singer, W., Desimone, R., Engel, A.K., and Fries, P. (2007). Modulation of neuronal interactions through neuronal synchronization. Science *316*, 1609–1612.

Neuron
Supplemental Information

Neural Population Tuning Links Visual Cortical Anatomy to Human Visual Perception

Chen Song, Dietrich Samuel Schwarzkopf, Ryota Kanai, and Geraint Rees

Supplemental Data

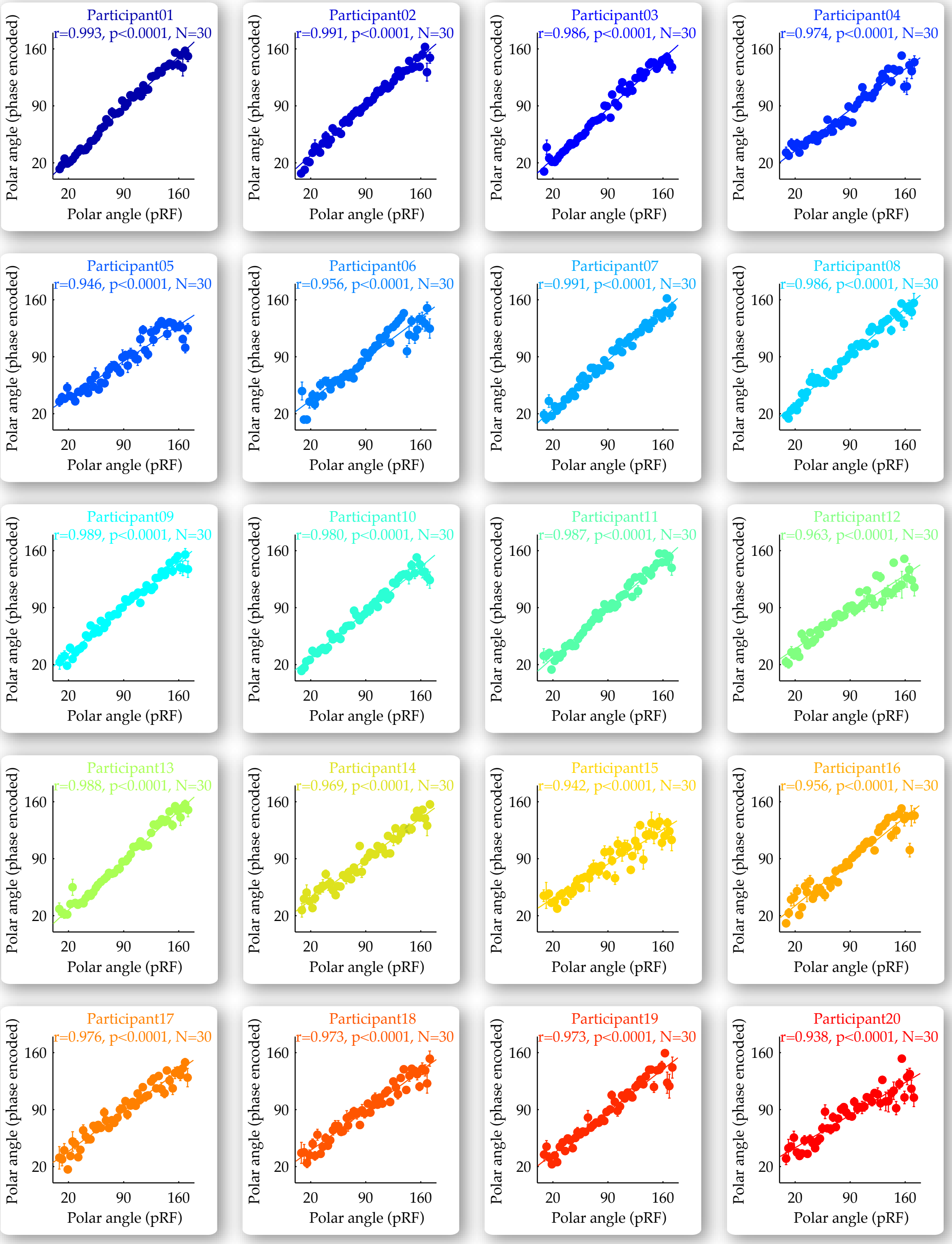

Figure S1. Comparison between Different Retinotopic Mapping Paradigms (Related to Experimental Procedures). Two different retinotopic mapping paradigms, the phase-encoded paradigm and the population-receptive-field paradigm, were used to delineate V1 for each of the twenty participants. The polar angle measures from the two different retinotopic mapping paradigms were plotted against each other on a voxel basis, where voxels responsive to similar polar angle were binned to generate 30 data points for each participant. Based on the voxel-level plot, we calculated the correlation in polar angle measures between the two different retinotopic mapping paradigms. Data points are color coded according to the participant. Statistical values reflect permutation-based Spearman's rank correlation with FWE correction for multiple comparisons.

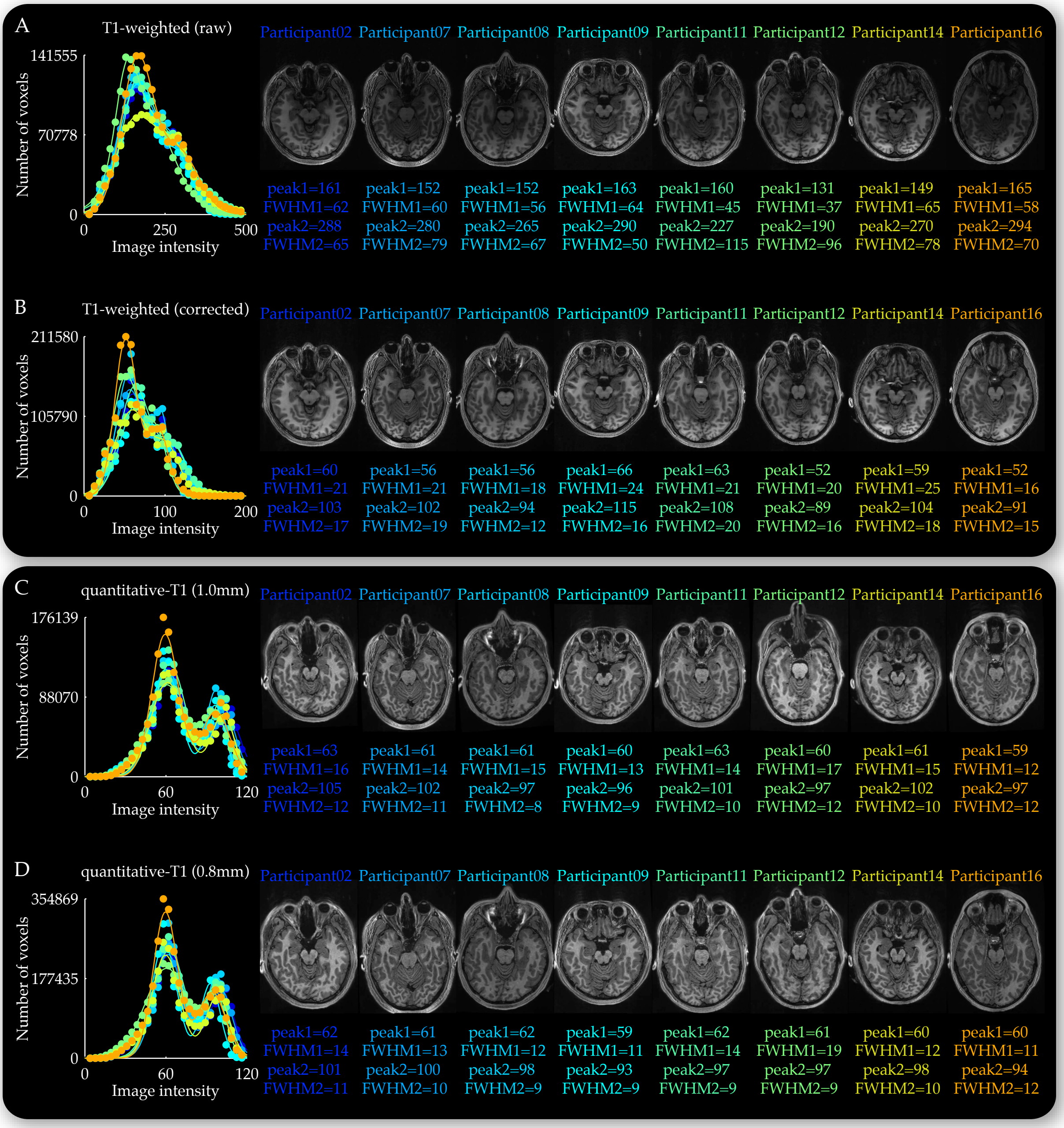

Figure S2. Comparison between T1-weighted MRI Images and Quantitative-T1 MRI Images (Related to Experimental Procedures). Two different MRI sequences, the standard T1-weighted sequence and an advanced quantitative-T1 sequence, were used to collect the structural MRI images for eight of the twenty participants. The distribution of image intensity was plotted on a voxel basis, where voxels with similar intensity value were binned to generate 30 data points for each participant. The double Gaussian fit to the image intensity distribution revealed mixed peaks of white matter and gray matter in the T1-weighted MRI images, not only before (A) but also after (B) non-uniform intensity correction. By contrast, the quantitative-T1 MRI images showed clearly separated peaks of white matter and gray matter with consistent intensity values between 1 mm resolution (C) and 0.8 mm resolution (D). These improvements in intensity homogeneity and tissue contrast from the T1-weighted MRI images to the quantitative-T1 MRI images were further illustrated in the sample cortical slices. Data points are color coded according to the participant. Parameters are derived from the double Gaussian fit to the image intensity distribution.

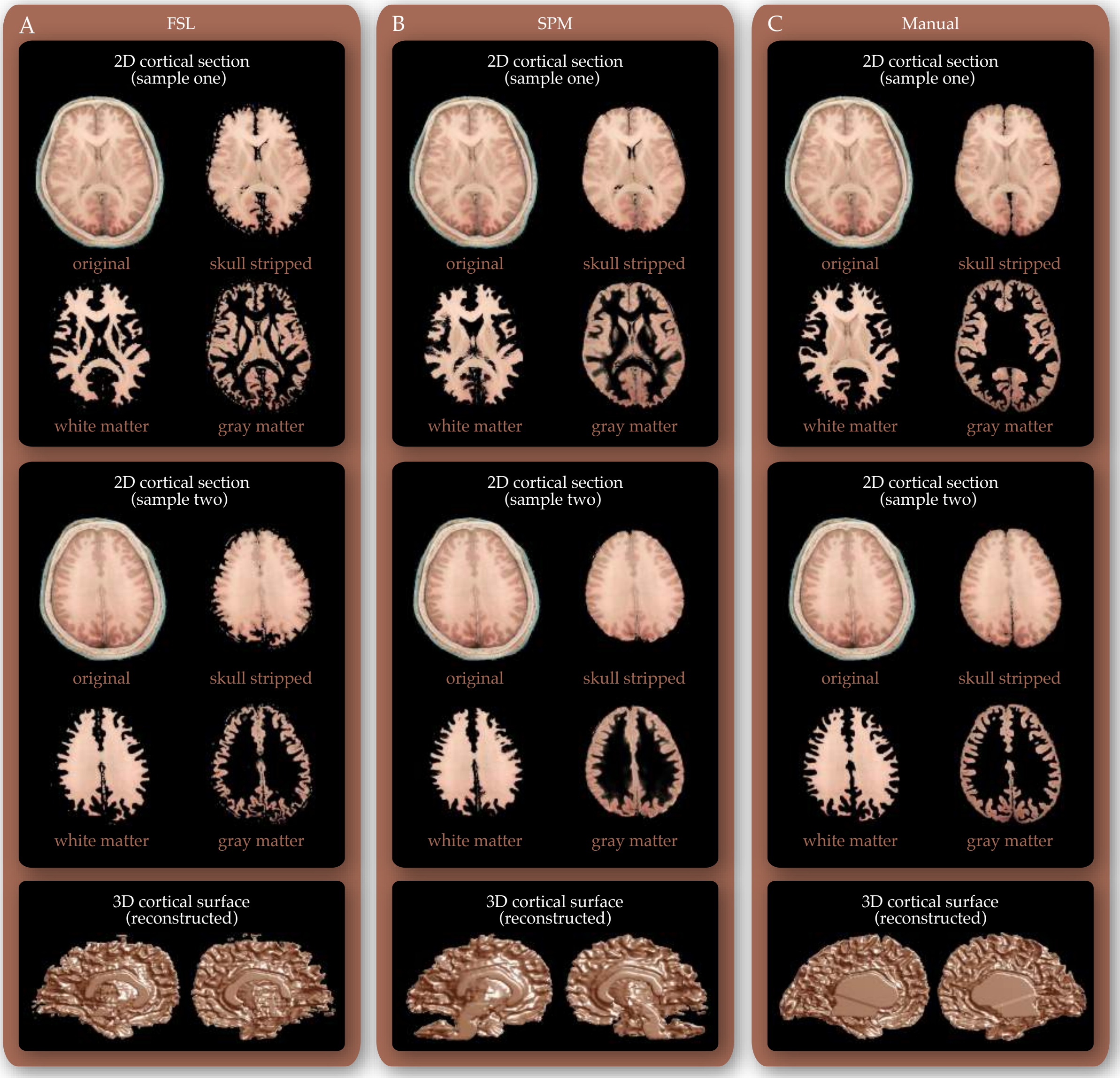

Figure S3. Tissue Segmentation of Histology Data (Related to Experimental Procedures). To build the three-dimensional cortical surface models from the two-dimensional histology images, we segmented each histology image into the white and the gray matter. Our approaches of automatic tissue segmentation, using existing software such as FSL (A) and SPM (B) or using custom-written code, all failed to reach satisfactory accuracy. Consequently, we took the approach of manual tissue segmentation (C). The differences between automatic and manual approaches in the performances of skull stripping, tissue segmentation, and cortical surface reconstruction, were illustrated in the two-dimensional cortical section images and the three-dimensional cortical surface images.

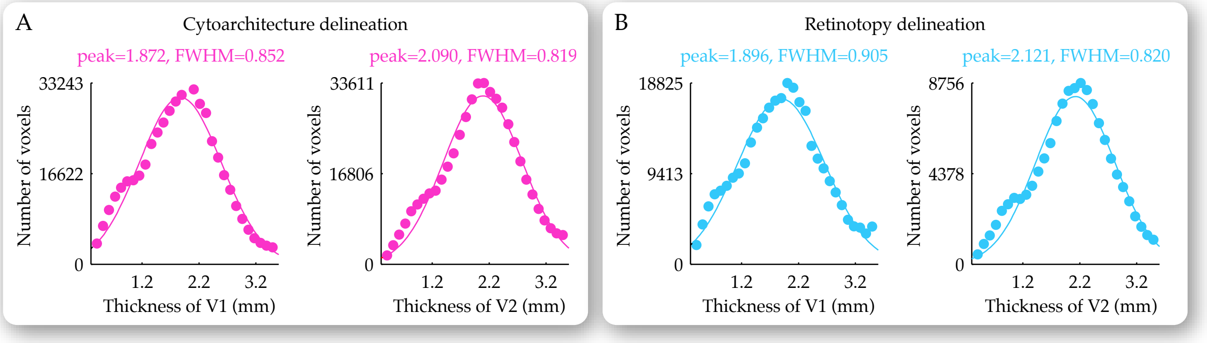

Figure S4. Surface-based Thickness Measure of Histology data (Related to Experimental Procedures). The histology-based measure of visual cortical thickness was acquired for early visual cortices (V1, V2) delineated according to a cytoarchitectonic atlas (A) and according to the average retinotopic map from our fMRI experiments (B), respectively. Based on the delineation of early visual cortices, the cortical thickness at individual visual cortical locations in the histology data was measured as the distance between the white and the pial cortical surfaces. The distribution of histologically-measured visual cortical thickness revealed a substantial degree of intra-individual variability that resembled the observations in MRI-based measure. Data points are color coded according to the delineation method. Parameters are derived from the Gaussian fit to the thickness distribution.

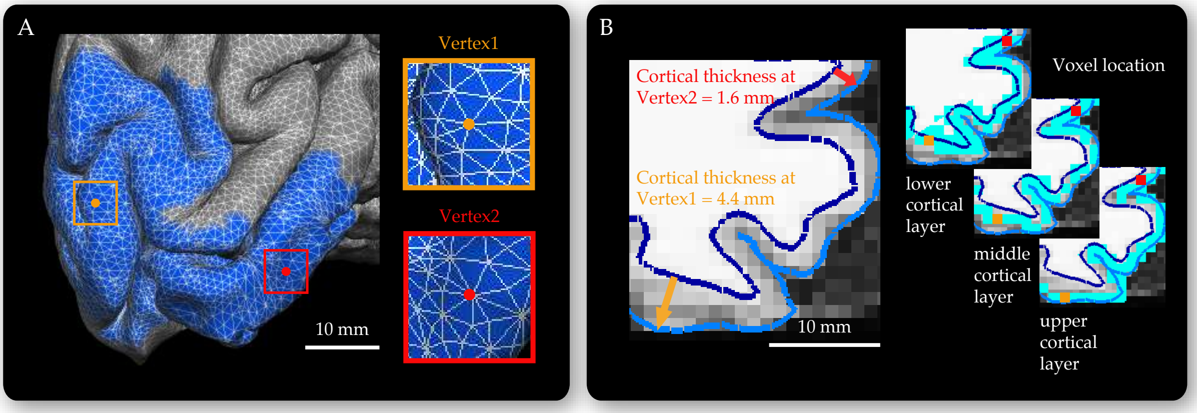

Figure S5. FMRI Spatial Sampling (Related to Experimental Procedures). The triangle-mesh model illustrated the three-dimensional cortical surface reconstruction in a representative participant, where individual vertex of this triangle-mesh model represented a single cortical surface location separable by MRI (A). To assess the influences of fMRI spatial sampling on the measure of neural population tuning, three different voxels, at upper, middle, and lower cortical layers, were sampled for each cortical surface location with high thickness, two different voxels, at upper and lower cortical layers, were sampled for each cortical surface location with medium thickness, and one voxel was sampled for each cortical surface location with low thickness (B). The measure of neural population tuning at individual visual cortical location was compared across voxels at different cortical depth, and the volume of gray matter in individual voxel was compared across different visual cortical locations.

Supplemental Experimental Procedures

1 Participants and apparatus

A group of twenty healthy volunteers gave written informed consent to participate in this study that was approved by the UCL Research Ethics Committee. The participants were young adults (aged 19 to 34, ten females, ten males) with normal or corrected-to-normal vision and no neurological or psychiatric history. All twenty participants took part in the main experiments where we delineated early visual cortices using retinotopic mapping (Sereno et al., 1995), measured visual cortical anatomy using the T1-weighted MRI data, measured neural population tuning for visual field position using the method of population-receptive-field mapping (Dumoulin & Wandell, 2008), and measured perceptual discrimination threshold for visual field position using the psychophysical staircase procedure. Eight (four females, four males) of the twenty participants also took part in the control experiments where we measured visual cortical anatomy using the quantitative-T1 MRI data (Weiskopf et al., 2013), measured fMRI hemodynamic response profile using the visually evoked BOLD responses (Friston et al., 1998; Glover, 1999), and measured fMRI signal-to-noise ratio using the resting state BOLD data (Murphy, Bodurka, & Bandettinia, 2007; Lutti, Thomas, Hutton, & Weiskopf, 2013).

We collected the T1-weighted MRI data at the standard resolution of 1 mm isotropic (TR = 7.92 ms, TE = 2.48 ms, flip angle = 16 degrees, matrix = 256 x 240 x 176), and the quantitative-T1 MRI data at a high resolution of 0.8 mm isotropic as well as the standard resolution of 1 mm isotropic. The protocol for the quantitative-T1 MRI data involved acquisitions of five different MRI signals, and in particular, the proton-density-weighted signals (1 mm resolution: TR = 23.7 ms, TE = 2.2 ms to 19.7 ms by steps of 2.5 ms, flip angle = 6 degrees, matrix = 256 x 240 x 176; 0.8 mm resolution: TR = 25.25 ms, TE = 2.39 ms to 18.91 ms by steps of 2.36 ms, flip angle = 5 degrees, matrix = 320 x 280 x 208), the magnetization-transfer-weighted signals (1 mm resolution: TR = 23.7 ms, TE = 2.2 ms to 14.7 ms by steps of 2.5 ms, flip angle = 6 degrees, matrix = 256 x 240 x 176; 0.8 mm resolution: TR = 29.25 ms, TE = 2.39 ms to 18.91 ms by steps of 2.36 ms, flip angle = 9 degrees, matrix = 320 x 280 x 208), the T1-weighted MDEFT signals (1 mm resolution: TR = 18.7 ms, TE = 2.2 ms to 14.7 ms by steps of 2.5 ms, flip angle = 20 degrees, matrix = 256 x 240 x 176; 0.8 mm resolution: TR = 25.25 ms, TE = 2.39 ms to 18.91 ms by steps of 2.36 ms, flip angle = 29 degrees, matrix = 320 x 280 x 208), the radio-frequency transmit field B1+ signals (1 mm resolution: TR = 500 ms, TE = 18.53 ms / 37.06 ms, flip angle = 90 degrees, matrix = 64 x 48 x 48; 0.8 mm resolution: TR = 500 ms, TE = 19.69 ms / 39.38 ms, flip angle = 90 degrees, matrix = 64 x 48 x 48), and the static magnetic field B0 signals (TR = 1020 ms, TE = 10 ms / 12.46 ms, flip angle = 90 degrees, matrix = 64 x 64 x 64).

We collected the fMRI data at a high spatial resolution of 1.5 mm isotropic, using a 3D EPI sequence with parallel imaging acceleration (Lutti et al., 2013). The data of the main experiments were collected at a standard temporal resolution of 3.2 second volume TR (slice TR = 80 ms, TE = 32.86 ms, flip angle = 20 degrees, field of view = 192 x 192 x 60 mm, 25% oversampling along the encoding slab to avoid wrap-around artifacts). The data of the control experiments were first collected at the same standard temporal resolution of 3.2 second volume TR, and then repeated at a high temporal resolution of 1.52 second volume TR (slice TR = 76 ms, TE = 37.3 ms, flip angle = 15 degrees, field of view = 54 x 192 x 192 mm, 11.1% oversampling along the encoding slab to avoid wrap-around artifacts). The high temporal resolution allowed

fine estimation of fMRI signal properties, but was accompanied by a reduced field-of-view as trade-off. To accommodate the reduced field-of-view, we collected the high temporal resolution fMRI data for the left and the right hemispheres separately in different experimental runs. The fMRI data were preprocessed in SPM8 (http://www.fil.ion.ucl.ac.uk/spm) through bias correction, realignment, unwarping, coregistration, and correction for physiology noise (cardiac activity, respiratory activity, head motion).

The neuroimaging experiments took place in a Siemens Trio 3T MRI scanner with a 32-channel head-coil. Visual stimuli were projected onto a screen (size = 28.6 x 21.5 cm) in the back of the scanner and viewed through a mirror on the head-coil (viewing distance = 85cm). The stimuli covered the visual field extending from fixation to 7.2 degree eccentricity. The psychophysics experiments took place in a dark room where the computer monitor provided the only significant source of light. Visual stimuli were presented on a 22" monitor (size = 41 x 30.6 cm, viewing distance = 67 cm, resolution = 2048 x 1536 pixels, pixel size = 0.017 degree of visual angle) or a 17" monitor (size = 34.2 x 27.5 cm, viewing distance = 3 m, resolution = 1280 x 960 pixels, pixel size = 0.005 degree of visual angle) and viewed through a chin and forehead rest.

2 Delineation of early visual cortices

2.1 Retinotopy-based delineation of early visual cortices

We delineated early visual cortices (V1, V2) non-invasively using the method of phase-encoded retinotopic mapping (Sereno et al., 1995). The mapped visual field covered an eccentricity range from 0.25 to 7.2 degree of visual angle. In the experiment, participants viewed full-contrast flickering checkerboard wedges (radius = 7.2 degree eccentricity) rotating smoothly in clockwise or anti-clockwise direction around a small fixation cross for 10 cycles at a speed of 61.2 seconds per cycle. To maintain participants' attention, at random temporal intervals the checkerboard stimuli underwent a small pattern shift for 200 ms, and participants were asked to indicate whenever this happened with a button press while keeping their eyes fixated at the central cross during the whole experiment. A Fast Fourier Transform was applied to BOLD time series to extract the phase and power at the stimulation frequency. The resulting phase maps were displayed on inflated cortical surfaces reconstructed using FreeSurfer. A statistic map indicating the significance of visual response was calculated by dividing the power at the stimulation frequency with the average power across all frequencies. The polar angle boundaries (representing vertical and horizontal meridians) were delineated manually according to the mirror reversals in the phase map. The eccentricity boundaries (representing 0.25 and 7.2 degree eccentricity) were delineated by thresholding the statistic map at a significance level of $p<0.05$ (uncorrected).

The delineation accuracy of the polar angle boundaries and the eccentricity boundaries were reassured through additional experiments. In particular, to improve the delineation accuracy of polar angle boundaries (representing vertical and horizontal meridians), we compared the polar-angle maps from two different retinotopic mapping experiments using the phase-encoded paradigm (described above) and the population-receptive-field paradigm (described in Supplemental Experimental Procedures Section 4.1), respectively. The comparison was made on a voxel basis for each participant (N = 20). We found that the polar angle values were consistent and correlated between the maps acquired from the two different paradigms (Fig. S1). To improve the delineation accuracy of eccentricity boundaries (representing 0.25 and 7.2 degree

eccentricity), the eccentricity boundaries delineated from the two retinotopic mapping experiments were refined in a third experiment using retinotopic localizer. In the experiment, participants viewed a ring-shaped grating (inner radius = 0.25 degree eccentricity, outer radius = 7.2 degree eccentricity) and a blank screen in a block-design fashion at a speed of 16 seconds per block for 12 blocks per run and 4 runs. Participants maintained their attention and fixation by detecting color change of the central fixation cross. The resulting statistical map was thresholded at $p<0.005$ (uncorrected) to refine the eccentricity boundaries that represented 0.25 and 7.2 degree eccentricity.

2.2 Morphology-based delineation of early visual cortices

As the retinotopy-based delineation covered a part rather than the full extent of early visual cortices, it was potentially confounded by inter-individual differences in the fraction of retinotopy coverage. To address this potential confound, we performed morphology-based delineation where the medial occipital cortex was delineated automatically in Freesurfer according to the cortical folding patterns (Desikan et al., 2006). The morphologically-delineated medial occipital cortex (Freesurfer pericalcarine and cuneus segments) extended along the anterior-posterior axis from the rostral to the caudal ends of the calcarine sulcus, and along the ventral-dorsal axis from the inferomedial end of the calcarine sulcus to the most medial portion of the occipital cortex. Across participants, the morphologically-delineated and the retinotopically-delineated surface area of early visual cortices exhibited correlated inter-individual variability (V1: r = 0.709, $p < 0.001$, N = 20 participants; V2: r = 0.475, $p < 0.05$, N = 20 participants).

The consistency with the morphology-based delineation suggested that the retinotopy-based delineation of early visual cortices was not largely confounded by inter-individual differences in the fraction of retinotopy coverage. To further assess the performance of retinotopic-based delineation, we estimated the fraction of retinotopy coverage for each participant (N = 20), based on the distribution of mapped visual field eccentricity derived from the eccentricity map. This distribution was best fitted with an exponential function $y = ae^{-bx}$. It reflected the percentage of voxels responsive to each visual field eccentricity (Fig. 1B). Given that different voxels were equal in volume, we estimated the retinotopically-delineated part of early visual cortices as the area under the exponential curve from x equaled 0.25 degree eccentricity to x equaled 7.2 degree eccentricity, and the full extent of early visual cortices as the area under the exponential curve from x equaled 0 to x approximated infinite. We found that in both V1 and V2, the retinotopically-delineated part accounted for about three-quarters of the full area. This fraction of retinotopy coverage was rather consistent across participants (V1: mean = 78.3%, std = 3.7%, N = 20; V2: mean = 78.8%, std = 3.3%, N = 20) and did not correlate with inter-individual variability in visual cortical surface area (V1: r = -0.120, p = 0.564, N = 20 participants; V2: r = 0.080, p = 0.768, N = 20 participants). The results reassured us of the reliability of our visual cortical delineation.

3 Visual cortical anatomy

3.1 Influences of MRI analysis software

By applying the surface-based analysis to the T1-weighted MRI data, we measured the thickness at individual visual cortical locations (vertices) and the surface area summed over different visual cortical locations. In the surface-based analysis, the T1-weighted MRI data were preprocessed through skull stripping and non-uniform intensity correction,

after which the pre-processed data were segmented into the white and the gray matter according to intensity-based tissue classification. The white and the gray matter segments were then covered with triangular tessellations to reconstruct the three-dimensional white and pial cortical surfaces in smooth triangle-mesh models. A triangle-mesh model is a collection of vertices (points), edges (connections between vertices), and triangle faces (closet sets of three edges) that defines the shape of a three-dimensional object in geometric modelling. The triangle tessellation was topologically corrected and smoothed using deformable surface algorithm with trilinear interpolation, which allowed the three-dimensional cortical surfaces to be reconstructed at subvoxel accuracy. Based on this three-dimensional cortical surface reconstruction, cortical thickness was computed as the distance between the white and the pial cortical surfaces, and cortical surface area was computed as the summed surface area of triangle faces in a cortical region.

This MRI-based measure of visual cortical anatomy was vulnerable to the confounding influences of data analysis software. To separate the contribution of software specific versus software independent factors, we repeated the analysis in four software, SPM (Ashburner, 2012), Freesurfer (Fischl, 2012), FSL (Jenkinson, Beckmann, Behrens, Woolrich, & Smith, 2012), and MIPAV CBS (http://www.nitrc.org/projects/cbs-tools) (Bogovic, Prince, & Bazin, 2013; Bazin et al., 2013). For each participant, the pre-processing of raw data and the segmentation of cortical tissues were repeated in four different software (SPM, FSL, Freesurfer, MIPAV CBS), after which the reconstruction of cortical surfaces and the computation of cortical thickness were repeated in two different software (Freesurfer, MIPAV CBS).

Across software, we compared the segmentation of cortical tissues, by calculating the standard deviation of the inner cortical boundary (white matter was given the value of one, gray matter the value of zero, the rest of the brain NaN) and that of the outer cortical boundary (gray or white matter was given the value of one, the rest of the brain the value of zero). The standard deviation was calculated on a voxel basis for each participant (N = 20), where the value was 0, or 0.5, or 0.58, if all four, or three, or two software returned the same segmentation result. We also compared the computation of cortical thickness across software, by calculating the correlation in the measure of cortical thickness. Specifically, cortical thickness measured from Freesurfer was plotted against cortical thickness measured from MIPAV CBS, again on a voxel basis. We found that the segmentation standard deviation in occipital lobe (Mazziotta et al., 2001) was low for both the inner cortical boundary (mean = 0.075, std = 0.009, N = 20 participants) and the outer cortical boundary (mean = 0.047, std = 0.009, N = 20 participants). Moreover, the measure of visual cortical thickness was highly correlated between different software ($r = 0.98$, $p < 0.0001$, N = 104158 voxels binned into N = 30 data points according to cortical thickness). Such consistency suggested that the MRI-based measure of visual cortical anatomy was not biased by the specific choice of data analysis software.

3.2 Influences of MRI acquisition sequence

In addition to the concern regarding data analysis software, the MRI-based measure of visual cortical anatomy was potentially vulnerable to the confounding influences of data acquisition sequence. While the T1-weighted MRI sequence we employed is a widely-used standard protocol, the signal in fact represents a combination of magnetic-field-specific and biological-tissue-specific components. Consequently, the T1-weighted MRI images had inhomogenous intensity and low tissue contrast, as illustrated by the mixed peaks of white matter and gray matter in the image intensity histogram (Fig.

S2). This could potentially bias the segmentation of cortical tissues. To address this limitation in quality of the standard T1-weighted MRI images, in control experiments we collected the structural MRI data using a state-of-art quantitative-T1 MRI sequence, at both a high resolution (0.8 mm isotropic voxels, 60 minutes per participant) and a standard resolution (1 mm isotropic voxels, 30 minutes per participant). The quantitative-T1 MRI images were calculated from multiple parametric signals (the proton-density-weighted signals, the magnetization-transfer-weighted signals, the T1-weighted MDEFT signals, the radio-frequency transmit field B1+ signals, the static magnetic field B0 signals) according to the methods developed by (Helms, Dathe, & Dechent, 2008; Weiskopf et al., 2013) including corrections for imperfect spoiling (Preibisch & Deichmann, 2009) and field (B1+, B0) inhomogeneities (Lutti, Hutton, Finsterbusch, Helms, & Weiskopf, 2010; Lutti et al., 2012). Through the consideration of multiple parametric signals, the quantitative-T1 MRI sequence factored out the magnetic-field-specific component and directly reflected the physical property of the underlying biological tissue (Weiskopf et al., 2013). Consequently, the quantitative-T1 MRI images were highly reproducible across different scanning sessions (e.g., 1 mm resolution vs. 0.8 mm resolution) and directly comparable across participants (Fig. S2). Moreover, as illustrated by the clearly separated peaks of white matter and gray matter in the image intensity histogram (Fig. S2), the quantitative-T1 MRI images had high tissue contrast and relatively homogeneous intensity over the cortex. This helped to reduce potential bias in the surface-based analysis.

The surface-based analysis of the quantitative-T1 MRI data were carried out in MIPAV CBS (http://www.nitrc.org/projects/cbs-tools), as the software was developed specifically for high resolution data and was not constrained in the voxel size (Bogovic et al., 2013; Bazin et al., 2013). On a voxel basis, we compared cortical thickness measured from the 1 mm resolution T1-weighted MRI data, the 1 mm resolution quantitative-T1 MRI data, and the 0.8 mm resolution quantitative-T1 MRI data. We found that the measure of visual cortical thickness was consistent across different acquisition sequences and different acquisition resolutions (T1-weighted sequence vs. quantitative-T1 sequence: $r = 0.97$, $p < 0.0001$; 1.0 mm resolution vs. 0.8 mm resolution: $r = 0.97$, $p < 0.0001$; $N = 42538$ voxels binned into $N = 30$ data points according to cortical thickness of 1.0 mm resolution quantitative-T1 MRI data). This observation suggested that although the standard T1-weighted MRI sequence did not offer high image quality, the MRI-based measure of visual cortical anatomy was robust against this limitation.

3.3 Comparison between in-vivo MRI and in-vitro histology

Our control studies reassured us that the MRI-based measure of visual cortical anatomy was not biased by the choice of data analysis software or data acquisition sequence. Nevertheless, while the structural MRI data offer a non-invasive, in-vivo measure of cortical anatomy, the measure is at the same time limited by its indirect nature. In contrast, a direct measure of cortical anatomy (albeit in-vitro) is possible from postmortem histology. Therefore, we further addressed the reliability of our in-vivo MRI measure by comparing it with an in-vitro histology measure derived from postmortem human brain. Conventional analysis of histology data employs a slice-based approach that is constrained by the slice orientation and is consequently limited in sampling coverage. For example, the slice-based measure of cortical thickness is only valid for histology slices orthogonal to the cortical surface. To overcome this limitation, we developed a surface-based histology measure of cortical thickness through reconstructing the three-dimensional triangle-mesh models of the

white and the pial cortical surfaces. The surface-based approach, while demanding, offered a sampling coverage of the full brain that was unconstrained by the slice orientation. This allowed direct comparison in the measure of visual cortical anatomy between the histology data and the structural MRI data.

We applied the surface-based analysis to a dataset of high-resolution (40 μm isotropic pixel), whole-brain (4992 pixel x 3328 pixel), histology images (502 images in total), taken consecutively every 300 μm along the dorsoventral axis of a postmodern human body. The histology data were generously shared by Drs. Yuchun Tang and Shuwei Liu at the Research Center for Sectional and Imaging Anatomy of Shandong University, China, under the approval from the local ethics committee. The postmodern body came from a deceased 38-year-old male who had donated his body for medical research purposes and had no neurological history. After the body was frozen and fixed, the head was dissected from the plane of thyroid cartilage and embedded in blue-stained gelatine. Serial transverse sectioning was performed along the dorsoventral axis using computerised freezing milling technique (milling machine: SKC500, Jinan, China; milling accuracy: 1 μm) (Spitzer, Ackerman, Scherzinger, & Whitlock, 1996). A high-resolution digital camera (Canon EOS 1D MARK II, Japan) was used to take the histology images along with the images of length markers and color charts. The images of length markers and color charts were subsequently used as reference in affine alignment of the histology images. The results of affine alignment were independently checked by two experienced neuroanatomists (Yuchun Tang and Bo Sun) and confirmed through three-dimensional volume reconstruction of the head.

To build the three-dimensional cortical surface models from the two-dimensional histology images, we segmented each histology image into different tissue components. Since performing manual tissue segmentation for a set of 502 images would be labor-intensive and time-consuming, at first we tried to perform automatic tissue segmentation. However, existing algorithms of automatic tissue segmentation were largely based on information of image intensity distribution and were mainly developed for the structural MRI data, yet the histology data differed substantially from the structural MRI data in image intensity distribution. This made MRI-based algorithms unsuitable for analysis of histology data. Indeed, when we applied MRI analysis software (e.g., SPM (Ashburner, 2012), FSL (Jenkinson et al., 2012)) to our histology data, the results of automatic tissue segmentation were rather inaccurate (Fig. S3).

Consequently, we wrote custom code that performed automatic tissue segmentation based on the color information and the consecutive nature of the histology images. We tried several approaches, including using the built-in functions of Mathematica (e.g., ImageForestingComponents, ClusteringComponents), using different saliency detection algorithms (e.g., visual-feature-based, spectral-based), and a combination of both. Nevertheless, neither of these approaches managed to reach satisfactory accuracy. Finally, we resorted to manual tissue segmentation. To improve the segmentation accuracy, we took advantage of the color information of the histology images by performing manual segmentation on the red, green, and blue channel images in addition to the original image. Non-uniform intensity correction (Salvado, Hillenbrand, Zhang, & Wilson, 2006) was also applied, resulting in a total of eight images (corrected/uncorrected red/green/blue/all channel) per transverse section. Based on these images, two researchers (Chen Song and Xiaocheng Sun) performed skull strip and tissue segmentation manually for each transverse section using the software Amira (Stalling, Westerhoff, & Hege, 2005).

Compared to the standard 1 mm resolution of the structural MRI data, the 40 μm in-plane resolution of the histology

data enabled much more accurate tissue segmentation. However, at the same time, the high resolution of the histology data posed challenges for the reconstruction of three-dimensional cortical surface models, because traditional software such as Freesurfer was developed for low resolution data and had constraints regarding voxel size (Fischl, 2012). Nevertheless, the software MIPAV CBS (http://www.nitrc.org/projects/cbs-tools) was developed specifically for high-resolution data and was suitable for our purpose. Using MIPAV CBS, we built the three-dimensional triangle-mesh models of the white and the pial cortical surfaces, from the two-dimensional manual segments of white matter and gray matter. During the analysis, the two-dimensional manual segments were binarized, down-sampled to generate 300 μm isotropic voxels, and refined through three-dimensional topology correction. Based on the three-dimensional cortical surface models, cortical thickness was computed as the distance between the white and the pial cortical surfaces, with the biomechanics of cerebral cortex taken into consideration.

To compare visual cortical thickness measured from the histology data with that measured from the structural MRI data, we delineated the occipital region-of-interest in the histology data according to a cytoarchitectonic atlas (Amunts, Malikovic, Mohlberg, Schormann, & Zilles, 2000) and according to the average retinotopic map from our MRI experiments, respectively. The former approach offered a standardised reference, whereas the later approach improved the comparability between histology-based and MRI-based measure of visual cortical thickness. In both approaches, the regions-of-interests were transformed from the original image space to the histology image space through coregistration and reslice. Regardless of the specific approach, we observed a substantial degree of intra-individual variability in histology-based measure of visual cortical thickness (Fig. S4) that was similar in extent to the MRI-based measure (Fig. 2). Moreover, the dependence of visual cortical thickness on cortical folding and visual field eccentricity that we observed in our structural MRI data was recaptured by our histology data. Specifically, we observed an increase in visual cortical thickness from sulci to gyri for both parafovea (V1: $T = 13.498$, $p < 0.0001$, $N = 87455$ voxels; V2: $T = 18.179$, $p < 0.0001$, $N = 86466$ voxels) and perifovea (V1: $T = 23.822$, $p < 0.0001$, $N = 230387$ voxels; V2: $T = 9.507$, $p < 0.0001$, $N = 50348$ voxels), as well as an increase in visual cortical thickness from parafovea to perifovea for both sulci (V1: $T = 83.929$, $p < 0.0001$, $N = 146834$ voxels; V2: $T = 56.089$, $p < 0.0001$, $N = 72410$ voxels) and gyri (V1: $T = 97.783$, $p < 0.0001$, $N = 171008$ voxels; V2: $T = 49.674$, $p < 0.0001$, $N = 64404$ voxels). This consistency between the MRI-based and the histology-based measures of visual cortical thickness gave us confidence in the application of in-vivo structural MRI data for assessing visual cortical anatomy.

4 Neural population tuning for visual field position

4.1 Measure of position tuning width

Our measure of neural population tuning for visual field position was based on the method of population-receptive-field (pRF) mapping (Dumoulin & Wandell, 2008). In the method, a full-contrast flickered checkerboard bar was presented at 64 different visual field positions, and the BOLD time series of each voxel were deconvolved with hemodynamic response function before fitting with a two-dimensional Gaussian representation of position tuning profile. The two-dimensional Gaussian function quantified the range of visual field positions that the voxel responded to (position tuning width) and the visual field position that the voxel responded strongest to (position tuning peak). This method of

population-receptive-field mapping was conceptually similar to the method of spike-triggered average (reverse correlation) used in extracellular recording for measuring the spatiotemporal receptive field of visual cortical neurons. Both methods relied on the assumption of a linear spatial summation where the response to a stimulus equalled the sum of the responses to the stimulus components. This linearity of spatial summation was observed in BOLD responses from early visual cortices (Hansen, David, & Gallant, 2004), which supported the use of population-receptive-field mapping for estimating neural population tuning.

Each participant (N = 20) took part in two runs of population-receptive-field mapping experiment. In a single experimental run, the checkerboard bar stimulus (width = 1.8 degree of visual angle) moved in the visual field for eight cycles at a speed of sixteen volumes per cycle and one visual field position per volume. The bar stimulus was oriented at one of the four orientations (horizontal, vertical, 45 degree, 135 degree) and moved along the corresponding orthogonal direction (north/south for horizontal bar, west/east for vertical bar, northwest/southeast for 45 degree bar, northeast/southwest for 135 degree bar). The orientation and the moving direction of the bar stimulus were counterbalanced across cycles. A blank screen was inserted into the last quarter of the second, fourth, sixth, and eighth cycle to provide a baseline condition that improved the measurement accuracy. Participants maintained their attention and fixation by detecting color change of the central fixation cross. The BOLD time series of each voxel were deconvolved with hemodynamic response function and then fitted with a two-dimensional Gaussian function f(x_0, y_0, σ) multiplied by the stimulus position function. The peak (x_0, y_0) and the width (σ) of position tuning were estimated for each voxel by varying the parameters x_0, y_0 (limit = 7.2 degree eccentricity) and σ (lower limit = 0.001 degree of visual angle, upper limit = 7.2 degree of visual angle) to find the least square fit. The measure of position tuning width was not confounded by the goodness-of-fit (r = -0.001, p = 0.83) and the voxels with a goodness-of-fit smaller than 15% were excluded in later analysis.

4.2 Influences of fMRI spatial sampling

The voxel-level measure of neural population tuning reflected a combined contribution from the average position tuning width of neurons in the voxel and the heterogeneity in position tuning peak between different neurons in the voxel (Hubel & Wiesel, 1974). To improve the resolution of the measure and minimise intra-voxel heterogeneity in tuning peak, we collected the fMRI data at a high spatial resolution (1.5 mm isotropic voxel) using a 3D EPI sequence with parallel imaging acceleration (Lutti et al., 2013). For tissue volumes as small as 1.5 mm isotropic, the heterogeneity in position tuning peak is smaller than position tuning width of single neurons, and is correlated with the average position tuning width of neurons in the tissue volume (Hubel & Wiesel, 1974). Therefore, the voxel-level measure of position tuning width in effect reflected the average tuning width of neurons in the voxel. Indeed, this voxel-level position tuning width (0.6 ± 0.35 degree of visual angle) measured here in the retinotopically-delineated part of human primary visual cortex was comparable with neural-level position tuning width (0.35 degree of visual angle) in the corresponding part of macaque primary visual cortex (Hubel & Wiesel, 1974).

In addition to minimising intra-voxel heterogeneity in position tuning peak, the high spatial resolution of our fMRI data also contributed to minimising inter-voxel heterogeneity in spatial sampling. To assess the confounding influences of spatial sampling, three different voxels, at upper, middle, and lower cortical layers, were sampled for each visual

cortical location with high cortical thickness, two different voxels, at upper and lower cortical layers, were sampled for each visual cortical location with medium cortical thickness, and one voxel was sampled for each visual cortical location with low cortical thickness (Fig. S5). We found that the measure of position tuning at individual visual cortical location was consistent across voxels at different cortical depth. Moreover, the gray matter volume in individual voxel was homogeneous across different visual cortical locations. These observations suggested that inter-voxel variability in position tuning width was not an artefact of fMRI spatial sampling.

4.3 Influences of fMRI hemodynamic coupling

Despite the improvement in spatial resolution, the fMRI-based measure of neural population tuning was nonetheless still potentially confounded by the temporal lag between neural responses and fMRI signals due to hemodynamic coupling. This potential confounding factor was taken into consideration during the experiment where the BOLD time series of each voxel were deconvolved with hemodynamic response function before fitting with a two-dimensional Gaussian representation of position tuning profile. In the main experiments, we took a classical fMRI analytic approach where a canonical hemodynamic response function $h(t) = (t/5.4)^6 e^{-(t-5.4)/0.9} - 0.35 * (t/10.8)^{12} e^{-(t-10.8)/0.9}$ was used for all participants and all voxels (Friston et al., 1998; Glover, 1999). Since the volume TR (3.2 second) in our experiment was no smaller than the full-width-half-maximum and the time-to-peak of hemodynamic response function in early visual cortices (Siero, Petridou, Hoogduin, Luijten, & Ramsey, 2011), the effective hemodynamic response function, namely the discrete function sampled every volume TR, was a Dirac delta function that temporally lagged position tuning peak for one volume without influencing position tuning width. Therefore, in theory, inter-voxel variability in position tuning width could not be an artefact of hemodynamic variability.

Nevertheless, to improve the reliability of position tuning measure, in control experiments we estimated the hemodynamic response function for individual voxels through acquisition of the visually evoked BOLD responses (Friston et al., 1998; Glover, 1999). We collected the visually evoked BOLD responses, first using the original 3D EPI sequence with the standard temporal resolution (volume TR = 3.2 second), and then using a new 3D EPI sequence with a high temporal resolution (volume TR = 1.52 second). This high temporal resolution allowed a fine estimation of the hemodynamic response function. For the 3D EPI sequences with volume TR of 3.2 and 1.52 second, a full-contrast flickered checkerboard ring (inner radius = 0.25 degree eccentricity, outer radius = 7.2 degree eccentricity) was presented for 3.2 and 3.04 second, followed by a blank screen of 28.8 and 27.36 second, and repeated for 20 and 40 cycles, respectively. Participants maintained their attention and fixation by detecting color change of the central fixation cross. The BOLD time series of each voxel were fitted with a hemodynamic response function $h(t) = (t/t_1)^{(5.6*t_1^2/w_1^2)} e^{-(t-t_1)*(5.6*t_1/w_1^2)} - d * (t/t_2)^{(5.6*t_2^2/w_2^2)} e^{-(t-t_2)*(5.6*t_2/w_2^2)}$, where the peak t_1, t_2 (limit = 12 second), the full-width-half-maximum w_1, w_2 (limit = 30 second), and the dip d (lower limit = 0.2, upper limit = 1) were varied to find the least square fit.

The parameters of this voxel-level optimized hemodynamic response function (mean of t_1 = 5.4, mean of t_2 = 10.7, mean of w_1 = 5.3, mean of w_2 = 7.8, mean of d = 0.36, N = 42538 voxels) were close in value to the parameters of canonical hemodynamic response function (t_1 = 5.4, t_2 = 10.8, w_1 = 5.2, w_2 = 7.4, d = 0.35). Moreover, the parameters exhibited a rather small degree of inter-voxel variability (std of t_1 = 0.006, std of t_2 = 0.029, std of w_1 = 0.008, std of w_2 =

0.027, std of d = 0.001, N = 42538 voxels) that did not co-vary with the measure of position tuning width (t_1: r = -0.107, p = 0.432; t_2: r = -0.045, p = 0.748; w_1: r = -0.049, p = 0.746; w_2: r = -0.030, p = 0.812; d: r = -0.002, p = 0.978; N = 42538 voxels binned into N = 30 data points according to position tuning with). These observations suggested that the canonical hemodynamic response function used in our main experiments was representative of the voxel-level estimation of hemodynamic response functions.

To further improve the reliability of position tuning measure, we reanalysed the data from the population-receptive-field mapping experiment using the voxel-level estimation of hemodynamic response function rather than the canonical hemodynamic response function. We deconvolved the BOLD time series of each voxel with the hemodynamic response function $h(t) = (t/t_1)^{(5.6*t_1^2/w_1^2)} e^{-(t-t_1)*(5.6*t_1/w_1^2)} - d*(t/t_2)^{(5.6*t_2^2/w_2^2)} e^{-(t-t_2)*(5.6*t_2/w_2^2)}$ whose parameters (t_1, t_2, w_1, w_2, d) were estimated from the visually evoked BOLD responses acquired through control experiments. The rest of the analysis followed the same procedure as before, where the deconvolved BOLD time series of each voxel were fitted with a two-dimensional Gaussian representation of position tuning profile f(x_0, y_0, σ) multiplied by the stimulus position function. We found that position tuning width measured using this voxel-level optimized hemodynamic response function was consistent with that measured using canonical hemodynamic response function (r = 0.9355, $p < 0.0001$, N = 42538 voxels binned into N = 30 data points according to position tuning width).

This new analysis suggested that our original analysis of neural population tuning was not biased by inter-voxel variability in hemodynamic response function. Nevertheless, both the new and the original analysis were dependent on the application of hemodynamic response model $h(t, t_1, t_2, w_1, w_2, d)$. To test whether the choice of hemodynamic response model might confound the measure of neural population tuning, we further conducted hemodynamic-model-free analysis where the BOLD time series from the population-receptive-field mapping experiment were deconvolved directly with the time series of visually evoked BOLD responses. We found that position tuning width measured from this model-free analysis and position tuning width measured from the model-based analysis exhibited high level of consistency (r = 0.9394, $p < 0.0001$, N = 42538 voxels binned into N = 30 data points according to position tuning width), suggesting that the measure was not biased by the choice of hemodynamic response model. Together these results suggested that inter-voxel variability in position tuning width more likely reflected neural variability rather than hemodynamic variability.

4.4 Influences of fMRI signal-to-noise ratio

In addition to the confounding influences of hemodynamic coupling, the fMRI-based measure of neural population tuning was also potentially affected by fMRI signal-to-noise ratio. To assess fMRI signal-to-noise ratio, in control experiments we acquired the resting state BOLD data, using the original 3D EPI sequence with standard temporal resolution (volume TR = 3.2 second) as well as the new 3D EPI sequence with a high temporal resolution (volume TR = 1.52 second). A single experiment run lasted for 10 minutes, during which participants maintained awake through eye fixation at a central cross on a black screen, under the monitor of an eye tracker. From the resting state BOLD time series, we calculated fMRI signal-to-noise ratio on a voxel basis, before and after physiological noise correction, respectively. The signal-to-noise ratio was calculated as the mean divided by the standard deviation of the BOLD time series (Murphy et al., 2007; Lutti et al., 2013).

We found that fMRI signal-to-noise ratio did not vary systematically with the measure of position tuning width (r = -0.229, p = 0.114, N = 42538 voxels binned into N = 30 data points according to position tuning width), or with cortical thickness (r = 0.106, p = 0.466, N = 42538 voxels binned into N = 30 data points according to cortical thickness), or with cortical folding (T = 0.061, p = 0.952, N = 42538 voxels). Moreover, while the physiological noise correction significantly improved fMRI signal-to-noise ratio by 35.95%, the degree of improvement did not co-vary with the measure of position tuning width (r = -0.219, p = 0.144, N = 42538 voxels binned into N = 30 data points according to position tuning width), or with cortical thickness (r = -0.004, p = 0.994, N = 42538 voxels binned into N = 30 data points according to cortical thickness), or with cortical folding (T = -0.021, p = 0.983, N = 42538 voxels). These results suggested that inter-voxel variability in position tuning width did not arise from variability in fMRI signal-to-noise ratio.

5 Perceptual discrimination for visual field position

5.1 Measure of position discrimination threshold

We measured the threshold of perceptual discrimination for visual field position using a psychophysical paradigm of spatial comparison, where participants judged the position difference between two concurrently presented stimuli (Vernier stimuli). In a single experimental trial, a pair of collinear bars and a pair of horizontally offset bars were presented in succession on the computer screen with random order (single bar width = 0.15 degree of visual angle, single bar length = 0.6 degree of visual angle, vertical distance between two bars = 0.3 degree of visual angle). The duration of each bar pair was 300 ms and the inter-stimulus-interval was 500 ms. While maintaining central fixation throughout the experiment, participants made an unspeeded forced choice regarding which temporal interval contained the pair of horizontally offset bars. The horizontal position difference between the pair of horizontally offset bars was varied in a 2-up-1-down staircase to assess the threshold at which the performance converged to 70.7% correct. Two consecutive correct answers led to a one-step decrease in the position difference in the next trial, whereas one incorrect answer led to a one-step increase in the position difference. The experiment stopped after eighteen reversals, and position discrimination threshold was calculated as the horizontal position difference averaged over the last ten reversals.

To test the reliability of position discrimination measure, in a separate experiment we employed a psychophysical paradigm of temporal comparison, where participants judged the position difference between two sequentially presented stimuli. In a single experimental trial, two bars were presented in succession on the computer screen where the duration of each bar was 300 ms and the inter-stimulus-interval was 500 ms (bar width = 0.15 degree of visual angle, bar length = 1.5 degree of visual angle). While maintaining central fixation throughout the experiment, participants made an unspeeded forced choice regarding whether the second bar, compared with the first bar, was moved horizontally rightwards or leftwards. The horizontal position difference between the two bars was varied in a 2-up-1-down staircase to assess the threshold at which the performance converged to 70.7% correct. For each participant (N = 20), we measured position discrimination threshold at central fixation (zero eccentricity) using the two different paradigms. We found that position discrimination threshold measured from the spatial and the temporal comparison paradigm was correlated across participants (r = 0.652, $p < 0.01$, N = 20 participants), suggesting that the measure was robust to the psychophysical paradigm.

5.2 Cortical projection of position discrimination threshold

To acquire a cortical map of perceptual acuity, we measured position discrimination threshold at a set of non-overlapping, evenly distributed visual field positions, using the psychophysical paradigm of spatial comparison. For each individual visual field position, we sought to acquire as accurate a measure of position discrimination threshold as possible such that the minimum position difference in the visual stimuli was smaller than participants' minimum position discrimination threshold. At the same time, we hoped to achieve a large visual field coverage that was at least no smaller than the visual field coverage in our fMRI experiments (i.e., central 7.2 degree eccentricity). However, there was a natural trade-off between the spatial resolution and the visual field coverage of any visual display. Therefore, we applied two different setup, one 17" monitor that offered a high spatial resolution (pixel size = 0.005 degree of visual angle) yet a limited visual field coverage (radius = 2.4 degree of visual angle), and one 22" monitor that offered a large visual field coverage (radius = 12.9 degree of visual angle) yet a limited spatial resolution (pixel size = 0.017 degree of visual angle). Balancing the available setup with our needs, we measured position discrimination thresholds at thirteen non-overlapping visual field positions covering three eccentricities (0, 4.7, 6.7 degree) and six polar angles (45, 90, 135, 225, 270, 315 degree). The measure at each visual field position was carried out in an independent experiment. We used the 17" monitor to measure position discrimination threshold at the central visual field (zero eccentricity) and the 22" monitor to measure position discrimination threshold at the peripheral visual field (4.7, 6.7 degree eccentricity).

The measure of position discrimination threshold was then projected onto early visual cortices to generate a cortical map of perceptual acuity. Specifically, the measure at each visual field position was attributed to the corresponding visual cortical locations (vertices) whose extent of position tuning overlapped with that visual field position. In the rare case where more than one position discrimination threshold was attributed to a single visual cortical location, the average of these thresholds was used. When determining whether the extent of position tuning overlapped with a visual field position, we applied four different criteria. The effective visual field position was taken as the area covering either the ends of bar stimuli (extending 0.2 degree of visual angle from the ends of the two bars) or the entire bar stimuli (extending 0.75 degree of visual angle from the stimulus center). The effective position tuning was cut-off at either 2.5σ (where the activity fell to 5% of the maximum activity) or 1.5σ (where the activity fell to 1/e of the maximum activity). Under the four different criteria, the cortical projection of position discrimination threshold covered 55%, 45%, 70%, 60% of the cortical locations in V1, respectively, and 60%, 50%, 90%, 80% of the cortical locations in V2, respectively. In the main manuscript, we reported the results where the effective visual field position was taken as the area covering the ends of bar stimuli and the effective position tuning was cut-off at 2.5σ. However, the specific criterion applied did not affect the results.

References

Amunts, K., Malikovic, A., Mohlberg, H., Schormann, T., & Zilles, K. (2000). Brodmann's areas 17 and 18 brought into stereotaxic space-where and how variable? *Neuroimage*, 66-84.

Ashburner, J. (2012). Spm: A history. *NeuroImage*, *62*, 791-800.

Bazin, P. L., Weiss, M., Dinse, J., Schafer, A., Trampel, R., & Turner, R. (2013). A computational framework for ultra-high resolution cortical segmentation at 7 tesla. *Neuroimage*, 10.1016.

Bogovic, J. A., Prince, J. L., & Bazin, P. L. (2013). A multiple object geometric deformable model for image segmentation. *Computer Vision and Image Understanding*, *117*, 145-157.

Desikan, R. S., Segonne, F., Fischl, B., Quinn, B. T., Dickerson, B. C., Blacker, D., et al. (2006). An automated labeling system for subdividing the human cerebral cortex on mri scans into gyral based regions of interest. *Neuroimage*, *31*, 968-980.

Dumoulin, S. O., & Wandell, B. A. (2008). Population receptive field estimates in human visual cortex. *Neuroimage*, *39*, 647-660.

Fischl, B. (2012). Freesurfer. *NeuroImage*, *62*, 774-781.

Friston, K. J., Fletcher, P., Josephs, O., Holmes, A., Rugg, M. D. M., & Turner, R. (1998). Event-related fmri: characterizing differential responses. *Neuroimage*, *7*, 30-40.

Glover, G. H. (1999). Deconvolution of impulse response in event-related bold fmri. *Neuroimage*, *9*, 416-429.

Hansen, K. A., David, S. V., & Gallant, J. L. (2004). Parametric reverse correlation reveals spatial linearity of retinotopic human v1 bold response. *Neuroimage*, *23*, 233-241.

Helms, G., Dathe, H., & Dechent, P. (2008). Quantitative flash mri at 3t using a rational approximation of the ernst equation. *Magnetic Resonance in Medicine*, *59*, 667-672.

Hubel, D. H., & Wiesel, T. N. (1974). Uniformity of monkey striate cortex: a parallel relationship between field size, scatter, and magnification factor. *The Journal of comparative neurology*, *158*, 295-306.

Jenkinson, M., Beckmann, C. F., Behrens, T. E., Woolrich, M. W., & Smith, S. M. (2012). Fsl. *NeuroImage*, *62*, 782-790.

Lutti, A., Hutton, C., Finsterbusch, J., Helms, G., & Weiskopf, N. (2010). Optimization and validation of methods for mapping of the radiofrequency transmit field at 3t. *Magnetic Resonance in Medicine*, *64*, 229-238.

Lutti, A., Stadler, J., Josephs, O., Windischberger, C., Speck, O., Bernarding, J., et al. (2012). Robust and fast whole brain mapping of the rf transmit field b1 at 7t. *PLoS One*, *7*, e32379.

Lutti, A., Thomas, D. L., Hutton, C., & Weiskopf, N. (2013). High-resolution functional mri at 3 t: 3d/2d echo-planar imaging with optimized physiological noise correction. *Magnetic Resonance in Medicine*, 1657-1664.

Mazziotta, J., Toga, A., Evans, A., Fox, P., Lancaster, J., Zilles, K., et al. (2001). A probabilistic atlas and reference system for the human brain: International consortium for brain mapping (icbm). *Computer Vision and Image Understanding*, *356*, 1293-1322.

Murphy, K., Bodurka, J., & Bandettinia, P. A. (2007). How long to scan? the relationship between fmri temporal signal to noise ratio and necessary scan duration. *Neuroimage*, 565-574.

Preibisch, C., & Deichmann, R. (2009). Influence of rf spoiling on the stability and accuracy of t1 mapping based on spoiled flash with varying flip angles. *Magnetic Resonance in Medicine*, *61*, 125-135.

Salvado, O., Hillenbrand, C., Zhang, S., & Wilson, D. L. (2006). Method to correct intensity inhomogeneity in mr images for atherosclerosis characterization. *IEEE Transactions on Medical Imaging*, *25*, 539-552.

Sereno, M. I., Dale, A. M., Reppas, J. B., Kwong, K. K., Belliveau, J. W., Brady, T. J., et al. (1995). Borders of multiple visual areas in human revealed by functional magnetic resonance imaging. *Science*, *268*, 889-893.

Siero, J. C. W., Petridou, N., Hoogduin, H., Luijten, P. R., & Ramsey, N. F. (2011). Cortical depth-dependent temporal dynamics of the bold response in the human brain. *Journal of Cerebral Blood Flow Megabolism*, *31*, 1999-2008.

Spitzer, V., Ackerman, M. J., Scherzinger, A. L., & Whitlock, D. (1996). The visible human male: a technical report. *Journal of the American Medical Informatics Association*, *3*, 118-130.

Stalling, D., Westerhoff, M., & Hege, H. C. (2005). Amira: A highly interactive system for visual data analysis. *The Visualization Handbook (Elsevier)*, 749-767.

Weiskopf, N., Suckling, J., Williams, G., Correia, M. M., Inkster, B., Tait, R., et al. (2013). Quantitative multi-parameter mapping of r1, pd(*), mt, and r2(*) at 3t: a multi-center validation. *Frontiers in Neuroscience*, *78*, doi: 10.3389.
